# Supplementary material for: Specific gut microbiome members are associated with distinct immune markers in pediatric allogeneic hematopoietic stem cell transplantation
Source: Microbiome. 2019 Sep 13;7:131. doi: 10.1186/s40168-019-0745-z (PMC6744702; doi:10.1186/s40168-019-0745-z)
Supplement: Supplementary file 1 — Table S1. Clinical patient characteristics. General patient characteristics, conditioning regimens, complications, and outcomes for the pediatric cohort (n = 37) and the subcohort (n = 30) for which the intestinal microbiome was characterized. Abbreviations: HLA, human leukocyte antigen; TBI, total body irradiation; CY, Cyclophosphamide; VP16, Etoposide; BU, Busulfan; MEL, Melphalan; GvHD, graft-versus-host disease. (PDF 379 kb) [file 40168_2019_745_MOESM1_ESM.pdf]

Characteristics		Number of patients (all)	Percentage of all patients (%)	Number of patients (Sub-cohort for which the microbiota was characterized)	Percentage of sub-cohort (%)
**Transplant recipients**		37	100	30*	81.1
**Average recipient age in years**		8.2 (Range: 1.1-18.0)	NA	7.8 (Range: 1.1-16.5)	NA
**Intestinal microbiome characterized**	**At 1-2 timepoints**	NA	NA	8	26.7
	**At 3-4 timepoints**	NA	NA	15	50
	**At 5-6 timepoints**	NA	NA	8	26.7
**Patient sex**	**Female**	15	40.5	12	40
	**Male**	22	59.5	18	60
**Disease at transplantation**	**malignant hematologic diseases**	23	62.2	17	56.7
	**Severe aplastic anemia**	5	13.5	5	16.7
	**Other benign disorders including immunodeficiencies**	9	24.3	8	26.7
**Donor type**	**HLA-matched sibling**	7	18.9	6	20
	**HLA-matched unrelated donor (9/10 or 10/10 match)**	27	73	22	73.3
	**HLA-mismatched umbilical cord blood donor (8/10 match)**	3	8.1	2	6.7
**Stem cell source**	**Bone marrow**	30	81.1	25	83.3
	**Umbilical cord blood**	4	10.8	3	10
	**Peripheral blood**	2	5.4	1	3.3
	**Bone marrow and umbilical cord blood**	1	2.7	1	3.3
**Conditioning regimen**	**TBI + CY / TBI + VP16**	10	27.1	10	33.3
	**Combinations of BU, CY, VP16 and MEL**	18	48.7	12	40
	**Fludarabine-based conditioning**	9	24.3	8	26.7

**Anti-thymocyte globulin treatment**		28	75.7	23	76.7
**Antibiotics pre- and post-HSCT**		37	100	30	100
**Sex mismatch** (female donor to male recipient)		4	10	3	10
**Acute GvHD**	**Grade 0-I**	26	70.3	22	73.3
	**Grade II-IV**	11	29.2	8	26.7
**Chronic GvHD within 24 months**		8	18.9	5	16.7
**Bacterial infections**	**At least one registered bacterial infection**	25	67.6	19	63.3
	**No registered bacterial infection**	12	32.4	11	36.7
**Overall Survival****	**Alive**	30	81.1	24	80
	**Dead**	7	18.9	6	20
**Relapse of primary malignancy**		6	16.2	7	23.3
**Non-relapse mortality**		2	5.4	1	3.3
**Re-transplantation**		3	8.1	4	13.3

*29/37 +1, one patient's microbiome was characterized but for which hBD2 was not measured

** Mean follow-up time after HSCT: 62.1 months (range: 48.9 – 75.8 months)
