# Supplementary figures and images for: Specific gut microbiome members are associated with distinct immune markers in pediatric allogeneic hematopoietic stem cell transplantation
Source: Microbiome. 2019 Sep 13;7:131. doi: 10.1186/s40168-019-0745-z (PMC6744702; doi:10.1186/s40168-019-0745-z)

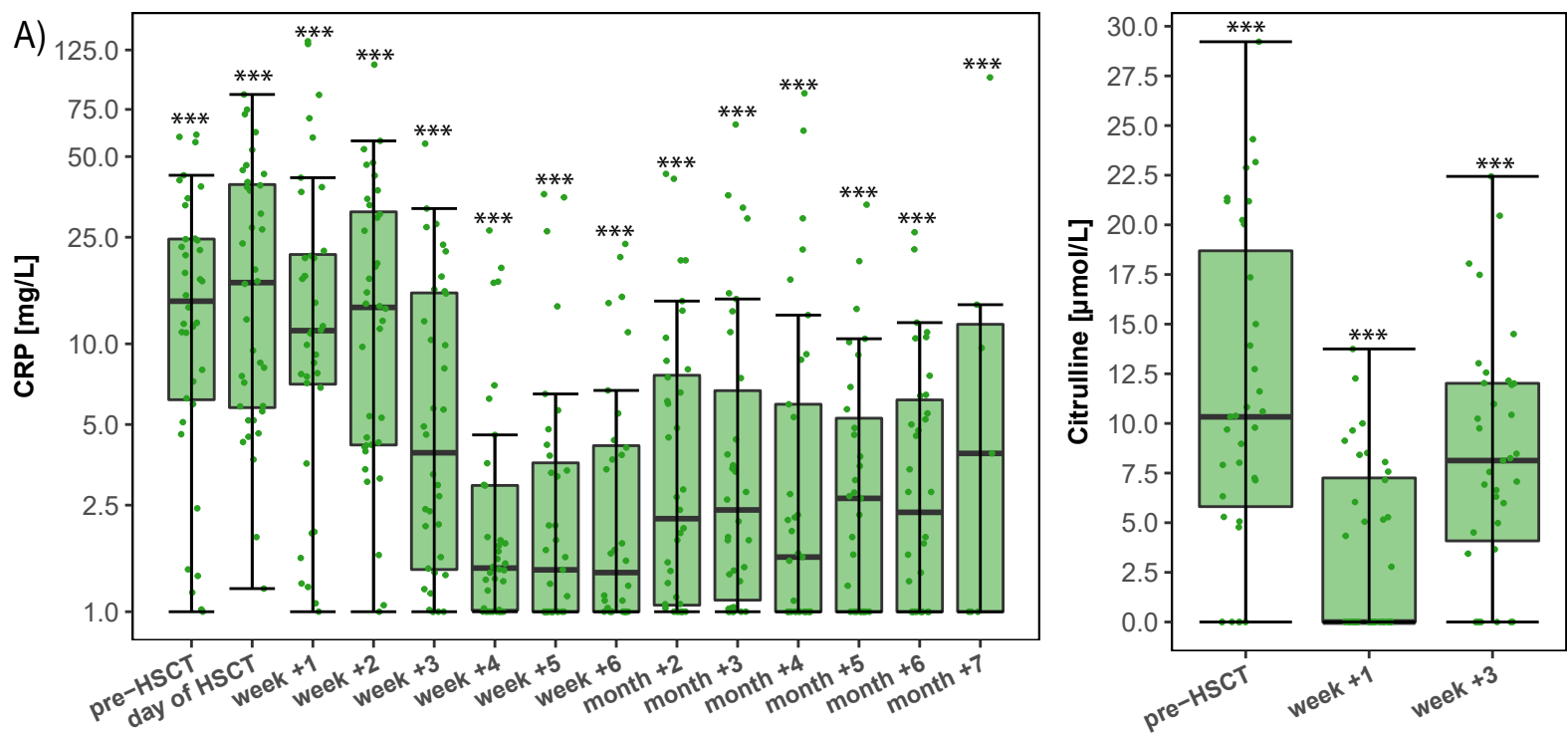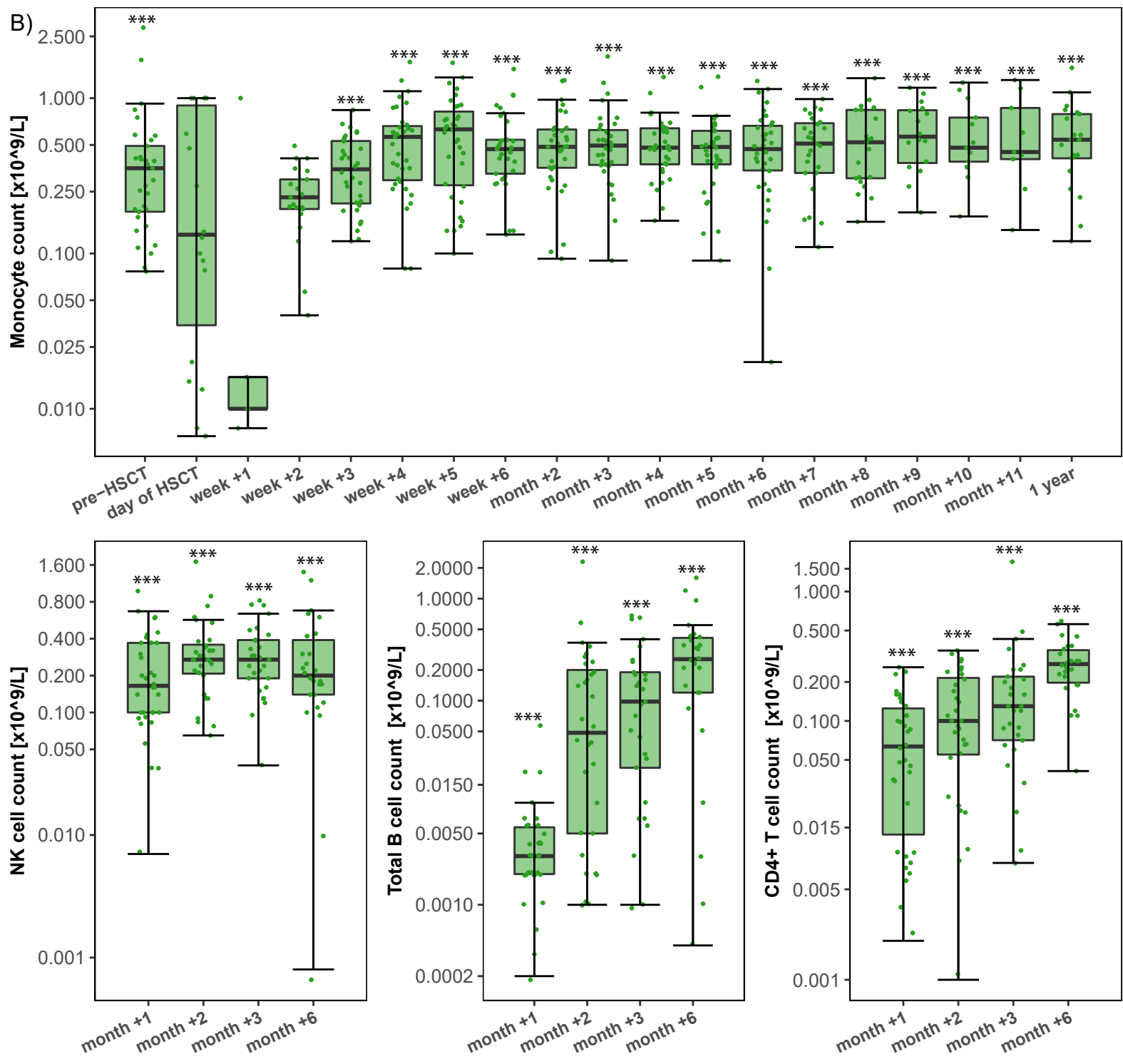

Supplement: Supplementary file 2 — Figure S1. Temporal patterns of immune markers and immune cells in HSCT patients. (A) C-reactive protein (CRP) and plasma citrulline levels in HSCT patients (n = 37) over time. CRP levels were significantly higher prior to HSCT and until week +2 compared to all following time points, e.g. at the day of HSCT (median: 16.93 mg/L, range: 1.22 - 85.28 mg/L) compared to week +3 (median: 3.92 mg/L, range: 1.22 - 55.89 mg/L) (P < 0.001). Plasma citrulline levels were significantly lower in week +1 compared to pre-HSCT (P < 0.001) and week +3 (P < 0.001). (B) Immune cell counts in HSCT patients over time. Monocyte counts are depicted at more time points than indicated in Figure 1a, because not all time points were included into further analyses (see Methods). NK cell counts were higher in months +2 to +6 compared to in month +1 (P < 0.001). B cell counts as well as CD4+ T cell counts increased steadily from month +1 to month +6 (P < 0.001). Y -axes in all plots, except for citrulline, were log10-transformed for better visualization. Zeros were replaced with 1 to avoid undefined values on the log-transformed axes. Asterisks indicate whether the component at each respective time point was significantly different from any of the other time points (showing the maximum significance level). * P < 0.05, ** P < 0.01 and *** P < 0.001. (PDF 419 kb) [file 40168_2019_745_MOESM2_ESM.pdf]

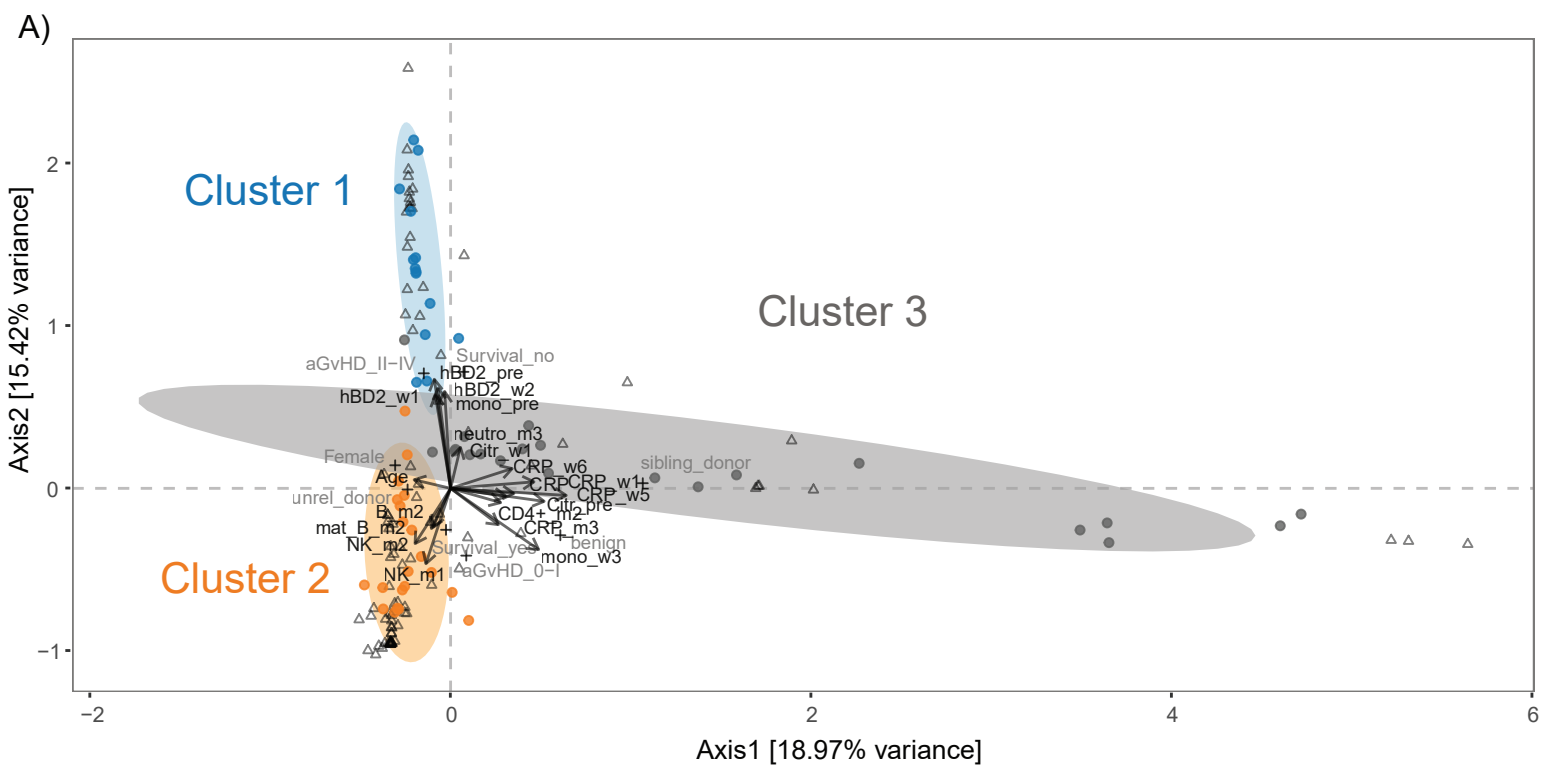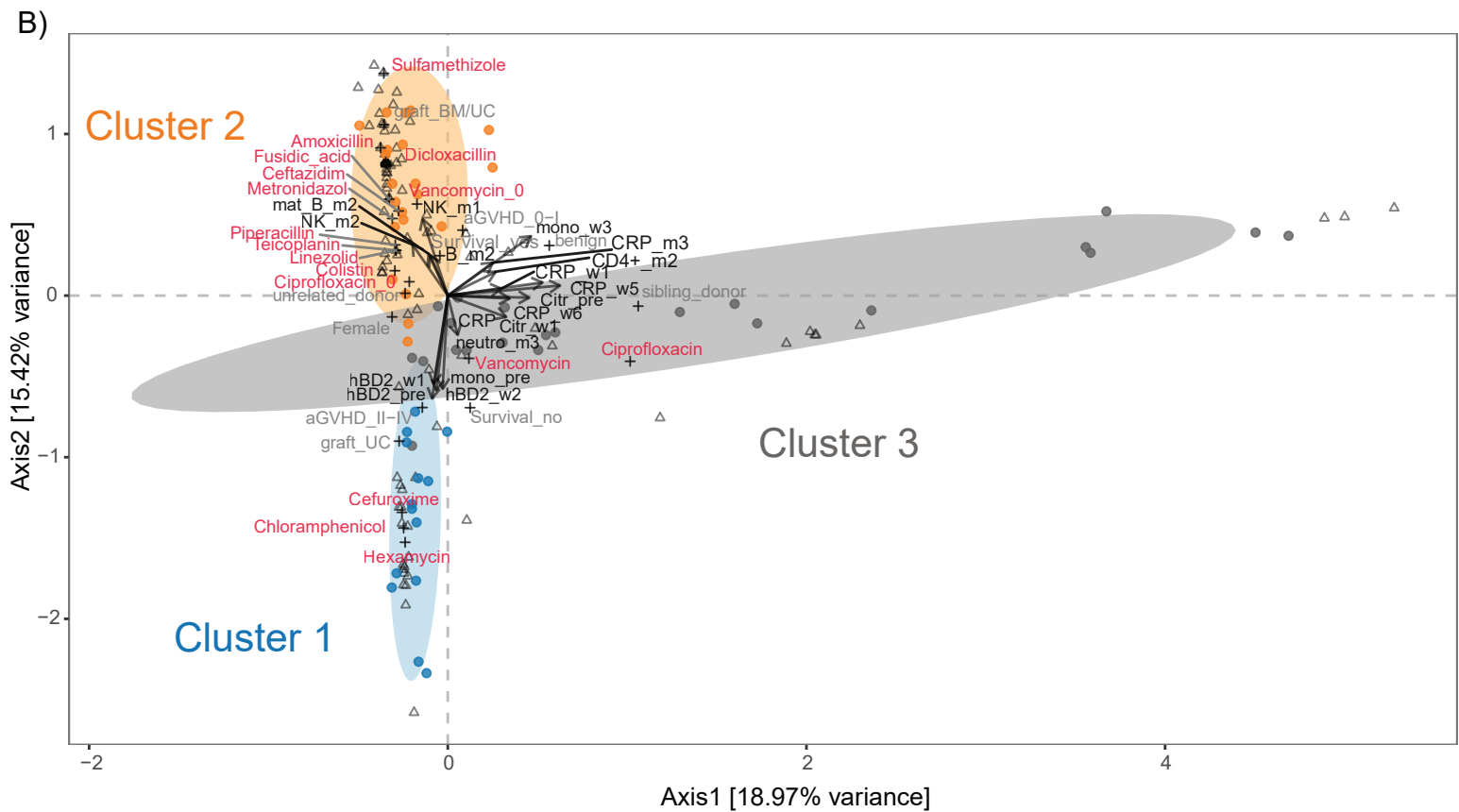

Supplement: Supplementary file 6 — Figure S3. Canonical correspondence analysis (CCpnA) of immune markers and intestinal bacterial taxa in patients undergoing HSCT. Triplots showing dimension 1 and 2 of the CCpnA that includes continuous clinical variables (arrows), categorical variables (+), and OTUs (circles). Samples are depicted as triangles. OTUs with a correlation of >0.2/<-0.2 in the sPLS analysis were included in the CCpnA model. Only the variables and OTUs with a score >0.2/<-0.2 in at least one CCpnA dimension are shown. The OTUs in the CCpnA plots are colored according to the cluster they were affiliated with in the sPLS-based hierarchical clustering analysis, and the ellipses present an 80% confidence interval, assuming normal distribution. (A) Full size visualization corresponding to the CCpnA model shown in Figure 4. Plot dimensions correspond to the explained variances of each component. (B) CCpnA including antibiotic treatment at time points simultaneous to microbiome characterization. Antibiotics were added as categorical variables. Depiction of the antibiotic’s name (in red) indicates administration of the particular antibiotic, and the extension “_0” indicates no administration of the respective antibiotic. Abbreviations of variables are the same as in Figure 2. Further abbreviations: graft_BM: stem cell source bone marrow; graft_UC: stem cell source umbilical cord blood. (PDF 1356 kb) [file 40168_2019_745_MOESM6_ESM.pdf]

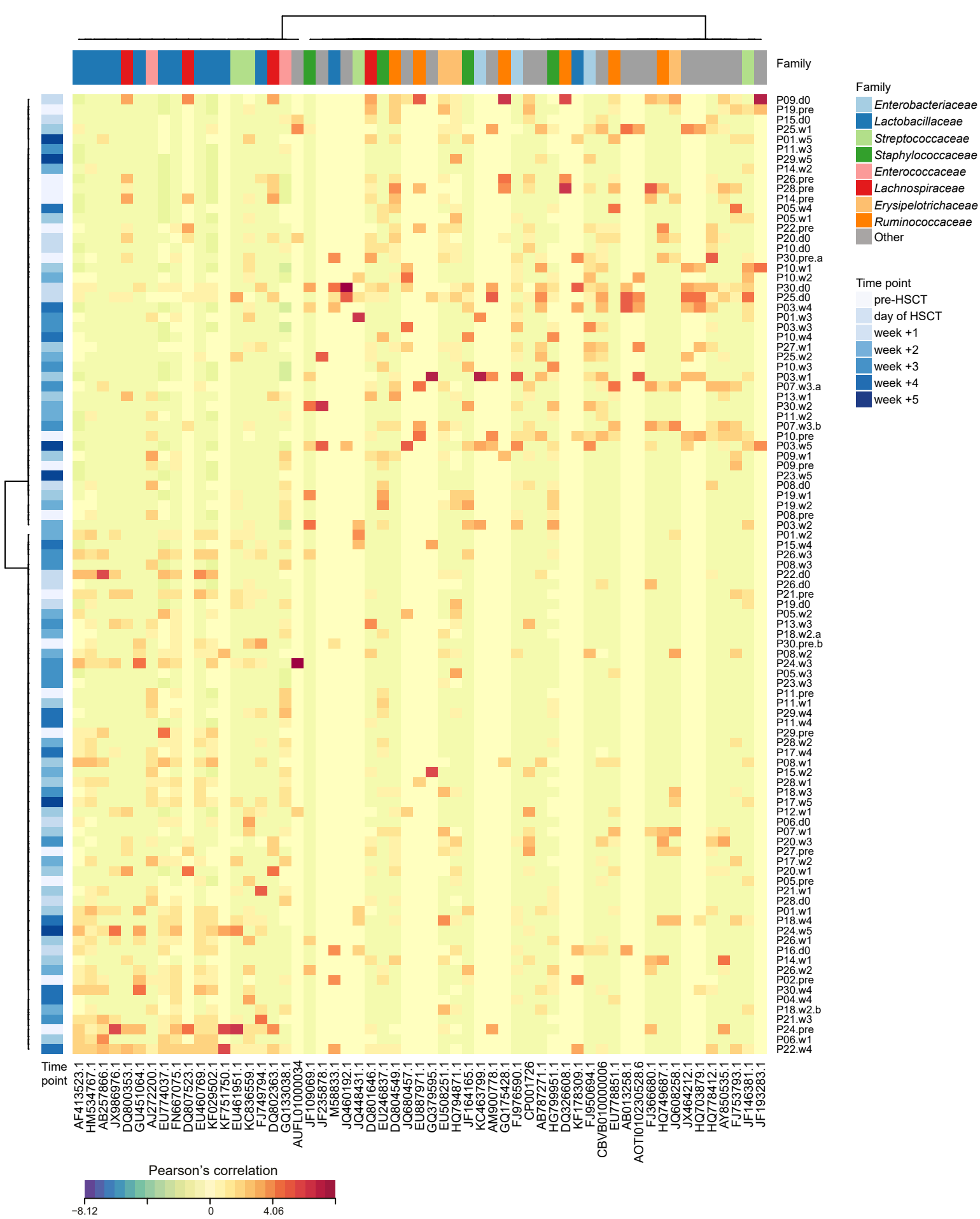

Supplement: Supplementary file 7 — Figure S4. Clustered image map (CIM) of OTU abundances by patient in the first two sPLS dimensions. Hierarchical clustering of OTU abundances (bottom) and patients’ fecal samples (right) (clustering method: complete linkage, distance method: Pearson’s correlation) was performed within the mixOmics cim() function based on the sPLS regression model. High abundance of an OTU in a sample is represented as positive correlation in the map (red) and low abundance as negative correlation (blue). The sampling time points of the fecal samples are displayed in the side bar on the left (blue gradient from pre-HSCT time point (light blue) to week +5 post-HSCT (dark blue)). The top side bar shows taxonomic information on family level. Sample names on the right indicate patient (P) and time point (pre: pre-HSCT, d0: day of HSCT, w: week). An “a” or “b” indicates that two samples were collected from the respective patient at the same time point, but on two different days. (PDF 491 kb) [file 40168_2019_745_MOESM7_ESM.pdf]

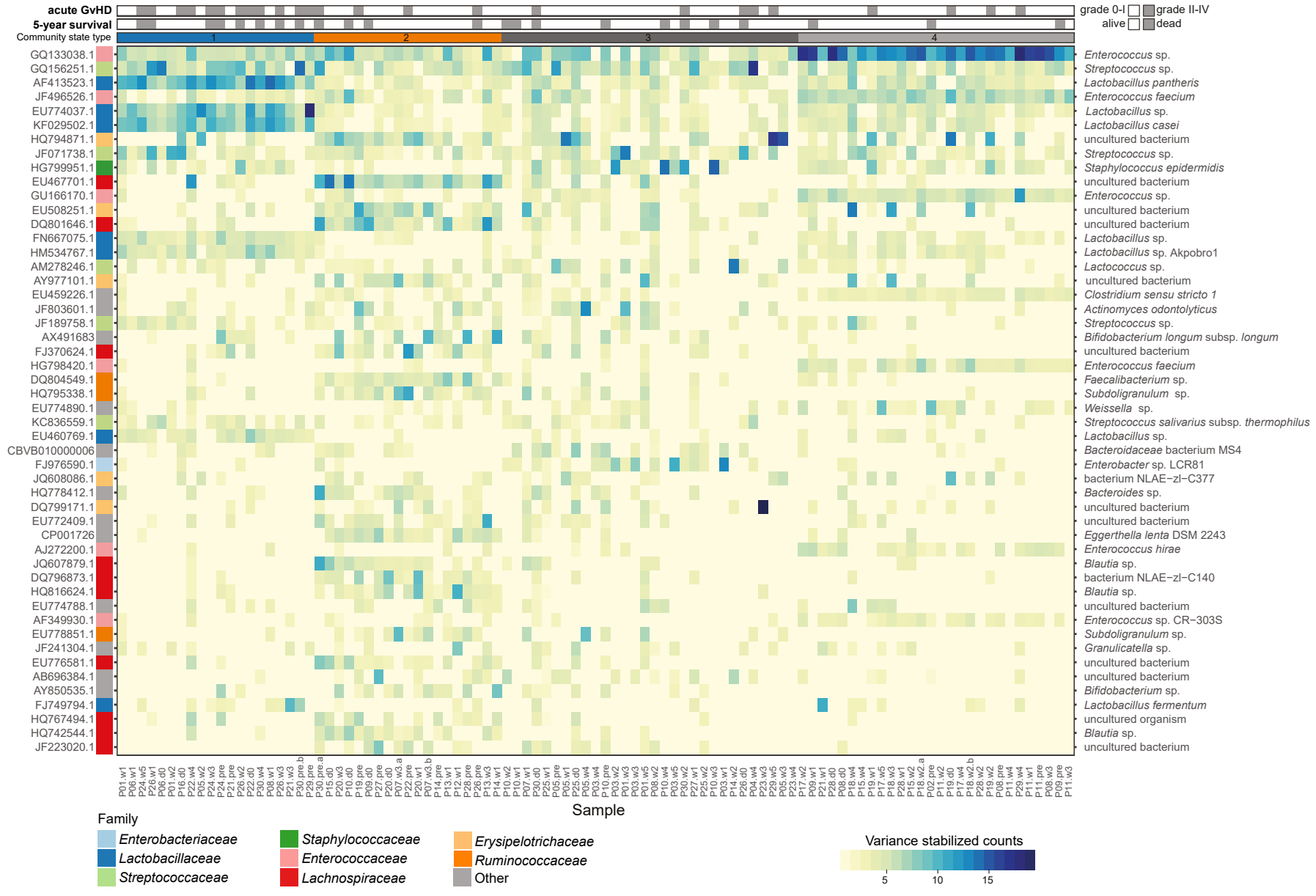

Supplement: Supplementary file 8 — Figure S5. Community state types and gut microbial patterns. Heat map of variance stabilized counts of the 50 most abundant OTUs of the intestinal microbiome over all samples, grouped into community state types (CSTs). Based on their OTU-composition, samples were assigned to community state types (CSTs) by partitioning around medoid (PAM) clustering using Bray-Curtis distance. The optimal number of clusters (k = 4) was estimated from the gap statistic and Silhouette width validation. Members of the Lactobacillaceae family dominated the abundance profiles within CST 1. CST 2 exhibited domination by Lachnospiraceae, Erysipelotrichaceae and Ruminococcaceae members. Enterobacteriaceae, Streptococcaceae and Staphylococcacea were characteristic for CST 3. CST 4 was characterized by a high abundance of Enterococcaceae. Average Silhouette width was s(i) = 0.16 (range: -0.02 – 0.36), with CST 1 and CST 4 being the best defined clusters (s(i) = 0.23 and 0.36, respectively). A Silhouette coefficient s(i) close to 1 indicates appropriate clustering of the respective samples. Sample names at the bottom indicate patient (P) and time point (pre: pre-HSCT, d0: day of HSCT, w: week). An “a” or “b” indicates that two samples were collected from the respective patient at the same time point, but on two different days. (PDF 516 kb) [file 40168_2019_745_MOESM8_ESM.pdf]
