# Supplementary material for: Specific gut microbiome members are associated with distinct immune markers in pediatric allogeneic hematopoietic stem cell transplantation
Source: Microbiome. 2019 Sep 13;7:131. doi: 10.1186/s40168-019-0745-z (PMC6744702; doi:10.1186/s40168-019-0745-z)
Supplement: Supplementary file 3 — Figure S2. Workflow of the statistical analysis approach. The diagram displays the major steps of the statistical analyses and their dependencies. Multivariate analyses (blue box) constitute the main approach, especially the multi-table analyses and clustering analyses (green box). To unravel the complexity of the multivariate analyses, these were supplemented with univariate analyses (upper grey box). (PDF 911 kb) [file 40168_2019_745_MOESM3_ESM.pdf]

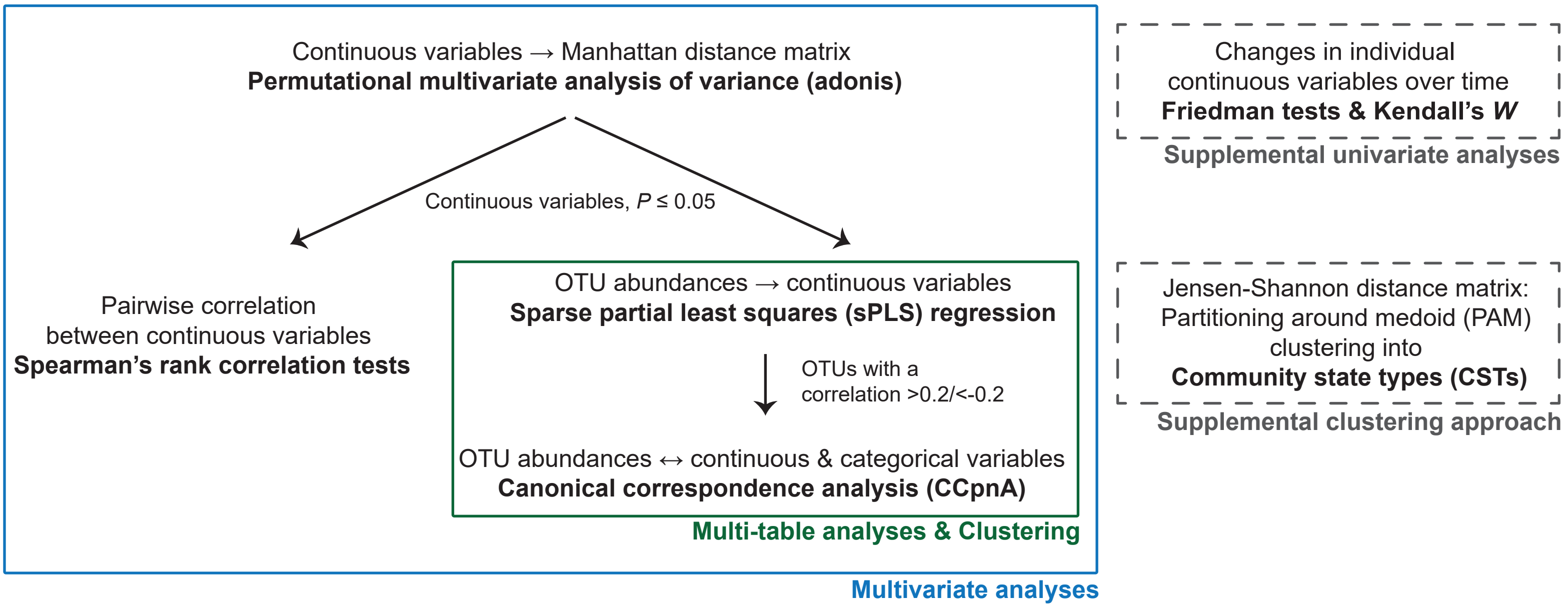

Continuous variables → Manhattan distance matrix
Permutational multivariate analysis of variance (adonis)
Continuous variables, P ≤ 0.05
Pairwise correlation
between continuous variables
Spearman's rank correlation tests
OTU abundances → continuous variables
Sparse partial least squares (sPLS) regression
OTUs with a
correlation >0.2/<-0.2
OTU abundances ↔ continuous & categorical variables
Canonical correspondence analysis (CCpnA)
Multi-table analyses & Clustering
Multivariate analyses
Changes in individual
continuous variables over time
Friedman tests & Kendall's W
Supplemental univariate analyses
Jensen-Shannon distance matrix:
Partitioning around medoid (PAM)
clustering into
Community state types (CSTs)
Supplemental clustering approach
