# Supplementary material for: Specific gut microbiome members are associated with distinct immune markers in pediatric allogeneic hematopoietic stem cell transplantation
Source: Microbiome. 2019 Sep 13;7:131. doi: 10.1186/s40168-019-0745-z (PMC6744702; doi:10.1186/s40168-019-0745-z)
Supplement: Supplementary file 4 — Table S2. Results of Permutational Multivariate Analysis of Variance Using Distance Matrices (adonis). Adonis was employed for model selection to identify relevant immune markers and immune cell types to be included in downstream analyses (See Methods for details). Significant variables (P<0.05) are marked in bold. Abbreviations: hBD2_sim, plasma human beta-defensin 2 levels at time points simultaneous to microbiome characterization; CRP_sim, C-reactive protein levels at time points simultaneous to microbiome characterization; Lymphocyte_count_sim, total lymphocyte counts at time points simultaneous to microbiome characterization; pIL6, plasma interleukin 6 concentration; Citr, plasma citrulline concentration; CD3+, CD3+ T cell counts; CD4+, CD3+CD4+ T cell counts; CD8+, CD3+CD8+ T cell counts; B, total B cell (CD45+CD19+) counts; mat_B, mature B cell (CD45+CD19+CD20+) counts. immat_B, immature B cell (CD45+CD19+CD20-) counts; NK, natural killer cell counts; mean_mono, mean monocyte counts at indicated time point; mean_neutro, mean neutrophil counts at indicated time point; Timepoints: pre, prior to transplantation; w0, on the day of transplantation; w1, w2, w3, w4, w5: one, two, three, four and five weeks after transplantation, respectively; m1, m2, m3, m4, m6: one, two, three, four and six months after transplantation, respectively; 1y, 1 year post-transplantation. (PDF 461 kb) [file 40168_2019_745_MOESM4_ESM.pdf]

Variable	SumOfSqs	F	*P*-value
hBD2_sim	9763	0.4635	0.915
**hbd2_pre**	89958	4.3373	**0.001**
**hbd2_w0**	39432	1.9012	**0.037**
**hbd2_w1**	62425	3.0098	**0.007**
**hbd2_w2**	70010	3.3755	**0.004**
hbd2_w3	32106	1.5480	0.088
hbd2_m2	33806	1.6300	0.104
**CRP_sim**	28707	1.3629	**0.015**
CRP_pre	15714	0.8102	0.631
CRP_w0	22573	1.1639	0.280
**CRP_w1**	51055	2.6324	**0.011**
CRP_w2	34122	1.7593	0.098
CRP_w3	41269	2.1278	0.055
CRP_m1	27106	1.3976	0.169
**CRP_w5**	41965	2.1637	**0.026**
**CRP_w6**	38860	2.0036	**0.029**
CRP_m2	31527	1.6255	0.095
**CRP_m3**	39972	2.0610	**0.034**
CRP_m4	23961	1.2354	0.252
**CRP_m6**	39945	2.0596	**0.024**
pIL6_w1	23180	1.1431	0.311
**Citr_pre**	78105	3.8519	**0.002**
**Citr_w1**	42029	2.0727	**0.021**
Citr_w3	33872	1.6705	0.069
CD3+_m1	28654	1.4500	0.123
CD3+_m2	17891	0.9054	0.501
CD4+_m1	30266	1.5316	0.090
**CD4+_m2**	38132	1.9296	**0.037**
**CD8+_m1**	38615	1.9541	**0.029**
CD8+_m2	21275	1.0766	0.337
B_m1	26588	1.3454	0.166
**B_m2**	41803	2.1154	**0.025**
mat_B_m1	26622	1.3472	0.167
**mat_B_m2**	41697	2.1100	**0.027**
immat_B_m1	28078	1.4208	0.133
**immat_B _m2**	40142	2.0313	**0.029**
**NK_m1**	63891	3.2331	**0.002**
**NK_m2**	44566	2.2552	**0.020**
Lymphocyte_count_sim	21541	1.0227	0.610
Lymphocyte_m2	22196	1.1232	0.324

Lymphocyte_m3	24450	1.2373	0.224
Lymphocyte_m6	26278	1.3298	0.177
Lymphocyte_1y	29658	1.5008	0.114
**mean_mono_pre**	47349	2.3109	**0.013**
**mean_mono_w3**	64242	3.1353	**0.002**
mean_mono_m1	17924	0.8748	0.561
mean_mono_m2	25930	1.2655	0.187
mean_mono_m3	18269	0.8916	0.516
mean_mono_m6	1579	0.7708	0.654
mean_neutro_pre	25198	1.2298	0.225
mean_neutro_w3	31161	1.5208	0.149
mean_neutro_m1	29036	1.4171	0.137
mean_neutro_m2	16656	0.8129	0.611
**mean_neutro_m3**	48466	2.3653	**0.010**
mean_neutro_m6	15960	0.7789	0.653
