# Supplementary material for: Specific gut microbiome members are associated with distinct immune markers in pediatric allogeneic hematopoietic stem cell transplantation
Source: Microbiome. 2019 Sep 13;7:131. doi: 10.1186/s40168-019-0745-z (PMC6744702; doi:10.1186/s40168-019-0745-z)
Supplement: Supplementary file 5 — Table S3. Taxonomy and cluster affiliation of OTUs strongly associated with host-related variables based on sPLS analysis and community state typing (CST). List of the 57 OTUs correlated strongest with variables in the sPLS analysis (>0.2/<-0.2) . SPLS-based clusters were determined by applying the mixOmics cim() function to the sPLS regression model (hierarchical clustering method: complete linkage, distance method: Pearson’s correlation) (see Methods). Four community state types (CSTs) were defined by clustering of fecal samples with similar microbial community compositions by partitioning around medoid (PAM) clustering (see Methods). OTUs were then assigned to the CST-based clusters in which they exhibited the highest average abundance over all samples. The same taxonomic families dominated in sPLS- and CST-based clusters, respectively. Cluster 1 was dominated by Lactobacillaceae. Cluster 2 was characterized mainly by Ruminococcaceae and Lachnospiraceae. Cluster 3 harbored Proteobacteria (P), e. g. Enterobacteriaceae. CS-typing revealed one additional cluster (4), characterized by a high abundance of Enterococcaceae and Staphylococcaceae. OTU numbers refer to the SILVA database (silva_119_rep_set97). Phyla abbreviations: F, Firmicutes; B, Bacteroidetes; A, Actinobacteria; P, Proteobacteria; FU, Fusobacteria. (PDF 505 kb) [file 40168_2019_745_MOESM5_ESM.pdf]

OTU number	Phylum	Order	Family	Genus/Species/Description	Cluster	
					**sPLS**	**CST**
HM534767.1	*F*	*Lactobacillales*	*Lactobacillaceae*	*Lactobacillus* sp. Akpobro1	1	1
AF413523.1	*F*	*Lactobacillales*	*Lactobacillaceae*	*Lactobacillus pantheris*	1	1
KF751750.1	*F*	*Lactobacillales*	*Lactobacillaceae*	*Lactobacillus* sp.	1	1
AB257866.1	*F*	*Lactobacillales*	*Lactobacillaceae*	*Lactobacillus suebicus*	1	1
JX986976.1	*F*	*Lactobacillales*	*Lactobacillaceae*	*Lactobacillus aviarius* subsp. *araffinosus*	1	1
GU451064.1	*F*	*Lactobacillales*	*Lactobacillaceae*	*Lactobacillus* sp. 5-1-2	1	1
EU774037.1	*F*	*Lactobacillales*	*Lactobacillaceae*	*Lactobacillus* sp.	1	1
FN667075.1	*F*	*Lactobacillales*	*Lactobacillaceae*	*Lactobacillus* sp.	1	1
EU461951.1	*F*	*Lactobacillales*	*Streptococcaceae*	*Streptococcus* sp.	1	1
EU460769.1	*F*	*Lactobacillales*	*Lactobacillaceae*	*Lactobacillus* sp.	1	1
KC836559.1	*F*	*Lactobacillales*	*Streptococcaceae*	*Streptococcus salivarius* subsp. *thermophilus*	1	1
FJ749794.1	*F*	*Lactobacillales*	*Lactobacillaceae*	*Lactobacillus fermentum*	1	1
KF029502.1	*F*	*Lactobacillales*	*Lactobacillaceae*	*Lactobacillus casei*	1	1
AUFL01000034	*B*	*Bacteroidales*	*Porphyromonadaceae*	*Dysgonomonas capnocytophagoides* DSM 22835	1	1
DQ801646.1	*F*	*Clostridiales*	*Lachnospiraceae*	uncultured bacterium	2	2
EU246837.1	*F*	*Bacillales*	*Staphylococcaceae*	*Staphylococcus* sp. *Cobs2Tis23*	2	4
DQ804549.1	*F*	*Clostridiales*	*Ruminococcaceae*	*Faecalibacterium* sp.	2	2
FJ366680.1	*A*	*Bifidobacteriales*	*Bifidobacteriaceae*	*Bifidobacterium* sp.	2	2

HQ749687.1	*F*	*Clostridiales*	*Ruminococcaceae*	uncultured bacterium	2	2
FJ753793.1	*F*	*Clostridiales*	*Peptostreptococcaceae*	swine fecal bacterium RF1A-Xyl2	2	2
DQ800353	*F*	*Clostridiales*	*Lachnospiraceae*	*Blautia* sp.	2	2
EU887971.1	*F*	*Clostridiales*	*Ruminococcaceae*	uncultured Clostridia bacterium	2	2
JQ608258.1	*F*	*Erysipelotrichales*	*Erysipelotrichaceae*	bacterium NLAE-zl-C558	2	2
AY850535.1		*Bifidobacteriales*	*Bifidobacteriaceae*	*Bifidobacterium* sp.	2	2
DQ326608.1	*F*	*Clostridiales*	*Ruminococcaceae*	uncultured bacterium	2	2
AJ272200.1	*F*	*Lactobacillales*	*Enterococcaceae*	*Enterococcus hirae*	2	4
EU508251.1	*F*	*Erysipelotrichales*	*Erysipelotrichaceae*	uncultured bacterium	2	2
DQ807523.1	*F*	*Clostridiales*	*Lachnospiraceae*	uncultured bacterium	2	2
CP001726	*A*	*Coriobacteriales*	*Coriobacteriaceae*	*Eggerthella lenta* DSM 2243	2	2
HQ794871.1	*F*	*Erysipelotrichales*	*Erysipelotrichaceae*	*uncultured organism*	2	2
JF193283.1	*F*	*Clostridiales*	*Family XI*	*Finegoldia* sp.	2	2
DQ802363.1	*F*	*Clostridiales*	*Lachnospiraceae*	*Blautia* sp.	2	2
GQ175428.1	*F*	*Clostridiales*	*Ruminococcaceae*	uncultured bacterium	2	2
EU778851.1	*F*	*Clostridiales*	*Ruminococcaceae*	*Subdoligranulum* sp.	2	2
GQ133038.1	*F*	*Lactobacillales*	*Enterococcaceae*	*Enterococcus* sp.	2	4
JQ448431.1	*F*	*Lactobacillales*	*Streptococcaceae*	*Streptococcus* sp.	3	1
JQ460192.1	*B*	*Bacteroidales*	*Prevotellaceae*	*Prevotella* sp.	3	3
M58833.1	*F*	*Lactobacillales*	*Lactobacillaceae*	*Pediococcus acidilactici*	3	1

HG799951.1	*F*	*Bacillales*	*Staphylococcaceae*	*Staphylococcus epidermidis*	3	3
JF235878.1	*A*	*Micrococcales*	*Microbacteriaceae*	*Naasia* sp.	3	3
JQ680457.1	*A*	*Corynebacteriales*	*Corynebacteriaceae*	*Corynebacterium* sp. DYS15	3	3
GQ379595.1	*P*	*Xanthomonadales*	*Xanthomonadaceae*	*Stenotrophomonas* sp.	3	4
AB787271.1	*B*	*Bacteroidales*	*Bacteroidaceae*	*Bacteroides* sp. UasXn-3	3	3
AOTI010230528.6	*B*	*Flavobacteriales*	*Flavobacteriaceae*	*Chryseobacterium Triticum urartu*	3	3
JF109069.1	*F*	*Bacillales*	*Staphylococcaceae*	*Staphylococcus* sp.	3	3
JX464212.1	*F*	*Bacillales*	*Paenibacillaceae*	*Paenibacillus* sp. *BJC15-C21*	3	3
HQ778412.1	*B*	*Bacteroidales*	*Bacteroidaceae*	*Bacteroides* sp.	3	2
FJ976590.1	*P*	*Enterobacteriales*	*Enterobacteriaceae*	*Enterobacter* sp. *LCR81*	3	3
KF178309.1	*F*	*Lactobacillales*	*Lactobacillaceae*	*Pediococcus pentosaceus*	3	3
HQ703879.1	*F*	*Lactobacillales*	*Carnobacteriaceae*	*Alkalibacterium* sp.	3	3
FJ950694.1	*P*	*Enterobacteriales*	*Enterobacteriaceae*	*Escherichia coli*	3	3
JF164165.1	*F*	*Bacillales*	*Staphylococcaceae*	*Staphylococcus* sp.	3	3
KC463799.1	*P*	*Enterobacteriales*	*Enterobacteriaceae*	*Klebsiella* sp.	3	3
JF146381.1	*F*	*Lactobacillales*	*Streptococcaceae*	*Streptococcus* sp.	3	3
AM900778.1	*F*	*Bacillales*	*Paenibacillaceae*	*Paenibacillus* sp. PA215	3	3
CBVB010000006	*B*	*Bacteroidales*	*Bacteroidaceae*	bacterium MS4	3	3
AB013258.1	*P*	*Neisseriales*	*Neisseriaceae*	uncultured beta proteobacterium	3	3
