# Supplementary material for: Specific gut microbiome members are associated with distinct immune markers in pediatric allogeneic hematopoietic stem cell transplantation
Source: Microbiome. 2019 Sep 13;7:131. doi: 10.1186/s40168-019-0745-z (PMC6744702; doi:10.1186/s40168-019-0745-z)
Supplement: Supplementary file 9 — Figure S6. Longitudinal profiles of microbial community composition and immune markers in patients with aGvHD who survived. In three representative patients with moderate to severe aGvHD who survived, high abundance of Lactobacillaceae was observed already before aGvHD onset. None of the depicted patients had a bacterial infection recorded during the monitored period. InvSimpson, inverse Simpson diversity index; hBD2, human beta-defensin 2. (PDF 358 kb) [file 40168_2019_745_MOESM9_ESM.pdf]

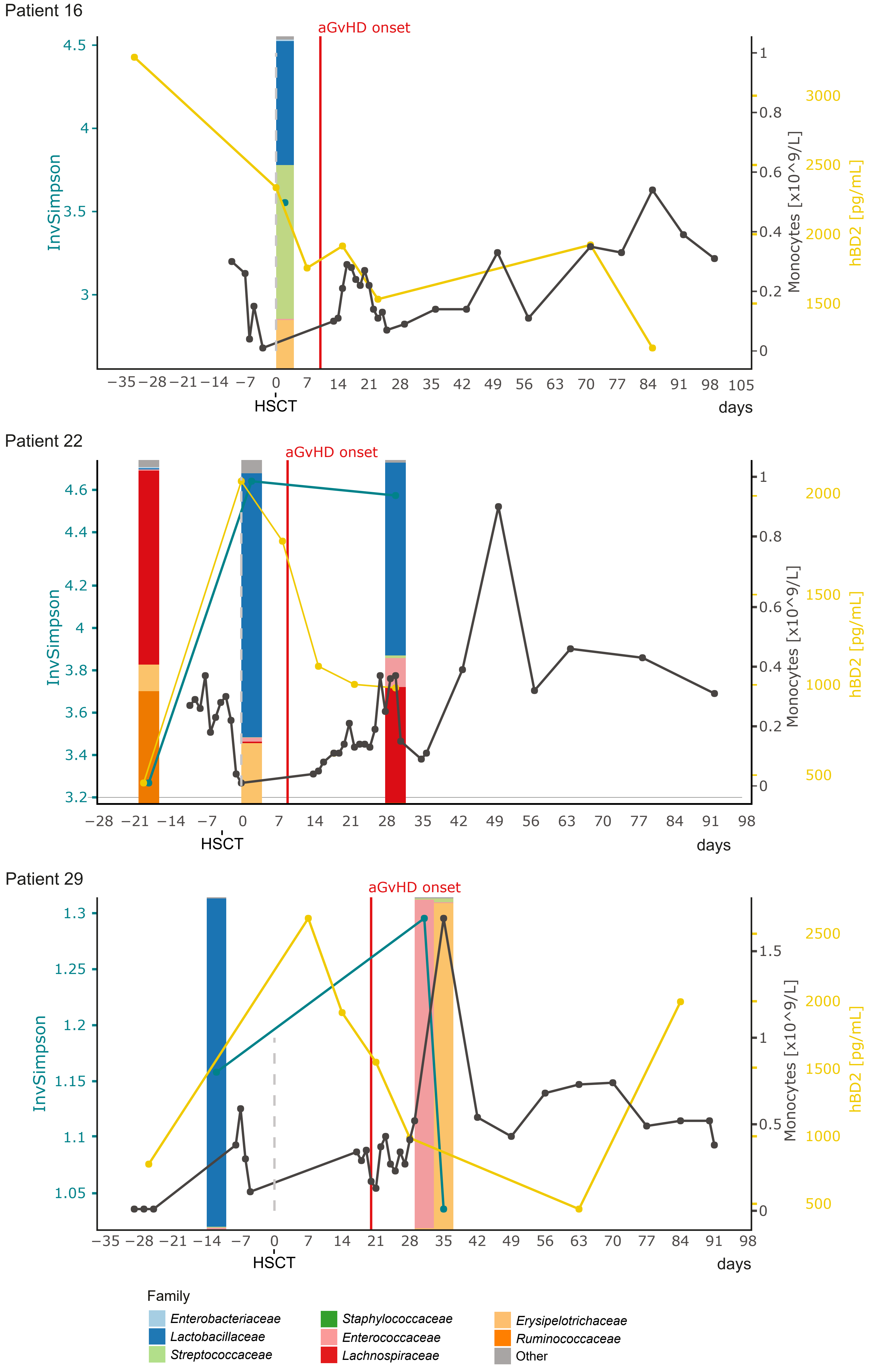
Patient 16
aGvHD onset
InvSimpson
4.5
4
3.5
3
−35
−28
−21
−14
−7
0
7
14
21
28
35
42
49
56
63
70
77
84
91
98
105
HSCT
days
1
0.8
0.6
0.4
0.2
0
Monocytes [x10^9/L]
3000
2500
2000
1500
hBD2 [pg/mL]
Patient 22
aGvHD onset
4.6
4.4
4.2
4
3.8
3.6
3.4
3.2
−28
−21
−14
−7
0
7
14
21
28
35
42
49
56
63
70
77
84
91
98
HSCT
days
1
0.8
0.6
0.4
0.2
0
Monocytes [x10^9/L]
2000
1500
1000
500
hBD2 [pg/mL]
Patient 29
aGvHD onset
1.3
1.25
1.2
1.15
1.1
1.05
InvSimpson
−35
−28
−21
−14
−7
0
7
14
21
28
35
42
49
56
63
70
77
84
91
98
HSCT
days
1.5
1
0.5
0
Monocytes [x10^9/L]
2500
2000
1500
1000
500
hBD2 [pg/mL]
Family
Enterobacteriaceae
Lactobacillaceae
Streptococcaceae
Staphylococcaceae
Enterococcaceae
Lachnospiraceae
Erysipelotrichaceae
Ruminococcaceae
Other
