# Supplementary material for: Specific gut microbiome members are associated with distinct immune markers in pediatric allogeneic hematopoietic stem cell transplantation
Source: Microbiome. 2019 Sep 13;7:131. doi: 10.1186/s40168-019-0745-z (PMC6744702; doi:10.1186/s40168-019-0745-z)
Supplement: Supplementary file 10 — Supplementary Discussion. Discussion concerning survival following high Lactobacillaceae abundances prior to the onset of aGvHD, and associations of adverse outcomes with Enterococcus compared with previous studies. (PDF 732 kb) [file 40168_2019_745_MOESM10_ESM.pdf]

Our results suggest that a high *Lactobacillaceae* abundance prior to the onset of aGvHD may point to a preventive effect, as these patients survived. A human clinical trial of *Lactobacillus rhamnosus* GG prebiotic gavage to HSCT patients at time of engraftment demonstrated no protection against GvHD [1]. This could mean that *Lactobacillaceae* may not play a key role in aGvHD development, at least not the particular *Lactobacillus rhamnosus* strain under the conditions used in this group of patients. However, the administered probiotic did not alter the abundance of *Lactobacillus* spp. in the patients' guts [1], suggesting that the strain was not able to establish and proliferate in the host environment in this situation. An intrinsic increase of *Lactobacillaceae* prior to aGvHD onset, as observed here, therefore might still play a role in reducing aGvHD. A recent study related to the use of a probiotic given to infants to prevent sepsis suggested that the time point of application of a specific *Lactobacillus* sp. strain as a synbiotic played a critical role in positive clinical outcomes [2]. Furthermore, a study on gut microbial immunomodulation emphasized the importance of characterizing bacteria at the strain-level, because individual strains can have different modulatory effects on the immune system [3]. Therefore, it would be of great interest to determine the identity and predicted function of the specific *Lactobacillus* spp. strains in our patients, and in particular, in those who exhibited an early high abundance of *Lactobacillus* spp., as compared with those who experienced an expansion of *Lactobacillus* spp. after aGvHD and who later died.

Interestingly, *Enterococcus* was not among the most relevant taxa identified by our multivariate analyses. Intestinal domination of *Enterococcus* spp. was not clearly associated with adverse outcomes in our subgroup of 30 patients, in contrast to previous findings [4–6]. It should be noted that these previous observations were made in adult allo-HSCT patients and were dependent on

the type and amount of antimicrobial treatment. In addition, to elucidate this discrepancy further, we are currently characterizing *Enterococcus* isolates from fecal samples of our patient group, to gain insight into bacterial strain-level differences.

Supplementary references

1. Gorshein E, Wei C, Ambrosy S, Budney S, Vivas J, Shenkerman A, et al. *Lactobacillus rhamnosus GG* probiotic enteric regimen does not appreciably alter the gut microbiome or provide protection against GVHD after allogeneic hematopoietic stem cell transplantation. Clin Transplant. 2017;31:e12947. doi:10.1111/ctr.12947.

2. Panigrahi P, Parida S, Nanda NC, Satpathy R, Pradhan L, Chandel DiS, et al. A randomized synbiotic trial to prevent sepsis among infants in rural India. Nature. 2017;548:407–12. doi:10.1038/nature23480.

3. Geva-Zatorsky N, Sefik E, Kua L, Pasman L, Tan TG, Ortiz-Lopez A, et al. Mining the Human Gut Microbiota for Immunomodulatory Organisms. Cell. 2017;168:928–943.e11. doi:10.1016/j.cell.2017.01.022.

4. Shono Y, van den Brink MRM. Gut microbiota injury in allogeneic haematopoietic stem cell transplantation. Nat Rev Cancer. 2018. doi:10.1038/nrc.2018.10.

5. Taur Y, Xavier JB, Lipuma L, Ubeda C, Goldberg J, Gobourne a., et al. Intestinal Domination and the Risk of Bacteremia in Patients Undergoing Allogeneic Hematopoietic Stem Cell Transplantation. Clin Infect Dis. 2012;55:905–14. doi:10.1093/cid/cis580.

6. Holler E, Butzhammer P, Schmid K, Hundsrucker C, Koestler J, Peter K, et al. Metagenomic Analysis of the Stool Microbiome in Patients Receiving Allogeneic Stem Cell Transplantation: Loss of Diversity Is Associated with Use of Systemic Antibiotics and More Pronounced in Gastrointestinal Graft-versus-Host Disease. Biol Blood Marrow Transplant. 2014;20:640–5. doi:10.1016/j.bbmt.2014.01.030.
