# Supplementary material for: Specific gut microbiome members are associated with distinct immune markers in pediatric allogeneic hematopoietic stem cell transplantation
Source: Microbiome. 2019 Sep 13;7:131. doi: 10.1186/s40168-019-0745-z (PMC6744702; doi:10.1186/s40168-019-0745-z)
Supplement: Supplementary file 11 — R data analysis report 1. Data preparation, filtering and transformation. (HTML 2337 kb) [file 40168_2019_745_MOESM11_ESM.html]

Data preparation, filtering and transformation


# Data preparation, filtering and transformation

#### *Anna Caecilia Masche*

#### *June 2018*

This is the 1st script used for data analysis and figure generation for the the manuscript by Masche et al. titled “Specific gut microbiome members are associated with distinct immune markers in allogeneic hematopoietic stem cell transplantation”.

This script and associated data are provided by Anna Cäcilia Masche, Susan Holmes, and Sünje Johanna Pamp.

These data and the associated script are licensed under the Creative Commons Attribution-ShareAlike 4.0 International License. To view a copy of this license, visit http://creativecommons.org/licenses/by-sa/4.0/ or send a letter to Creative Commons, PO Box 1866, Mountain View, CA 94042, USA.

Under the condition that appropriate credit is provided, you are free to: 1) Share, copy and redistribute the material in any medium or format 2) Adapt, remix, transform, and build upon the material for any purpose, even commercially.

To see the full license associated with attribution of this work, see the CC-By-CA license, see http://creativecommons.org/licenses/by-sa/4.0/.

The local filename is: Script1\_data\_prep.Rmd.

## **Install required packages from CRAN and Bioconductor:**

```
#pkgs_needed = c("devtools", "cluster","RColorBrewer", "gridExtra", "plyr", "dplyr", "ggbiplot", "ggord", , "DESeq2", "genefilter", "vegan", "mixOmics", "SummarizedExperiment","phyloseq")

#letsinstall = setdiff(pkgs_needed, installed.packages(dependencies = TRUE)) 

#if (length(letsinstall) > 0) {
#  source("http://bioconductor.org/biocLite.R")
#  biocLite(letsinstall)
#}
```

## **Load required packages:**

```
library("ggplot2")
library("ggbiplot")
```

```
## Loading required package: plyr
```

```
## Loading required package: scales
```

```
## Warning: package 'scales' was built under R version 3.4.3
```

```
## Loading required package: grid
```

```
library("ggord")
library("GenomeInfoDb")
```

```
## Loading required package: BiocGenerics
```

```
## Loading required package: parallel
```

```
## 
## Attaching package: 'BiocGenerics'
```

```
## The following objects are masked from 'package:parallel':
## 
##     clusterApply, clusterApplyLB, clusterCall, clusterEvalQ,
##     clusterExport, clusterMap, parApply, parCapply, parLapply,
##     parLapplyLB, parRapply, parSapply, parSapplyLB
```

```
## The following objects are masked from 'package:stats':
## 
##     IQR, mad, sd, var, xtabs
```

```
## The following objects are masked from 'package:base':
## 
##     anyDuplicated, append, as.data.frame, cbind, colMeans,
##     colnames, colSums, do.call, duplicated, eval, evalq, Filter,
##     Find, get, grep, grepl, intersect, is.unsorted, lapply,
##     lengths, Map, mapply, match, mget, order, paste, pmax,
##     pmax.int, pmin, pmin.int, Position, rank, rbind, Reduce,
##     rowMeans, rownames, rowSums, sapply, setdiff, sort, table,
##     tapply, union, unique, unsplit, which, which.max, which.min
```

```
## Loading required package: S4Vectors
```

```
## Loading required package: stats4
```

```
## 
## Attaching package: 'S4Vectors'
```

```
## The following object is masked from 'package:plyr':
## 
##     rename
```

```
## The following object is masked from 'package:base':
## 
##     expand.grid
```

```
## Loading required package: IRanges
```

```
## 
## Attaching package: 'IRanges'
```

```
## The following object is masked from 'package:plyr':
## 
##     desc
```

```
library("phyloseq")
```

```
## 
## Attaching package: 'phyloseq'
```

```
## The following object is masked from 'package:IRanges':
## 
##     distance
```

```
library("gridExtra")
```

```
## 
## Attaching package: 'gridExtra'
```

```
## The following object is masked from 'package:BiocGenerics':
## 
##     combine
```

```
library("plyr")
library("dplyr")
```

```
## Warning: package 'dplyr' was built under R version 3.4.1
```

```
## 
## Attaching package: 'dplyr'
```

```
## The following object is masked from 'package:gridExtra':
## 
##     combine
```

```
## The following object is masked from 'package:GenomeInfoDb':
## 
##     intersect
```

```
## The following objects are masked from 'package:IRanges':
## 
##     collapse, desc, intersect, setdiff, slice, union
```

```
## The following objects are masked from 'package:S4Vectors':
## 
##     first, intersect, rename, setdiff, setequal, union
```

```
## The following objects are masked from 'package:BiocGenerics':
## 
##     combine, intersect, setdiff, union
```

```
## The following objects are masked from 'package:plyr':
## 
##     arrange, count, desc, failwith, id, mutate, rename, summarise,
##     summarize
```

```
## The following objects are masked from 'package:stats':
## 
##     filter, lag
```

```
## The following objects are masked from 'package:base':
## 
##     intersect, setdiff, setequal, union
```

```
library("DESeq2")
```

```
## Loading required package: GenomicRanges
```

```
## Loading required package: SummarizedExperiment
```

```
## Loading required package: Biobase
```

```
## Welcome to Bioconductor
## 
##     Vignettes contain introductory material; view with
##     'browseVignettes()'. To cite Bioconductor, see
##     'citation("Biobase")', and for packages 'citation("pkgname")'.
```

```
## 
## Attaching package: 'Biobase'
```

```
## The following object is masked from 'package:phyloseq':
## 
##     sampleNames
```

```
## Loading required package: DelayedArray
```

```
## Loading required package: matrixStats
```

```
## 
## Attaching package: 'matrixStats'
```

```
## The following objects are masked from 'package:Biobase':
## 
##     anyMissing, rowMedians
```

```
## The following object is masked from 'package:dplyr':
## 
##     count
```

```
## The following object is masked from 'package:plyr':
## 
##     count
```

```
## 
## Attaching package: 'DelayedArray'
```

```
## The following objects are masked from 'package:matrixStats':
## 
##     colMaxs, colMins, colRanges, rowMaxs, rowMins, rowRanges
```

```
## The following object is masked from 'package:base':
## 
##     apply
```

```
library("genefilter")
```

```
## 
## Attaching package: 'genefilter'
```

```
## The following objects are masked from 'package:matrixStats':
## 
##     rowSds, rowVars
```

```
library("vegan")
```

```
## Warning: package 'vegan' was built under R version 3.4.1
```

```
## Loading required package: permute
```

```
## Warning: package 'permute' was built under R version 3.4.1
```

```
## Loading required package: lattice
```

```
## This is vegan 2.4-3
```

```
library("mixOmics")
```

```
## Warning: package 'mixOmics' was built under R version 3.4.1
```

```
## Loading required package: MASS
```

```
## 
## Attaching package: 'MASS'
```

```
## The following object is masked from 'package:genefilter':
## 
##     area
```

```
## The following object is masked from 'package:dplyr':
## 
##     select
```

```
## 
## Loaded mixOmics 6.1.3
## 
## Visit http://www.mixOmics.org for more details about our methods.
## Any bug reports or comments? Notify us at mixomics at math.univ-toulouse.fr or https://bitbucket.org/klecao/package-mixomics/issues
## 
## Thank you for using mixOmics!
```

```
library("SummarizedExperiment")
```

## **Import the data and combine into a phyloseq object:**

### **Import the OTU count table:**

#### The file OTU\_table.txt is provided.

```
otu_mat <- read.table("M:/Documents/Publications/Masche_R_Scripts_and_Data/Data/OTU_table.txt", sep = "", header = TRUE, row.names = 1)

otu_mat1 <- as.matrix(otu_mat)

otu_mat2 <- otu_table(otu_mat1, taxa_are_rows = TRUE)
```

### **Import the taxonomy table:**

#### The file Taxonomy\_table.txt is provided.

```
tax_mat <- read.table("M:/Documents/Publications/Masche_R_Scripts_and_Data/Data/Taxonomy_table.txt", sep = ",", header = TRUE, row.names = 1)

tax_mat1 <- as.matrix(tax_mat)

tax_mat2 <- tax_table(tax_mat1)
```

### **Combine OTU count table and taxonomy table into a phyloseq object:**

```
phy_obj = phyloseq(otu_mat2, tax_mat2)
phy_obj
```

```
## phyloseq-class experiment-level object
## otu_table()   OTU Table:         [ 756 taxa and 98 samples ]
## tax_table()   Taxonomy Table:    [ 756 taxa by 7 taxonomic ranks ]
```

### **Import the sample\_data file and merge it with the phyloseq object:**

#### The file Data\_matrix\_30\_patients.txt is provided.

```
map_file <- read.table("M:/Documents/Publications/Masche_R_Scripts_and_Data/Data/Data_matrix_30_patients.txt", sep = "", header = TRUE, row.names = 1)

map_file1 <- sample_data(map_file)

phy_obj1 <- merge_phyloseq(phy_obj, map_file1)
```

## **Create a “core” OTU set:**

#### Keep only those OTUs with at least 5 reads in at least 2 samples by using the `kOverA()` function in package `genefilter`:

```
function1 <- genefilter::kOverA(2,4) #selects OTUs that have a read count of at least 5 in at least 2 samples

logi <- filter_taxa(phy_obj1, function1, prune=FALSE) #which OTUs passed the filtering? (TRUE/FALSE)

phy_obj1_core <- filter_taxa(phy_obj1, function1, prune=TRUE) #new filtered phyloseq object  

phy_obj1_core #How many OTUs passed the filtering? --> 256 of 756
```

```
## phyloseq-class experiment-level object
## otu_table()   OTU Table:         [ 256 taxa and 98 samples ]
## sample_data() Sample Data:       [ 98 samples by 417 sample variables ]
## tax_table()   Taxonomy Table:    [ 256 taxa by 7 taxonomic ranks ]
```

## **Create a “cleaned/decontaminated” OTU set (from “core” OTUs):**

#### Identify potential contaminants in the negative control and exclude these from the data set.

```
NC <- grep("negative_control", sample_names(phy_obj1_core), value=TRUE) #grep extraction control
NC_subset <- prune_samples(sample_names(phy_obj1_core) %in% NC, phy_obj1_core)#subset extraction control
```

### **Look at the abundances of OTUs (>1) of the negative control in all the samples:**

```
potential_contaminants_all <- prune_taxa(taxa_sums(NC_subset) >0, phy_obj1_core)

potential_contaminants_all_psm <- psmelt(potential_contaminants_all)
```

### **Plot histograms of abundance in all samples for each OTU occurring in the negative control:**

#### Add a vertical line for the abundance in the negative control

```
test <- potential_contaminants_all_psm[potential_contaminants_all_psm$Sample =="negative_control", ]

contam_abund <- ggplot(potential_contaminants_all_psm[potential_contaminants_all_psm$Sample!= "negative_control" , ], aes(x=Abundance)) + geom_histogram(binwidth = 1) + facet_wrap(~OTU + Family + Species,  scales = 'free_x', ncol = 1) + geom_vline(aes(xintercept = Abundance), test, col = "red", size = 1) + theme(plot.title = element_text(size = 14, face = "bold"))

contam_abund
```

##### **We will exclude the following OTUs that are more abundant in the negative control than in all of the samples as potential contaminants:**

AF526940.1.1489  
EU723168.1.1319

##### **We will exclude the following OTUs that are more abundant in the negative control than in most of the samples as potential contaminants:**

EU294137.1.1446  
EU464668.1.1415  
EU489741.1.1434  
EU705372.1.1307  
FJ595883.1.1379  
FQ659262.1.1358  
GQ140331.1.1495  
HM237107.1.1268  
HQ113223.1.1408  
JF154537.1.1353  
JF706644.1.1410  
JN700217.1.1287  
KC541230.1.1480  
KC814649.1.1276  
KF192057.1.1398

##### **OTUs not to be excluded as contaminants because they are similarly abundant in the negative control as in most of the samples, so it cannot be determined if the OTU originates from the negative control or a sample (overspill to negative control):**

DQ264411.1.1519  
FJ950694.1.1472  
FJ976590.1.1390  
GQ133038.1.1416  
GQ156251.1.1363  
KF029502.1.1525

### **Now exclude the 17 OTUs we identified as potential contaminants before in the plot:**

```
# Define the taxa to be excluded:
badTaxa = c("AF526940.1.1489", "EU723168.1.1319", "EU294137.1.1446", "EU464668.1.1415", "EU489741.1.1434", "EU705372.1.1307", "FJ595883.1.1379", "FQ659262.1.1358", "GQ140331.1.1495", "HM237107.1.1268", "HQ113223.1.1408", "JF154537.1.1353", "JF706644.1.1410", "JN700217.1.1287", "KC541230.1.1480", "KC814649.1.1276", "KF192057.1.1398")

length(badTaxa)
```

```
## [1] 17
```

```
allTaxa <- taxa_names(phy_obj1_core)

keepTaxa <- allTaxa[!(allTaxa %in% badTaxa)]

phy_obj1_core_cleaned <- prune_taxa(keepTaxa, phy_obj1_core)
# new phyloseq object with just the taxa we kept. 

phy_obj1_core_cleaned  #239 taxa left
```

```
## phyloseq-class experiment-level object
## otu_table()   OTU Table:         [ 239 taxa and 98 samples ]
## sample_data() Sample Data:       [ 98 samples by 417 sample variables ]
## tax_table()   Taxonomy Table:    [ 239 taxa by 7 taxonomic ranks ]
```

### **Write the phyloseq object with core taxa and without potential contaminants to a file to be used in the follow-up scripts:**

#### The file phy\_obj1\_core\_cleaned.Rdata is provided.

```
#saveRDS(phy_obj1_core_cleaned, file = "M:/Documents/Publications/Masche_R_Scripts_and_Data/Data/phy_obj1_core_cleaned.Rdata")
```

## **Explore sequencing depth before any alterations of the count table:**

#### Add a vertical line at read count = 2000

### **Initial sequencing depth:**

```
sdt = data.table::data.table(as(sample_data(phy_obj1), "data.frame"),
                 TotalReads = sample_sums(phy_obj1), keep.rownames = TRUE)
data.table::setnames(sdt, "rn", "SampleID")
pSeqDepth = ggplot(sdt, aes(TotalReads)) + geom_histogram(binwidth = 500) + geom_vline(xintercept = 2000, color= "red") + annotate("text", label = nrow(sdt[sdt$TotalReads<2000]), x = 700, y = 1, size = 4, colour = "black")  + ggtitle("Sequencing Depth raw counts") + theme(plot.title = element_text(size = 14, face = "bold"))
```

### **Sequencing depth after selecting a core microbiome:**

```
sdt_core = data.table::data.table(as(sample_data(phy_obj1_core), "data.frame"),
                 TotalReads = sample_sums(phy_obj1_core), keep.rownames = TRUE)
data.table::setnames(sdt_core, "rn", "SampleID")
pSeqDepth_core = ggplot(sdt_core, aes(TotalReads)) + geom_histogram(binwidth = 500) +  geom_vline(xintercept = 2000, color= "red") + annotate("text", label = nrow(sdt_core[sdt_core$TotalReads<2000]), x = 700, y = 1, size = 4, colour = "black") + ggtitle("Sequencing Depth core counts") + theme(plot.title = element_text(size = 14, face = "bold"))
```

### **Sequencing depth after removing potential contaminants:**

```
sdt_core_cleaned <- data.table::data.table(as(sample_data(phy_obj1_core_cleaned), "data.frame"),
                 TotalReads = sample_sums(phy_obj1_core_cleaned), keep.rownames = TRUE)
data.table::setnames(sdt_core_cleaned, "rn", "SampleID")
pSeqDepth_core_cleaned = ggplot(sdt_core_cleaned, aes(TotalReads)) + geom_histogram(binwidth = 500)  + geom_vline(xintercept = 2000, color= "red") + annotate("text", label = nrow(sdt_core_cleaned[sdt_core_cleaned$TotalReads<2000]), x = 700, y = 1, size = 4, colour = "black") + ggtitle("Sequencing Depth core and cleaned counts") + theme(plot.title = element_text(size = 14, face = "bold"))
```

```
gridExtra::grid.arrange(pSeqDepth, pSeqDepth_core, pSeqDepth_core_cleaned)
```

## **Conversion from `phyloseq` object to `DESeq2` object:**

#### Now we will convert the phyloseq object to a `DESeq2` DESeqDataSet-class object to transform the OTU counts by `DESeq`’s `varianceStabilizingTransformation()` function for downstream analysis.

```
my_dds_all <- phyloseq::phyloseq_to_deseq2(phy_obj1_core_cleaned, ~timepoint)
```

```
## converting counts to integer mode
```

#### The geometric means cannot be calculated if there are 0s present. Therefore, we first calculate size factors separately using a zero-tolerant variant of the geometric mean.

### **Calculate geometric means prior to estimating size factors:**

```
gm_mean = function(x, na.rm=TRUE){
  exp(sum(log(x[x > 0]), na.rm=na.rm) / length(x))
}

geoMeans_all <- apply(counts(my_dds_all), 1, gm_mean)
my_dds_all2 <- estimateSizeFactors(my_dds_all, geoMeans = geoMeans_all)
```

### **Variance stabilizing transformation of the OTU count data (for downstream analyses):**

#### I will use variance stabilization (I also considered and tried rlog transformation, but got the warning that my data was too sparse).

#### Transformation will be blinded to the experimental design to allow comparing samples unbiased by prior information.

```
vsd_unbiased <- DESeq2::varianceStabilizingTransformation(my_dds_all2, blind = TRUE, fitType = "local")

#make a phyloseq object with the transformed OTU table 
phy_obj1_core_cleaned_vsd <- phy_obj1_core_cleaned

otu_table(phy_obj1_core_cleaned_vsd) <- phyloseq::otu_table(SummarizedExperiment::assay(vsd_unbiased), taxa_are_rows = TRUE)
```

## **Calculate alpha diversity indices based on the untransformed data (here referring to “core” and “cleaned”, but not variance stabilized data)**

#### I ran `estimate_richness()` on untransformed data (actually - because some diversity measures depend on singletons and doubletons - it should even be done before the “core” microbiome step, but on the other hand these single- and doubletons create a lot of noise that distort the diversity results).

```
alpha_div_indices_raw <- estimate_richness(phy_obj1_core_cleaned, split = TRUE, measures = c("Observed","InvSimpson"))
```

### **Add the resulting alpha diversity results to the sample data in the latest phyloseq object (`phy_obj1_core_cleaned_vsd`):**

```
alpha_div_indices_raw_sampledata_format <- sample_data(alpha_div_indices_raw)

phy_obj1_core_cleaned_vsd1 <- merge_phyloseq(phy_obj1_core_cleaned_vsd, alpha_div_indices_raw_sampledata_format)
```

### **Plot richness (raw) by patient:**

```
rich1 <- phyloseq::plot_richness(phy_obj1_core_cleaned, x = "Patient_ID", color = NULL, shape = NULL,
  title = NULL, scales = "free_y", nrow = 1, shsi = NULL,
  measures = "InvSimpson", sortby = NULL)
print(rich1)
```

### **Plot richness (raw) by patient and time point:**

```
rich2 <- phyloseq::plot_richness(phy_obj1_core_cleaned, x="timepoint", color = "Patient_ID", measures = c("InvSimpson"), title = "Alpha Diversity with raw counts") + geom_point(size=3, aes(color = Patient_ID))+ geom_line(size=1, aes(x = timepoint, group = Patient_ID, color = Patient_ID))

print(rich2)
```

For comparison we also calculated alpha diversity by different indices based on rarefied data (rarefying was performed by the `rarefy_even_depth()` function in `phyloseq` using a minimum sample size of 2000). Subsequently we assessed if the inverse Simpson index differed when using raw compared to rarefied data depending on the total read count. There seemed to be no difference (code and graphs not included here). Therefore we continued with using the invSimpson index based on raw data.

### **Write the updated phyloseq object to a file to be used in the follow-up scripts:**

#### The file phy\_obj1\_core\_cleaned\_vsd1.Rdata is provided.

```
#saveRDS(phy_obj1_core_cleaned_vsd1, file = "M:/Documents/Publications/Masche_R_Scripts_and_Data/Data/phy_obj1_core_cleaned_vsd1.Rdata")
```
