# Supplementary material for: Specific gut microbiome members are associated with distinct immune markers in pediatric allogeneic hematopoietic stem cell transplantation
Source: Microbiome. 2019 Sep 13;7:131. doi: 10.1186/s40168-019-0745-z (PMC6744702; doi:10.1186/s40168-019-0745-z)
Supplement: Supplementary file 12 — R data analysis report 2. Bacterial alpha-diversity over time and rank abundance curve for the gut microbiome of HSCT patients. (HTML 1461 kb) [file 40168_2019_745_MOESM12_ESM.html]

Bacterial alpha-diversity and relative abundance over time for the gut microbiome of HSCT patients


# Bacterial alpha-diversity and relative abundance over time for the gut microbiome of HSCT patients

#### Anna Caecilia Masche

#### June 2019

This is the 2nd script used for data analysis and figure generation for the the manuscript by Masche et al. titled “Specific gut microbiome members are associated with distinct immune markers in allogeneic hematopoietic stem cell transplantation”.

This script and associated data are provided by Anna Caecilia Masche, Susan Holmes, and Suenje Johanna Pamp.

These data and the associated script are licensed under the Creative Commons Attribution-ShareAlike 4.0 International License. To view a copy of this license, visit http://creativecommons.org/licenses/by-sa/4.0/ or send a letter to Creative Commons, PO Box 1866, Mountain View, CA 94042, USA.

Under the condition that appropriate credit is provided, you are free to: 1) Share, copy and redistribute the material in any medium or format 2) Adapt, remix, transform, and build upon the material for any purpose, even commercially.

To see the full license associated with attribution of this work, see the CC-By-CA license, see http://creativecommons.org/licenses/by-sa/4.0/.

The local filename is: Script2\_diversity.Rmd.

## **Load required packages:**

```
library("phyloseq")
```

```
## Registered S3 methods overwritten by 'ggplot2':
##   method         from 
##   [.quosures     rlang
##   c.quosures     rlang
##   print.quosures rlang
```

```
library("dplyr")
```

```
## 
## Attaching package: 'dplyr'
```

```
## The following objects are masked from 'package:stats':
## 
##     filter, lag
```

```
## The following objects are masked from 'package:base':
## 
##     intersect, setdiff, setequal, union
```

```
library("ggplot2")
library("gridExtra")
```

```
## 
## Attaching package: 'gridExtra'
```

```
## The following object is masked from 'package:dplyr':
## 
##     combine
```

```
library("reshape2")
#install.packages("PMCMR")
library("PMCMR")
```

```
## PMCMR is superseded by PMCMRplus and will be no longer maintained. You may wish to install PMCMRplus instead.
```

## **Load the phyloseq object resulting from Script 1:**

#### The file phy\_obj1\_core\_cleaned\_vsd1.Rdata is provided.

```
d_30 <- readRDS(file = "./phy_obj1_core_cleaned_vsd1.RData")
```

## **Boxplot of alpha-diversity per time point:**

#### The following code produces the basic Figure 1C.

```
plot.div <- ggplot(subset(sample_data(d_30),timepoint != "NA"), aes(x = timepoint, y =  InvSimpson)) + geom_boxplot(outlier.color="NA") + geom_jitter(size=3, shape = 21, width = 0.2)  + theme(axis.title.y = element_text(size=12, margin=margin(0,25,0,0), face = "bold"), axis.text.y = element_text(size=16), axis.text.x = element_text(size=14, face = "bold", angle = 35, vjust = 1, hjust = 1), axis.title.x = element_blank(), legend.text = element_text(size=14, face = "bold"), legend.title = element_text(face = "bold", size = 20),panel.grid.minor = element_blank(), panel.grid.major = element_blank(), panel.background = element_blank(), panel.border = element_rect(color = "black", fill= NA)) + labs(y="Inverse Simpson") + scale_x_discrete(labels=c("pre-HSCT", "day of HSCT", "week +1", "week +2", "week +3", "week +4", "week +5")) + stat_boxplot(geom ='errorbar') 

p_median <- plot.div+stat_summary(fun.y=median, geom="line", colour="black", size = 1.4, aes(group = 1)) + coord_trans(y = "log10")


print(p_median)
```

### **Comparison of alpha diversity at different time points using a Friedman test with Benjamini-Hochberg correction and a post-hoc Conover test:**

```
#subset h2:
div_fried <- as(sample_data(d_30)[,c("Patient_ID", "timepoint", "InvSimpson")], "data.frame")

#dcast to format the data as required for the Friedman test
div_fried_dcast <- dcast(div_fried, Patient_ID  ~ timepoint, value.var = "InvSimpson", mean, margins =TRUE)

#turn into a matrix: 
div_fried_dcast_ma <- as.matrix(div_fried_dcast)

#perfrom Friedman test.Week +5 needs to be excluded as there were too few observatons at this time point.
friedman.test(div_fried_dcast_ma[, c(2:7)], na.action = "na.omit", p.adjust.method = "BH") #no overall significant difference between timepoints
```

```
## 
##  Friedman rank sum test
## 
## data:  div_fried_dcast_ma[, c(2:7)]
## Friedman chi-squared = 8.8571, df = 5, p-value = 0.1149
```

```
#Post-hoc Conover test. Conover was chosen over Nemenyi as it allows for p-value correction for multiple testing.
#Therefore first convert the data to a numeric matrix:

div_fried_dcast_ma_num <- div_fried_dcast_ma

mode(div_fried_dcast_ma_num) <- "numeric" #convert character matrix to numeric matrix
```

```
## Warning in mde(x): NAs introduced by coercion
```

```
posthoc.friedman.conover.test(div_fried_dcast_ma_num[, c(2:7)],na.action = "na.omit", p.adjust.method = "BH")
```

```
## 
##  Pairwise comparisons using Conover's test for a two-way 
##                     balanced complete block design 
## 
## data:  div_fried_dcast_ma_num[, c(2:7)] 
## 
##    f01     w0      w1      w2      w3     
## w0 0.03520 -       -       -       -      
## w1 0.87249 0.04782 -       -       -      
## w2 0.00057 0.16067 0.00089 -       -      
## w3 2.4e-06 0.00653 4.1e-06 0.16067 -      
## w4 1.1e-14 1.4e-09 1.5e-14 1.4e-06 0.00036
## 
## P value adjustment method: BH
```

## **Plot stacked bar charts of microbial composition (relative abundance) on taxonomic family level for initial pattern identification**

#### The file phy\_obj1\_core\_cleaned.Rdata is provided.

```
phy_dat <- readRDS(file = "./phy_obj1_core_cleaned.Rdata")
```

#### The following code is modified after: Fukuyama J, Rumker L, Sankaran K, Jeganathan P, Dethlefsen L, Relman DA, et al. Multidomain analyses of a longitudinal human microbiome intestinal cleanout perturbation experiment. PLoS Comput Biol. 2017;13:e1005706. doi:10.1371/journal.pcbi.1005706.

### First, we combine the abundances, taxonomic information, and sample data in a data frame.

```
otu_counts <- data.frame(Sample_ID = sample_names(phy_dat), t(get_taxa(phy_dat))) %>%  tbl_df()

sample_df <- sample_data(phy_dat) %>% as.data.frame()

sample_df <- data.frame(Sample_ID = row.names(sample_df), sample_df)

taxa <- tax_table(phy_dat)@.Data %>%  tbl_df()
taxa$OTU <- taxa_names(phy_dat)


combined <- otu_counts %>%
  melt(
    id.vars = "Sample_ID",
    variable.name = "OTU"
  ) %>%
  left_join(sample_df) %>%
  left_join(taxa)
```

```
## Joining, by = "Sample_ID"
```

```
## Joining, by = "OTU"
```

```
## Warning: Column `OTU` joining factor and character vector, coercing into
## character vector
```

```
combined <- combined[! is.na(combined$timepoint),]
```

### Counts are summarized by family and time point and transformed to relative abundance. Additionally, we calculate one version grouped by patient ID, and another grouped by remission status.

```
combined$Family[is.na(combined$Family)] <- "unknown"

stacked_bar_data <- combined %>%
  group_by(timepoint, Family) %>%
  summarise(Family_total = sum(value)) %>%
  group_by(timepoint) %>%
  mutate(total = sum(Family_total), Family_frac = Family_total / total)


## Identify the 8 most abundant families
top_families <- combined %>%
  group_by(Family) %>%
  summarise(Family_total = sum(value)) %>%
  arrange(desc(Family_total)) %>%
  top_n(8) %>%
  dplyr::select(Family) %>%
  unlist()
```

```
## Selecting by Family_total
```

```
## Control the plot order in the legend
stacked_bar_data$Family[!(stacked_bar_data$Family %in% top_families)] <- "other"
stacked_bar_data$Family <- factor(
  stacked_bar_data$Family,
  levels = c(setdiff(top_families, "unknown"), "other")
) #when combined as 'unknown' unclassified families fall under ther top 8 (therefore they are not identified as different from 'top_families' by 'setdiff' and set to 'NA'). However, they do not represent the same family, which has to be considered. The plot below will be adjusted accordingly, so that they fall under 'other'.
```

### Stacked bar chart of the relative abundance per time point for the 12 most abundant taxonomic families in the gut.

#### The following code produces the basic Figure 1D.

```
my.col_x <- c("Enterobacteriaceae" = "#a6cee3", "Lactobacillaceae" = "#1f78b4", "Streptococcaceae" = "#b2df8a", "Staphylococcaceae" = "#33a02c", "Enterococcaceae" = "#fb9a99", "Lachnospiraceae" = "#e31a1c", "Erysipelotrichaceae" = "#fdbf6f", "Ruminococcaceae" = "#ff7f00", "other" = "grey")


ggplot(stacked_bar_data) +
  geom_bar(aes(x = timepoint, y = Family_frac, fill = Family), width = 1, stat = "identity") +
  scale_fill_manual(values = my.col_x, drop = FALSE) +
  guides(fill = guide_legend(override.aes = list(color = "black", size = 0.2), nrow = 4)) +
  labs(
    "x" = "Time point",
    "y" = "Proportion"
  ) +
  theme(
    panel.border = element_rect(fill = "transparent", size = 1),
    panel.background = element_rect(fill = "#F7F7F7", size = 1),
    legend.position = "bottom",
    legend.text = element_text(size = 9),  axis.text.x = element_text(size=14, face = "bold", angle = 35, vjust = 1, hjust = 1), axis.text.y = element_text(size=12, face = "bold"), axis.title.y = element_text(size=14, face = "bold")
  ) + scale_x_discrete(labels=c("pre-HSCT", "day of HSCT", "week +1", "week +2", "week +3", "week +4", "week +5"))
```

### Proportions of selected families

```
# Lachnospiraceae pre-HSCT
stacked_bar_data$Family_frac[stacked_bar_data$Family == "Lachnospiraceae" & stacked_bar_data$timepoint == "f01"]
```

```
## [1] 0.190559
```

```
# Average Lachnospiraceae w1 to w5
mean(stacked_bar_data$Family_frac[stacked_bar_data$Family == "Lachnospiraceae" & stacked_bar_data$timepoint %in% c("w1", "w2", "w3", "w4", "w5")])
```

```
## [1] 0.06149073
```

```
# Ruminococcaceae pre-HSCT
stacked_bar_data$Family_frac[stacked_bar_data$Family == "Ruminococcaceae" & stacked_bar_data$timepoint == "f01"]
```

```
## [1] 0.09554007
```

```
# Average Ruminococcaceae w1 to w5
mean(stacked_bar_data$Family_frac[stacked_bar_data$Family == "Ruminococcaceae" & stacked_bar_data$timepoint %in% c("w1", "w2", "w3", "w4", "w5")])
```

```
## [1] 0.016628
```

```
# Enterococcaceae pre-HSCT
stacked_bar_data$Family_frac[stacked_bar_data$Family == "Enterococcaceae" & stacked_bar_data$timepoint == "f01"]
```

```
## [1] 0.1739716
```

```
# Average Enterococcaceae w1 to w5
mean(stacked_bar_data$Family_frac[stacked_bar_data$Family == "Enterococcaceae" & stacked_bar_data$timepoint %in% c("w1", "w2", "w3", "w4", "w5")])
```

```
## [1] 0.3015398
```
