# Supplementary material for: Specific gut microbiome members are associated with distinct immune markers in pediatric allogeneic hematopoietic stem cell transplantation
Source: Microbiome. 2019 Sep 13;7:131. doi: 10.1186/s40168-019-0745-z (PMC6744702; doi:10.1186/s40168-019-0745-z)
Supplement: Supplementary file 13 — R data analysis report 3. Temporal patterns of immune markers and immune cells in HSCT patients. (HTML 1457 kb) [file 40168_2019_745_MOESM13_ESM.html]

Temporal patterns of immune markers and immune cells in HSCT patients


# Temporal patterns of immune markers and immune cells in HSCT patients

#### *Anna Caecilia Masche*

#### *March 2019*

This is the 3rd script used for data analysis and figure generation for the the manuscript by Masche et al. titled “Specific gut microbiome members are associated with distinct immune markers in allogeneic hematopoietic stem cell transplantation”.

This script and associated data are provided by Anna Cäcilia Masche, Susan Holmes, and Sünje Johanna Pamp.

These data and the associated script are licensed under the Creative Commons Attribution-ShareAlike 4.0 International License. To view a copy of this license, visit http://creativecommons.org/licenses/by-sa/4.0/ or send a letter to Creative Commons, PO Box 1866, Mountain View, CA 94042, USA.

Under the condition that appropriate credit is provided, you are free to: 1) Share, copy and redistribute the material in any medium or format 2) Adapt, remix, transform, and build upon the material for any purpose, even commercially.

To see the full license associated with attribution of this work, see the CC-By-CA license, see http://creativecommons.org/licenses/by-sa/4.0/.

The local filename is: Script3\_temporal\_pattern.Rmd.

## **Load required packages:**

```
library("ggplot2")
```

```
## Warning: package 'ggplot2' was built under R version 3.5.2
```

```
library("dplyr")
```

```
## Warning: package 'dplyr' was built under R version 3.5.2
```

```
## 
## Attaching package: 'dplyr'
```

```
## The following objects are masked from 'package:stats':
## 
##     filter, lag
```

```
## The following objects are masked from 'package:base':
## 
##     intersect, setdiff, setequal, union
```

```
library("gridExtra")
```

```
## 
## Attaching package: 'gridExtra'
```

```
## The following object is masked from 'package:dplyr':
## 
##     combine
```

```
library("reshape2")
```

```
## Warning: package 'reshape2' was built under R version 3.5.2
```

```
#install.packages("PMCMR")
library("PMCMR")
```

```
## PMCMR is superseded by PMCMRplus and will be no longer maintained. You may wish to install PMCMRplus instead.
```

```
library("DescTools")
```

```
## Warning: package 'DescTools' was built under R version 3.5.2
```

## **Load table:**

#### The file Data\_matrix\_37\_patients\_per\_day.txt is provided.

```
a <- read.table(file= "M:/Documents/Publications/Masche_R_Scripts_and_Data/Data/Data_matrix_37_patients_per_day.txt", sep = "\t", header = TRUE)
```

### **Exclude one patient for which the microbiome was characterized, but hBD2 was not measured (see Table S1):**

```
out <- c("P21")
a_2 <- droplevels(a[! a$Patient_ID %in% out, ])
```

### **Subset unique time points per patient:**

```
a_3 <- a_2[! duplicated(a_2[c("Patient_ID", "timepoint")]),]
```

## **Subset time points for C-reactive preotein (CRP):**

```
out2 <- c("NA", "x8months", "x9months", "xy10months", "xy11months", "y1year")

a_4 <- droplevels(a_3[! a_3$timepoint %in% out2, ])

a_4 <- a_4[!is.na(a_4$timepoint),]
```

### **Plot CRP levels per timepoint:**

```
plot.crp <- ggplot(a_4, aes(x = timepoint , y =  CRP_tp_mean)) + geom_boxplot(fill = "#94CF8F", outlier.color = "NA") + geom_jitter(size=0.5, shape = 21, width = 0.2, fill = "#2BA121", color= "#2BA121")  + theme(axis.title.y = element_text(size=10, margin=margin(0,5,0,0), face = "bold"), axis.text.y = element_text(size=10), axis.text.x = element_text(size=9, face = "bold", angle = 25, vjust = 1, hjust = 1), axis.title.x = element_blank(), legend.text = element_text(size=14, face = "bold"), legend.title = element_text(face = "bold", size = 20),panel.grid.minor = element_blank(), panel.grid.major = element_blank(), panel.background = element_blank(), panel.border = element_rect(color = "black", fill= NA)) + labs(y="CRP [mg/L]") + stat_boxplot(geom ='errorbar') + scale_y_continuous(breaks= c( 1, 2.5, 5, 10, 25, 50, 75, 125)) + scale_x_discrete(labels=c("pre-HSCT", "day of HSCT", "week +1", "week +2", "week +3", "week +4", "week +5", "week +6", "month +2", "month +3", "month +4", "month +5","month +6", "month +7")) + coord_trans(y = "log10")

plot.crp
```

```
## Warning: Removed 60 rows containing non-finite values (stat_boxplot).

## Warning: Removed 60 rows containing non-finite values (stat_boxplot).
```

```
## Warning: Removed 60 rows containing missing values (geom_point).
```

### **Comparison of CRP levels at different time points using a Friedman test with Benjamini-Hochberg correction and a post-hoc Conover test:**

```
#subset CRP:
crp_fried <- a_4[,c("Patient_ID", "timepoint", "CRP_tp_mean")]

#dcast to format the data as required for the Friedman test
crp_fried_dcast <- dcast(crp_fried, Patient_ID  ~ timepoint, value.var = "CRP_tp_mean", mean, margins =TRUE)

#turn into a matrix: 
crp_fried_dcast_ma <- as.matrix(crp_fried_dcast)

#perfrom Friedman test:
friedman.test(crp_fried_dcast_ma, na.action = "na.omit", p.adjust.method = "BH") #overall significant difference between timepoints
```

```
## 
##  Friedman rank sum test
## 
## data:  crp_fried_dcast_ma
## Friedman chi-squared = 44.767, df = 15, p-value = 8.332e-05
```

```
#Post-hoc Conover test. Conover was chosen over Nemenyi as it allows for p-value correction for multiple testing.
#Therefore first convert the data to a numeric matrix:

crp_fried_dcast_ma_num <- crp_fried_dcast_ma

mode(crp_fried_dcast_ma_num) <- "numeric" #convert character matrix to numeric matrix
```

```
## Warning in mde(x): NAs introduced by coercion
```

```
posthoc.friedman.conover.test(crp_fried_dcast_ma_num[, c(2:16)],na.action = "na.omit", p.adjust.method = "BH")
```

```
## 
##  Pairwise comparisons using Conover's test for a two-way 
##                     balanced complete block design 
## 
## data:  crp_fried_dcast_ma_num[, c(2:16)] 
## 
##          f01     w0      w1      w2      w3      w4      w5      w6     
## w0       0.10443 -       -       -       -       -       -       -      
## w1       0.40736 0.01189 -       -       -       -       -       -      
## w2       0.65783 0.03321 0.73736 -       -       -       -       -      
## w3       3.1e-07 1.5e-11 2.4e-05 4.0e-06 -       -       -       -      
## w4       < 2e-16 < 2e-16 3.2e-16 < 2e-16 5.2e-05 -       -       -      
## w5       < 2e-16 < 2e-16 4.8e-15 2.7e-16 0.00023 0.74292 -       -      
## w6       1.2e-12 < 2e-16 3.6e-10 3.2e-11 0.04502 0.05246 0.11385 -      
## x2months 1.3e-11 < 2e-16 3.0e-09 3.1e-10 0.09995 0.02218 0.05246 0.74292
## x3months 1.5e-08 3.5e-13 1.7e-06 2.4e-07 0.63178 0.00049 0.00177 0.14346
## x4months 1.7e-11 < 2e-16 4.0e-09 4.1e-10 0.10906 0.01948 0.04740 0.72432
## x5months 2.0e-08 4.9e-13 2.1e-06 3.1e-07 0.65783 0.00041 0.00149 0.13069
## x6months 2.4e-11 < 2e-16 5.3e-09 5.6e-10 0.11879 0.01707 0.04275 0.69092
## x7months 3.4e-08 0.00014 2.0e-10 1.9e-09 < 2e-16 < 2e-16 < 2e-16 < 2e-16
## (all)    < 2e-16 1.4e-12 < 2e-16 < 2e-16 < 2e-16 < 2e-16 < 2e-16 < 2e-16
##          x2months x3months x4months x5months x6months x7months
## w0       -        -        -        -        -        -       
## w1       -        -        -        -        -        -       
## w2       -        -        -        -        -        -       
## w3       -        -        -        -        -        -       
## w4       -        -        -        -        -        -       
## w5       -        -        -        -        -        -       
## w6       -        -        -        -        -        -       
## x2months -        -        -        -        -        -       
## x3months 0.27120  -        -        -        -        -       
## x4months 0.95834  0.28932  -        -        -        -       
## x5months 0.25378  0.95834  0.27120  -        -        -       
## x6months 0.94375  0.31147  0.95834  0.28932  -        -       
## x7months < 2e-16  < 2e-16  < 2e-16  < 2e-16  < 2e-16  -       
## (all)    < 2e-16  < 2e-16  < 2e-16  < 2e-16  < 2e-16  0.00095 
## 
## P value adjustment method: BH
```

#### Effect size

#### Kendall’s coefficient of concordance decsribes the agreement between subjects (Patient ID) and gives a value which ranges between 0 and 1. Kendall’s W of 1 indicates that all subjects ranked the time points in the same way and therefore were in complete agreement.

```
crp_fried_dcast_ma_df <- as.data.frame(crp_fried_dcast_ma[c(1:37),c(1:15)])

crp_fried_dcast_ma_df[2:15] <- lapply(crp_fried_dcast_ma_df[2:15], as.character)
crp_fried_dcast_ma_df[2:15] <- lapply(crp_fried_dcast_ma_df[2:15], as.numeric)


#Transpose, so that groups (time points) are in rows, and blocks (PatientID) are in columns. 
crp_fried_dcast_ma_df_t <- t(crp_fried_dcast_ma_df)
crp_fried_dcast_ma_df_t <- as.data.frame(crp_fried_dcast_ma_df_t)
colnames(crp_fried_dcast_ma_df_t) <- as.character(unlist(crp_fried_dcast_ma_df_t[1,]))
crp_fried_dcast_ma_df_t = crp_fried_dcast_ma_df_t[-1, ]


for(i in 1:37){
 crp_fried_dcast_ma_df_t[,i] <- rank(crp_fried_dcast_ma_df_t[,i], 
      na.last = "keep", ties.method = "average")
  } 

KendallW(crp_fried_dcast_ma_df_t,
         correct=FALSE,
         test=TRUE, na.rm = FALSE)
```

```
## Warning in KendallW(crp_fried_dcast_ma_df_t, correct = FALSE, test =
## TRUE, : Coefficient may be incorrect due to ties
```

```
## 
##  Kendall's coefficient of concordance W
## 
## data:  crp_fried_dcast_ma_df_t
## Kendall chi-squared = 157.32, df = 13, subjects = 14, raters = 37,
## p-value < 2.2e-16
## alternative hypothesis: W is greater 0
## sample estimates:
##         W 
## 0.3270664
```

#### Mean/median CRP by time point:

```
a_4 %>% group_by(timepoint) %>% dplyr::summarize(var = median(CRP_tp_mean, na.rm = TRUE))
```

```
## # A tibble: 14 x 2
##    timepoint   var
##    <fct>     <dbl>
##  1 f01       14.4 
##  2 w0        16.9 
##  3 w1        11.2 
##  4 w2        13.7 
##  5 w3         3.92
##  6 w4         1.46
##  7 w5         1.43
##  8 w6         1.4 
##  9 x2months   2.22
## 10 x3months   2.40
## 11 x4months   1.6 
## 12 x5months   2.65
## 13 x6months   2.35
## 14 x7months   3.9
```

```
a_4 %>% group_by(timepoint) %>% dplyr::summarize(var = min(CRP_tp_mean, na.rm = TRUE))
```

```
## # A tibble: 14 x 2
##    timepoint   var
##    <fct>     <dbl>
##  1 f01        1   
##  2 w0         1.22
##  3 w1         1   
##  4 w2         1   
##  5 w3         1   
##  6 w4         1   
##  7 w5         1   
##  8 w6         1   
##  9 x2months   1   
## 10 x3months   1   
## 11 x4months   1   
## 12 x5months   1   
## 13 x6months   1   
## 14 x7months   1
```

```
a_4 %>% group_by(timepoint) %>% dplyr::summarize(var = max(CRP_tp_mean, na.rm = TRUE))
```

```
## # A tibble: 14 x 2
##    timepoint   var
##    <fct>     <dbl>
##  1 f01        60.4
##  2 w0         85.3
##  3 w1        135. 
##  4 w2        110. 
##  5 w3         55.9
##  6 w4         26.5
##  7 w5         36.2
##  8 w6         23.6
##  9 x2months   43.1
## 10 x3months   65.9
## 11 x4months   86.1
## 12 x5months   33.1
## 13 x6months   26.1
## 14 x7months   98.7
```

## **Subset time points for monocytes:**

```
#calculate time point mean:
a_m_mean <- a_2 %>% dplyr::group_by(Patient_ID, timepoint) %>% dplyr::summarise(mono_tp_mean = mean(mono, na.rm = TRUE))

a_m_mean1 <- a_m_mean[!is.na(a_m_mean$timepoint),]
```

### **Plot monocytes:**

```
a_m_mean1$mono_tp_mean[a_m_mean1$mono_tp_mean == 0] <- 1 #replace 0s with 1 so the axis can be log transformed

plot.mono <- ggplot(a_m_mean1, aes(x = timepoint, y =  mono_tp_mean)) + geom_boxplot(fill = "#94CF8F", outlier.color = "NA") + geom_jitter(size=0.5, shape = 21, width = 0.2, fill = "#2BA121", color= "#2BA121")  + theme(axis.title.y = element_text(size=10, margin=margin(0,5,0,0), face = "bold"), axis.text.y = element_text(size=10), axis.text.x = element_text(size=9, face = "bold", angle = 25, vjust = 1, hjust = 1), axis.title.x = element_blank(), legend.text = element_text(size=14, face = "bold"), legend.title = element_text(face = "bold", size = 20),panel.grid.minor = element_blank(), panel.grid.major = element_blank(), panel.background = element_blank(), panel.border = element_rect(color = "black", fill= NA)) + labs(y="Monocyte count [x10^9/L]") + stat_boxplot(geom ='errorbar')  + coord_trans(y = "log10") + scale_y_continuous(breaks= c(0.01, 0.025, 0.05, 0.1, 0.25, 0.5, 1, 2.5)) + scale_x_discrete(labels=c("pre-HSCT", "day of HSCT", "week +1", "week +2", "week +3", "week +4", "week +5", "week +6", "month +2", "month +3", "month +4", "month +5","month +6", "month +7", "month +8", "month +9", "month +10", "month +11", "1 year"))  


plot.mono
```

```
## Warning: Removed 214 rows containing non-finite values (stat_boxplot).

## Warning: Removed 214 rows containing non-finite values (stat_boxplot).
```

```
## Warning: Removed 214 rows containing missing values (geom_point).
```

### **Comparison of monocyte count at different time points using a Friedman test with Benjamini-Hochberg correction and a post-hoc Conover test:**

```
#subset mono:
mono_fried <- a_m_mean[,c("Patient_ID", "timepoint", "mono_tp_mean")]

#dcast to format the data as required for the Friedman test
mono_fried_dcast <- dcast(mono_fried, Patient_ID  ~ timepoint, value.var = "mono_tp_mean", mean, margins =TRUE)

#turn into a matrix: 
mono_fried_dcast_ma <- as.matrix(mono_fried_dcast)

#perfrom Friedman test. Monocyte count on the day of HSCT, in week +1 and in week +2 were excluded as data was missing for ???40% of the patients leading to .
friedman.test(mono_fried_dcast_ma[,c(1:2, 6:20)], na.action = "na.omit", p.adjust.method = "BH") #no overall significant difference between timepoints
```

```
## 
##  Friedman rank sum test
## 
## data:  mono_fried_dcast_ma[, c(1:2, 6:20)]
## Friedman chi-squared = 21.412, df = 16, p-value = 0.1632
```

```
#Post-hoc Conover test. Conover was chosen over Nemenyi as it allows for p-value correction for multiple testing.
#Therefore first convert the data to a numeric matrix:

mono_fried_dcast_ma_num <- mono_fried_dcast_ma

mode(mono_fried_dcast_ma_num) <- "numeric" #convert character matrix to numeric matrix
```

```
## Warning in mde(x): NAs introduced by coercion
```

```
posthoc.friedman.conover.test(mono_fried_dcast_ma_num[, c(2:20)],na.action = "na.omit", p.adjust.method = "BH")
```

```
## 
##  Pairwise comparisons using Conover's test for a two-way 
##                     balanced complete block design 
## 
## data:  mono_fried_dcast_ma_num[, c(2:20)] 
## 
##            f01     w0      w1      w2      w3      w4      w5      w6     
## w0         0.04223 -       -       -       -       -       -       -      
## w1         1.3e-13 1.0e-07 -       -       -       -       -       -      
## w2         0.03236 0.93117 1.8e-07 -       -       -       -       -      
## w3         0.55766 0.19203 1.7e-11 0.15902 -       -       -       -      
## w4         0.01470 0.76129 8.4e-07 0.82166 0.08695 -       -       -      
## w5         0.00052 0.20612 9.1e-05 0.24750 0.00625 0.38444 -       -      
## w6         0.00261 0.41196 1.2e-05 0.47472 0.02272 0.65937 0.72533 -      
## x2months   0.05333 0.93117 6.2e-08 0.86369 0.22451 0.69449 0.17711 0.36077
## x3months   0.03349 0.93117 1.7e-07 0.98669 0.16305 0.82166 0.24209 0.46692
## x4months   0.00073 0.24209 6.1e-05 0.28811 0.00829 0.43739 0.93117 0.79244
## x5months   0.00680 0.59088 3.1e-06 0.65937 0.04751 0.83343 0.53728 0.82166
## x6months   0.01603 0.78376 7.3e-07 0.83343 0.09295 0.97911 0.36764 0.64655
## x7months   3.1e-06 0.01470 0.00680 0.01994 8.0e-05 0.04223 0.30025 0.14476
## x8months   1.5e-14 2.1e-08 0.82166 4.0e-08 2.5e-12 1.8e-07 2.5e-05 3.1e-06
## x9months   < 2e-16 3.5e-12 0.12249 7.3e-12 < 2e-16 4.6e-11 2.1e-08 1.5e-09
## xy10months < 2e-16 < 2e-16 0.00069 < 2e-16 < 2e-16 < 2e-16 2.3e-13 8.6e-15
## xy11months < 2e-16 < 2e-16 7.8e-07 < 2e-16 < 2e-16 < 2e-16 < 2e-16 < 2e-16
## y1year     < 2e-16 1.5e-11 0.18749 3.1e-11 4.3e-16 2.0e-10 7.3e-08 5.9e-09
##            x2months x3months x4months x5months x6months x7months x8months
## w0         -        -        -        -        -        -        -       
## w1         -        -        -        -        -        -        -       
## w2         -        -        -        -        -        -        -       
## w3         -        -        -        -        -        -        -       
## w4         -        -        -        -        -        -        -       
## w5         -        -        -        -        -        -        -       
## w6         -        -        -        -        -        -        -       
## x2months   -        -        -        -        -        -        -       
## x3months   0.87195  -        -        -        -        -        -       
## x4months   0.20612  0.28215  -        -        -        -        -       
## x5months   0.52909  0.65519  0.59935  -        -        -        -       
## x6months   0.71662  0.83016  0.41932  0.82166  -        -        -       
## x7months   0.01115  0.01922  0.26058  0.07817  0.03951  -        -       
## x8months   1.2e-08  3.7e-08  1.7e-05  7.3e-07  1.6e-07  0.00261  -       
## x9months   1.8e-12  6.7e-12  1.2e-08  2.4e-10  3.8e-11  8.5e-06  0.20612 
## xy10months < 2e-16  < 2e-16  1.2e-13  8.9e-16  < 2e-16  4.9e-10  0.00200 
## xy11months < 2e-16  < 2e-16  < 2e-16  < 2e-16  < 2e-16  1.7e-14  3.3e-06 
## y1year     8.0e-12  2.8e-11  4.3e-08  1.0e-09  1.6e-10  2.4e-05  0.29415 
##            x9months xy10months xy11months
## w0         -        -          -         
## w1         -        -          -         
## w2         -        -          -         
## w3         -        -          -         
## w4         -        -          -         
## w5         -        -          -         
## w6         -        -          -         
## x2months   -        -          -         
## x3months   -        -          -         
## x4months   -        -          -         
## x5months   -        -          -         
## x6months   -        -          -         
## x7months   -        -          -         
## x8months   -        -          -         
## x9months   -        -          -         
## xy10months 0.09928  -          -         
## xy11months 0.00133  0.17283    -         
## y1year     0.85538  0.05974    0.00058   
## 
## P value adjustment method: BH
```

#### Effect size

#### Kendall’s coefficient of concordance decsribes the agreement between subjects (Patient ID) and gives a value which ranges between 0 and 1. Kendall’s W of 1 indicates that all subjects ranked the time points in the same way and therefore were in complete agreement.

```
mono_fried_dcast_ma_df <- as.data.frame(mono_fried_dcast_ma[c(1:37),c(1:20)])

mono_fried_dcast_ma_df[2:20] <- lapply(mono_fried_dcast_ma_df[2:20], as.character)
mono_fried_dcast_ma_df[2:20] <- lapply(mono_fried_dcast_ma_df[2:20], as.numeric)


#Transpose, so that groups (time points) are in rows, and blocks (PatientID) are in columns. 
mono_fried_dcast_ma_df_t <- t(mono_fried_dcast_ma_df)
mono_fried_dcast_ma_df_t <- as.data.frame(mono_fried_dcast_ma_df_t)
colnames(mono_fried_dcast_ma_df_t) <- as.character(unlist(mono_fried_dcast_ma_df_t[1,]))
mono_fried_dcast_ma_df_t = mono_fried_dcast_ma_df_t[-1, ]


for(i in 1:37){
 mono_fried_dcast_ma_df_t[,i] <- rank(mono_fried_dcast_ma_df_t[,i], 
      na.last = "keep", ties.method = "average")
  } 

KendallW(mono_fried_dcast_ma_df_t,
         correct=FALSE,
         test=TRUE, na.rm = FALSE)
```

```
## Warning in KendallW(mono_fried_dcast_ma_df_t, correct = FALSE, test =
## TRUE, : Coefficient may be incorrect due to ties
```

```
## 
##  Kendall's coefficient of concordance W
## 
## data:  mono_fried_dcast_ma_df_t
## Kendall chi-squared = 182.3, df = 18, subjects = 19, raters = 37,
## p-value < 2.2e-16
## alternative hypothesis: W is greater 0
## sample estimates:
##         W 
## 0.2737214
```

#### Mean/median monocytes by time point:

```
a_m_mean %>% group_by(timepoint) %>% dplyr::summarize(var = median(mono_tp_mean, na.rm = TRUE))
```

```
## # A tibble: 20 x 2
##    timepoint     var
##    <fct>       <dbl>
##  1 f01        0.354 
##  2 w0         0.0175
##  3 w1         0.01  
##  4 w2         0.23  
##  5 w3         0.348 
##  6 w4         0.563 
##  7 w5         0.63  
##  8 w6         0.47  
##  9 x2months   0.486 
## 10 x3months   0.495 
## 11 x4months   0.48  
## 12 x5months   0.485 
## 13 x6months   0.47  
## 14 x7months   0.51  
## 15 x8months   0.52  
## 16 x9months   0.565 
## 17 xy10months 0.48  
## 18 xy11months 0.45  
## 19 y1year     0.54  
## 20 <NA>       0.5
```

```
a_m_mean %>% group_by(timepoint) %>% dplyr::summarize(var = min(mono_tp_mean, na.rm = TRUE))
```

```
## # A tibble: 20 x 2
##    timepoint     var
##    <fct>       <dbl>
##  1 f01        0.0767
##  2 w0         0     
##  3 w1         0     
##  4 w2         0.04  
##  5 w3         0.12  
##  6 w4         0.08  
##  7 w5         0.1   
##  8 w6         0.132 
##  9 x2months   0.0925
## 10 x3months   0.09  
## 11 x4months   0.162 
## 12 x5months   0.09  
## 13 x6months   0.02  
## 14 x7months   0.11  
## 15 x8months   0.16  
## 16 x9months   0.184 
## 17 xy10months 0.173 
## 18 xy11months 0.142 
## 19 y1year     0.12  
## 20 <NA>       0.0242
```

```
a_m_mean %>% group_by(timepoint) %>% dplyr::summarize(var = max(mono_tp_mean, na.rm = TRUE))
```

```
## # A tibble: 20 x 2
##    timepoint    var
##    <fct>      <dbl>
##  1 f01        2.85 
##  2 w0         0.59 
##  3 w1         0.016
##  4 w2         0.493
##  5 w3         0.839
##  6 w4         1.71 
##  7 w5         1.69 
##  8 w6         1.54 
##  9 x2months   1.30 
## 10 x3months   1.86 
## 11 x4months   1.37 
## 12 x5months   1.38 
## 13 x6months   1.29 
## 14 x7months   0.987
## 15 x8months   1.34 
## 16 x9months   1.17 
## 17 xy10months 1.26 
## 18 xy11months 1.31 
## 19 y1year     1.57 
## 20 <NA>       1.25
```

## **Citrulline:**

```
a_citr <- subset(a_3, select= c(Patient_ID, CitDay7, CitDay21, CitDayMinus7))

a_citr1 <- reshape2::melt(a_citr, id.vars = c("Patient_ID"), measure.vars = c("CitDay7", "CitDay21", "CitDayMinus7"), variable.name = "timepoint", value.name = "Citrulline_tp")

levels(a_citr1$timepoint)[levels(a_citr1$timepoint) == "CitDayMinus7"] <- "a-7"
levels(a_citr1$timepoint)[levels(a_citr1$timepoint) == "CitDay7"] <- "b7"
levels(a_citr1$timepoint)[levels(a_citr1$timepoint) == "CitDay21"] <- "c21"


a_citr2 <- a_citr1[! duplicated(a_citr1[c("Patient_ID", "timepoint")]),]

a_citr3<- arrange(a_citr2,timepoint)
```

### **Plot citrulline:**

```
plot.citr <- ggplot(a_citr3, aes(x = as.character(timepoint) , y =  Citrulline_tp)) + geom_boxplot(fill = "#94CF8F", outlier.color = "NA") + geom_jitter(size=0.5, shape = 21, width = 0.2, fill = "#2BA121", color= "#2BA121")  + theme(axis.title.y = element_text(size=10, margin=margin(0,5,0,0), face = "bold"), axis.text.y = element_text(size=10), axis.text.x = element_text(size=9, face = "bold", angle = 25, vjust = 1, hjust = 1), axis.title.x = element_blank(), legend.text = element_text(size=14, face = "bold"), legend.title = element_text(face = "bold", size = 20),panel.grid.minor = element_blank(), panel.grid.major = element_blank(), panel.background = element_blank(), panel.border = element_rect(color = "black", fill= NA)) + labs(y="Citrulline [µmol/L]") + stat_boxplot(geom ='errorbar') + scale_x_discrete(labels=c("pre-HSCT", "week +1", "week +3")) + scale_y_continuous(breaks = c(0, 2.5, 5, 7.5, 10, 12.5, 15, 17.5, 20, 22.5, 25, 27.5, 30)) 


plot.citr
```

```
## Warning: Removed 5 rows containing non-finite values (stat_boxplot).

## Warning: Removed 5 rows containing non-finite values (stat_boxplot).
```

```
## Warning: Removed 5 rows containing missing values (geom_point).
```

### **Comparison of citrulline levels at different time points using a Friedman test with Benjamini-Hochberg correction and a post-hoc Conover test:**

```
#subset citr:
citr_fried <- a_citr3[,c("Patient_ID", "timepoint", "Citrulline_tp")]

#dcast to format the data as required for the Friedman test
citr_fried_dcast <- dcast(citr_fried, Patient_ID  ~ timepoint, value.var = "Citrulline_tp", mean, margins =TRUE)

#turn into a matrix: 
citr_fried_dcast_ma <- as.matrix(citr_fried_dcast)

#perfrom Friedman test.
friedman.test(citr_fried_dcast_ma, na.action = "na.omit", p.adjust.method = "BH") #overall significant difference between timepoints
```

```
## 
##  Friedman rank sum test
## 
## data:  citr_fried_dcast_ma
## Friedman chi-squared = 92.319, df = 4, p-value < 2.2e-16
```

```
#Post-hoc Conover test. Conover was chosen over Nemenyi as it allows for p-value correction for multiple testing.
#Therefore first convert the data to a numeric matrix:

citr_fried_dcast_ma_num <- citr_fried_dcast_ma

mode(citr_fried_dcast_ma_num) <- "numeric" #convert character matrix to numeric matrix
```

```
## Warning in mde(x): NAs introduced by coercion
```

```
posthoc.friedman.conover.test(citr_fried_dcast_ma_num[, c(2:4)],na.action = "na.omit", p.adjust.method = "BH")
```

```
## 
##  Pairwise comparisons using Conover's test for a two-way 
##                     balanced complete block design 
## 
## data:  citr_fried_dcast_ma_num[, c(2:4)] 
## 
##     b7      c21    
## c21 < 2e-16 -      
## a-7 < 2e-16 4.7e-11
## 
## P value adjustment method: BH
```

#### Effect size

#### Kendall’s coefficient of concordance decsribes the agreement between subjects (Patient ID) and gives a value which ranges between 0 and 1. Kendall’s W of 1 indicates that all subjects ranked the time points in the same way and therefore were in complete agreement.

```
citr_fried_dcast_ma_df <- as.data.frame(citr_fried_dcast_ma[c(1:37),c(1:4)])

citr_fried_dcast_ma_df[2:4] <- lapply(citr_fried_dcast_ma_df[2:4], as.character)
citr_fried_dcast_ma_df[2:4] <- lapply(citr_fried_dcast_ma_df[2:4], as.numeric)


#Transpose, so that groups (time points) are in rows, and blocks (PatientID) are in columns. 
citr_fried_dcast_ma_df_t <- t(citr_fried_dcast_ma_df)
citr_fried_dcast_ma_df_t <- as.data.frame(citr_fried_dcast_ma_df_t)
colnames(citr_fried_dcast_ma_df_t) <- as.character(unlist(citr_fried_dcast_ma_df_t[1,]))
citr_fried_dcast_ma_df_t = citr_fried_dcast_ma_df_t[-1, ]


for(i in 1:37){
 citr_fried_dcast_ma_df_t[,i] <- rank(citr_fried_dcast_ma_df_t[,i], 
      na.last = "keep", ties.method = "average")
  } 

KendallW(citr_fried_dcast_ma_df_t,
         correct=FALSE,
         test=TRUE, na.rm = FALSE)
```

```
## Warning in KendallW(citr_fried_dcast_ma_df_t, correct = FALSE, test =
## TRUE, : Coefficient may be incorrect due to ties
```

```
## 
##  Kendall's coefficient of concordance W
## 
## data:  citr_fried_dcast_ma_df_t
## Kendall chi-squared = 23.365, df = 2, subjects = 3, raters = 37,
## p-value = 8.441e-06
## alternative hypothesis: W is greater 0
## sample estimates:
##         W 
## 0.3157414
```

#### Mean/median by time point:

```
a_citr3 %>% group_by(timepoint) %>% dplyr::summarize(var = median(Citrulline_tp, na.rm = TRUE))
```

```
## # A tibble: 3 x 2
##   timepoint   var
##   <fct>     <dbl>
## 1 b7         0   
## 2 c21        8.13
## 3 a-7       10.3
```

```
a_citr3 %>% group_by(timepoint) %>% dplyr::summarize(var = min(Citrulline_tp, na.rm = TRUE))
```

```
## # A tibble: 3 x 2
##   timepoint   var
##   <fct>     <dbl>
## 1 b7            0
## 2 c21           0
## 3 a-7           0
```

```
a_citr3 %>% group_by(timepoint) %>% dplyr::summarize(var = max(Citrulline_tp, na.rm = TRUE))
```

```
## # A tibble: 3 x 2
##   timepoint   var
##   <fct>     <dbl>
## 1 b7         13.8
## 2 c21        22.4
## 3 a-7        29.2
```

## **NK cells:**

```
a_nk <- subset(a_3, select= c(Patient_ID, n3p16p56_dag30, n3p16p56_dag60, n3p16p56_dag90, n3p16p56_dag180))
 
a_nk1 <- reshape2::melt(a_nk, id.vars = c("Patient_ID"), measure.vars = c("n3p16p56_dag30", "n3p16p56_dag60", "n3p16p56_dag90", "n3p16p56_dag180"), variable.name = "timepoint", value.name = "NK_tp")

levels(a_nk1$timepoint)[levels(a_nk1$timepoint) == "n3p16p56_dag30"] <- "30"
levels(a_nk1$timepoint)[levels(a_nk1$timepoint) == "n3p16p56_dag60"] <- "60"
levels(a_nk1$timepoint)[levels(a_nk1$timepoint) == "n3p16p56_dag90"] <- "90"
levels(a_nk1$timepoint)[levels(a_nk1$timepoint) == "n3p16p56_dag180"] <- "x180"


a_nk2 <- a_nk1[! duplicated(a_nk1[c("Patient_ID", "timepoint")]),]
```

### **Plot NK cells:**

```
plot.NK <- ggplot(a_nk2, aes(x = as.character(timepoint) , y =  NK_tp)) + geom_boxplot(fill = "#94CF8F", outlier.color = "NA") + geom_jitter(size=0.5, shape = 21, width = 0.2, fill = "#2BA121", color= "#2BA121")  + theme(axis.title.y = element_text(size=10, margin=margin(0,5,0,0), face = "bold"), axis.text.y = element_text(size=10), axis.text.x = element_text(size=9, face = "bold", angle = 25, vjust = 1, hjust = 1), axis.title.x = element_blank(), legend.text = element_text(size=14, face = "bold"), legend.title = element_text(face = "bold", size = 20),panel.grid.minor = element_blank(), panel.grid.major = element_blank(), panel.background = element_blank(), panel.border = element_rect(color = "black", fill= NA)) + labs(y="NK cell count [x10^9/L]") + stat_boxplot(geom ='errorbar') + scale_x_discrete(labels=c("month +1", "month +2", "month +3","month +6")) + scale_y_continuous(breaks= c(0.001, 0.01, 0.05, 0.1, 0.2, 0.4, 0.8, 1.6)) + coord_trans(y = "log10")


plot.NK
```

```
## Warning: Removed 27 rows containing non-finite values (stat_boxplot).

## Warning: Removed 27 rows containing non-finite values (stat_boxplot).
```

```
## Warning: Removed 27 rows containing missing values (geom_point).
```

### **Comparison of NK cell counts at different time points using a Friedman test with Benjamini-Hochberg correction and a post-hoc Conover test:**

```
#subset NK:
NK_fried <- a_nk2[,c("Patient_ID", "timepoint", "NK_tp")]

#dcast to format the data as required for the Friedman test
NK_fried_dcast <- dcast(NK_fried, Patient_ID  ~ timepoint, value.var = "NK_tp", mean, margins =TRUE)

#turn into a matrix: 
NK_fried_dcast_ma <- as.matrix(NK_fried_dcast)

#perfrom Friedman test.
friedman.test(NK_fried_dcast_ma, na.action = "na.omit", p.adjust.method = "BH") #overall significant difference between timepoints
```

```
## 
##  Friedman rank sum test
## 
## data:  NK_fried_dcast_ma
## Friedman chi-squared = 49.463, df = 5, p-value = 1.785e-09
```

```
#Post-hoc Conover test. Conover was chosen over Nemenyi as it allows for p-value correction for multiple testing.
#Therefore first convert the data to a numeric matrix:

NK_fried_dcast_ma_num <- NK_fried_dcast_ma

mode(NK_fried_dcast_ma_num) <- "numeric" #convert character matrix to numeric matrix
```

```
## Warning in mde(x): NAs introduced by coercion
```

```
posthoc.friedman.conover.test(NK_fried_dcast_ma_num[, c(2:5)],na.action = "na.omit", p.adjust.method = "BH")
```

```
## 
##  Pairwise comparisons using Conover's test for a two-way 
##                     balanced complete block design 
## 
## data:  NK_fried_dcast_ma_num[, c(2:5)] 
## 
##      30      60      90    
## 60   2.6e-08 -       -     
## 90   4.6e-14 0.0077  -     
## x180 < 2e-16 1.4e-07 0.0040
## 
## P value adjustment method: BH
```

#### Effect size

#### Kendall’s coefficient of concordance decsribes the agreement between subjects (Patient ID) and gives a value which ranges between 0 and 1. Kendall’s W of 1 indicates that all subjects ranked the time points in the same way and therefore were in complete agreement.

```
NK_fried_dcast_ma_df <- as.data.frame(NK_fried_dcast_ma[c(1:37),c(1:5)])

NK_fried_dcast_ma_df[2:5] <- lapply(NK_fried_dcast_ma_df[2:5], as.character)
NK_fried_dcast_ma_df[2:5] <- lapply(NK_fried_dcast_ma_df[2:5], as.numeric)


#Transpose, so that groups (time points) are in rows, and NKlocks (PatientID) are in columns. 
NK_fried_dcast_ma_df_t <- t(NK_fried_dcast_ma_df)
NK_fried_dcast_ma_df_t <- as.data.frame(NK_fried_dcast_ma_df_t)
colnames(NK_fried_dcast_ma_df_t) <- as.character(unlist(NK_fried_dcast_ma_df_t[1,]))
NK_fried_dcast_ma_df_t = NK_fried_dcast_ma_df_t[-1, ]


for(i in 1:37){
 NK_fried_dcast_ma_df_t[,i] <- rank(NK_fried_dcast_ma_df_t[,i], 
      na.last = "keep", ties.method = "average")
  } 

KendallW(NK_fried_dcast_ma_df_t,
         correct=FALSE,
         test=TRUE, na.rm = FALSE)
```

```
## Warning in KendallW(NK_fried_dcast_ma_df_t, correct = FALSE, test = TRUE, :
## Coefficient may be incorrect due to ties
```

```
## 
##  Kendall's coefficient of concordance W
## 
## data:  NK_fried_dcast_ma_df_t
## Kendall chi-squared = 13.897, df = 3, subjects = 4, raters = 37,
## p-value = 0.003048
## alternative hypothesis: W is greater 0
## sample estimates:
##         W 
## 0.1252009
```

## **B cells:**

```
a_allB <- subset(a_3, select= c(Patient_ID, p45p19_dag30, p45p19_dag60, p45p19_dag90, p45p19_dag180))
 
a_allB1 <- reshape2::melt(a_allB, id.vars = c("Patient_ID"), measure.vars = c("p45p19_dag30", "p45p19_dag60", "p45p19_dag90", "p45p19_dag180"), variable.name = "timepoint", value.name = "allB_tp")

levels(a_allB1$timepoint)[levels(a_allB1$timepoint) == "p45p19_dag30"] <- "30"
levels(a_allB1$timepoint)[levels(a_allB1$timepoint) == "p45p19_dag60"] <- "60"
levels(a_allB1$timepoint)[levels(a_allB1$timepoint) == "p45p19_dag90"] <- "90"
levels(a_allB1$timepoint)[levels(a_allB1$timepoint) == "p45p19_dag180"] <- "x180"


a_allB2 <- a_allB1[! duplicated(a_allB1[c("Patient_ID", "timepoint")]),]
```

### **Plot B cells:**

```
plot.allB <- ggplot(a_allB2, aes(x = as.character(timepoint) , y =  allB_tp)) + geom_boxplot(fill = "#94CF8F", outlier.color = "NA") + geom_jitter(size=0.5, shape = 21, width = 0.2, fill = "#2BA121", color= "#2BA121")  + theme(axis.title.y = element_text(size=10, margin=margin(0,5,0,0), face = "bold"), axis.text.y = element_text(size=10), axis.text.x = element_text(size=9, face = "bold", angle = 25, vjust = 1, hjust = 1), axis.title.x = element_blank(), legend.text = element_text(size=14, face = "bold"), legend.title = element_text(face = "bold", size = 20),panel.grid.minor = element_blank(), panel.grid.major = element_blank(), panel.background = element_blank(), panel.border = element_rect(color = "black", fill= NA)) + labs(y="Total B cell count  [x10^9/L]") + stat_boxplot(geom ='errorbar') + scale_x_discrete(labels=c("month +1", "month +2", "month +3","month +6")) + scale_y_continuous(breaks= c(0.0002, 0.001, 0.005, 0.015, 0.05, 0.1, 0.2, 0.5, 1, 2, 3.5)) + coord_trans(y = "log10")


plot.allB
```

```
## Warning: Removed 27 rows containing non-finite values (stat_boxplot).

## Warning: Removed 27 rows containing non-finite values (stat_boxplot).
```

```
## Warning: Removed 27 rows containing missing values (geom_point).
```

### **Comparison of B cell counts at different time points using a Friedman test with Benjamini-Hochberg correction and a post-hoc Conover test:**

```
#subset B:
B_fried <- a_allB2[,c("Patient_ID", "timepoint", "allB_tp")]

#dcast to format the data as required for the Friedman test
B_fried_dcast <- dcast(B_fried, Patient_ID  ~ timepoint, value.var = "allB_tp", mean, margins =TRUE)

#turn into a matrix: 
B_fried_dcast_ma <- as.matrix(B_fried_dcast)

#perfrom Friedman test.
friedman.test(B_fried_dcast_ma, na.action = "na.omit", p.adjust.method = "BH") #overall significant difference between timepoints
```

```
## 
##  Friedman rank sum test
## 
## data:  B_fried_dcast_ma
## Friedman chi-squared = 73.517, df = 5, p-value = 1.897e-14
```

```
#Post-hoc Conover test. Conover was chosen over Nemenyi as it allows for p-value correction for multiple testing.
#Therefore first convert the data to a numeric matrix:

B_fried_dcast_ma_num <- B_fried_dcast_ma

mode(B_fried_dcast_ma_num) <- "numeric" #convert character matrix to numeric matrix
```

```
## Warning in mde(x): NAs introduced by coercion
```

```
posthoc.friedman.conover.test(B_fried_dcast_ma_num[, c(2:5)],na.action = "na.omit", p.adjust.method = "BH")
```

```
## 
##  Pairwise comparisons using Conover's test for a two-way 
##                     balanced complete block design 
## 
## data:  B_fried_dcast_ma_num[, c(2:5)] 
## 
##      30      60      90     
## 60   < 2e-16 -       -      
## 90   < 2e-16 6.5e-09 -      
## x180 < 2e-16 < 2e-16 < 2e-16
## 
## P value adjustment method: BH
```

#### Effect size

#### Kendall’s coefficient of concordance decsribes the agreement between subjects (Patient ID) and gives a value which ranges between 0 and 1. Kendall’s W of 1 indicates that all subjects ranked the time points in the same way and therefore were in complete agreement.

```
B_fried_dcast_ma_df <- as.data.frame(B_fried_dcast_ma[c(1:37),c(1:5)])

B_fried_dcast_ma_df[2:5] <- lapply(B_fried_dcast_ma_df[2:5], as.character)
B_fried_dcast_ma_df[2:5] <- lapply(B_fried_dcast_ma_df[2:5], as.numeric)


#Transpose, so that groups (time points) are in rows, and blocks (PatientID) are in columns. 
B_fried_dcast_ma_df_t <- t(B_fried_dcast_ma_df)
B_fried_dcast_ma_df_t <- as.data.frame(B_fried_dcast_ma_df_t)
colnames(B_fried_dcast_ma_df_t) <- as.character(unlist(B_fried_dcast_ma_df_t[1,]))
B_fried_dcast_ma_df_t = B_fried_dcast_ma_df_t[-1, ]


for(i in 1:37){
 B_fried_dcast_ma_df_t[,i] <- rank(B_fried_dcast_ma_df_t[,i], 
      na.last = "keep", ties.method = "average")
  } 

KendallW(B_fried_dcast_ma_df_t,
         correct=FALSE,
         test=TRUE, na.rm = FALSE)
```

```
## Warning in KendallW(B_fried_dcast_ma_df_t, correct = FALSE, test = TRUE, :
## Coefficient may be incorrect due to ties
```

```
## 
##  Kendall's coefficient of concordance W
## 
## data:  B_fried_dcast_ma_df_t
## Kendall chi-squared = 55.8, df = 3, subjects = 4, raters = 37,
## p-value = 4.635e-12
## alternative hypothesis: W is greater 0
## sample estimates:
##         W 
## 0.5027027
```

#### Mean/median per timepoint:

```
a_allB2 %>% group_by(timepoint) %>% dplyr::summarize(var = median(allB_tp, na.rm = TRUE))
```

```
## # A tibble: 4 x 2
##   timepoint    var
##   <fct>      <dbl>
## 1 30        0.003 
## 2 60        0.0485
## 3 90        0.098 
## 4 x180      0.255
```

```
a_allB2 %>% group_by(timepoint) %>% dplyr::summarize(var = min(allB_tp, na.rm = TRUE))
```

```
## # A tibble: 4 x 2
##   timepoint    var
##   <fct>      <dbl>
## 1 30        0.0002
## 2 60        0.001 
## 3 90        0.001 
## 4 x180      0.0004
```

```
a_allB2 %>% group_by(timepoint) %>% dplyr::summarize(var = max(allB_tp, na.rm = TRUE))
```

```
## # A tibble: 4 x 2
##   timepoint   var
##   <fct>     <dbl>
## 1 30        0.057
## 2 60        2.3  
## 3 90        0.68 
## 4 x180      1.6
```

## **CD4+ T cells:**

```
a_cd4T <- subset(a_3, select= c(Patient_ID, p3p4_dag30, p3p4_dag60, p3p4_dag90, p3p4_dag180))
 
a_cd4T1 <- reshape2::melt(a_cd4T, id.vars = c("Patient_ID"), measure.vars = c("p3p4_dag30", "p3p4_dag60", "p3p4_dag90", "p3p4_dag180"), variable.name = "timepoint", value.name = "cd4T_tp")

levels(a_cd4T1$timepoint)[levels(a_cd4T1$timepoint) == "p3p4_dag30"] <- "30"
levels(a_cd4T1$timepoint)[levels(a_cd4T1$timepoint) == "p3p4_dag60"] <- "60"
levels(a_cd4T1$timepoint)[levels(a_cd4T1$timepoint) == "p3p4_dag90"] <- "90"
levels(a_cd4T1$timepoint)[levels(a_cd4T1$timepoint) == "p3p4_dag180"] <- "x180"


a_cd4T2 <- a_cd4T1[! duplicated(a_cd4T1[c("Patient_ID", "timepoint")]),]
```

### **Plot CD4+ T cells:**

```
plot.cd4T <- ggplot(a_cd4T2, aes(x = as.character(timepoint) , y =  cd4T_tp)) + geom_boxplot(fill = "#94CF8F", outlier.color = "NA") + geom_jitter(size=0.5, shape = 21, width = 0.2, fill = "#2BA121", color= "#2BA121")  + theme(axis.title.y = element_text(size=10, margin=margin(0,5,0,0), face = "bold"), axis.text.y = element_text(size=10), axis.text.x = element_text(size=9, face = "bold", angle = 25, vjust = 1, hjust = 1), axis.title.x = element_blank(), legend.text = element_text(size=14, face = "bold"), legend.title = element_text(face = "bold", size = 20),panel.grid.minor = element_blank(), panel.grid.major = element_blank(), panel.background = element_blank(), panel.border = element_rect(color = "black", fill= NA)) + labs(y="CD4+ T cell count  [x10^9/L]") + stat_boxplot(geom ='errorbar') + scale_x_discrete(labels=c("month +1", "month +2", "month +3","month +6")) + scale_y_continuous(breaks= c(0.001, 0.005, 0.015, 0.05, 0.1, 0.2, 0.5,1, 1.5)) + coord_trans(y = "log10")

plot.cd4T
```

```
## Warning: Removed 27 rows containing non-finite values (stat_boxplot).

## Warning: Removed 27 rows containing non-finite values (stat_boxplot).
```

```
## Warning: Removed 27 rows containing missing values (geom_point).
```

### **Comparison of CD4+ T cell counts at different time points using a Friedman test with Benjamini-Hochberg correction and a post-hoc Conover test:**

```
#subset cd4:
cd4_fried <- a_cd4T2[,c("Patient_ID", "timepoint", "cd4T_tp")]

#dcast to format the data as required for the Friedman test
cd4_fried_dcast <- dcast(cd4_fried, Patient_ID  ~ timepoint, value.var = "cd4T_tp", mean, margins =TRUE)

#turn into a matrix: 
cd4_fried_dcast_ma <- as.matrix(cd4_fried_dcast)

#perfrom Friedman test.
friedman.test(cd4_fried_dcast_ma, na.action = "na.omit", p.adjust.method = "BH") #overall significant difference between timepoints
```

```
## 
##  Friedman rank sum test
## 
## data:  cd4_fried_dcast_ma
## Friedman chi-squared = 69.822, df = 5, p-value = 1.116e-13
```

```
#Post-hoc Conover test. Conover was chosen over Nemenyi as it allows for p-value correction for multiple testing.
#Therefore first convert the data to a numeric matrix:

cd4_fried_dcast_ma_num <- cd4_fried_dcast_ma

mode(cd4_fried_dcast_ma_num) <- "numeric" #convert character matrix to numeric matrix
```

```
## Warning in mde(x): NAs introduced by coercion
```

```
posthoc.friedman.conover.test(cd4_fried_dcast_ma_num[, c(2:5)],na.action = "na.omit", p.adjust.method = "BH")
```

```
## 
##  Pairwise comparisons using Conover's test for a two-way 
##                     balanced complete block design 
## 
## data:  cd4_fried_dcast_ma_num[, c(2:5)] 
## 
##      30      60      90     
## 60   5.8e-15 -       -      
## 90   < 2e-16 4.3e-13 -      
## x180 < 2e-16 < 2e-16 < 2e-16
## 
## P value adjustment method: BH
```

#### Effect size

#### Kendall’s coefficient of concordance decsribes the agreement between subjects (Patient ID) and gives a value which ranges between 0 and 1. Kendall’s W of 1 indicates that all subjects ranked the time points in the same way and therefore were in complete agreement.

```
cd4_fried_dcast_ma_df <- as.data.frame(cd4_fried_dcast_ma[c(1:37),c(1:5)])

cd4_fried_dcast_ma_df[2:5] <- lapply(cd4_fried_dcast_ma_df[2:5], as.character)
cd4_fried_dcast_ma_df[2:5] <- lapply(cd4_fried_dcast_ma_df[2:5], as.numeric)


#Transpose, so that groups (time points) are in rows, and blocks (PatientID) are in columns. 
cd4_fried_dcast_ma_df_t <- t(cd4_fried_dcast_ma_df)
cd4_fried_dcast_ma_df_t <- as.data.frame(cd4_fried_dcast_ma_df_t)
colnames(cd4_fried_dcast_ma_df_t) <- as.character(unlist(cd4_fried_dcast_ma_df_t[1,]))
cd4_fried_dcast_ma_df_t = cd4_fried_dcast_ma_df_t[-1, ]


for(i in 1:37){
 cd4_fried_dcast_ma_df_t[,i] <- rank(cd4_fried_dcast_ma_df_t[,i], 
      na.last = "keep", ties.method = "average")
  } 

KendallW(cd4_fried_dcast_ma_df_t,
         correct=FALSE,
         test=TRUE, na.rm = FALSE)
```

```
## Warning in KendallW(cd4_fried_dcast_ma_df_t, correct = FALSE, test =
## TRUE, : Coefficient may be incorrect due to ties
```

```
## 
##  Kendall's coefficient of concordance W
## 
## data:  cd4_fried_dcast_ma_df_t
## Kendall chi-squared = 51.138, df = 3, subjects = 4, raters = 37,
## p-value = 4.572e-11
## alternative hypothesis: W is greater 0
## sample estimates:
##         W 
## 0.4607012
```

#### Mean/median CD4+ T cell counts per time point

```
a_cd4T2 %>% group_by(timepoint) %>% dplyr::summarize(var = median(cd4T_tp, na.rm = TRUE))
```

```
## # A tibble: 4 x 2
##   timepoint    var
##   <fct>      <dbl>
## 1 30        0.0635
## 2 60        0.1   
## 3 90        0.13  
## 4 x180      0.275
```

```
a_cd4T2 %>% group_by(timepoint) %>% dplyr::summarize(var = min(cd4T_tp, na.rm = TRUE))
```

```
## # A tibble: 4 x 2
##   timepoint   var
##   <fct>     <dbl>
## 1 30        0.002
## 2 60        0.001
## 3 90        0.008
## 4 x180      0.041
```

```
a_cd4T2 %>% group_by(timepoint) %>% dplyr::summarize(var = max(cd4T_tp, na.rm = TRUE))
```

```
## # A tibble: 4 x 2
##   timepoint   var
##   <fct>     <dbl>
## 1 30         0.26
## 2 60         0.35
## 3 90         1.7 
## 4 x180       0.59
```

## **hBD2:**

#### Here we are using a data set that also contains the healthy controls (for hBD2), the other numbers are the same as in the data set used above.

```
h2_data <- read.table(file= "M:/Documents/Publications/Masche_R_Scripts_and_Data/Data/hBD2_with_healthy_controls.txt", header = TRUE, sep="")
```

The following code produces the basic Figure 1B.

```
h2_data$Mean_estimate_dilution_considered[h2_data$Mean_estimate_dilution_considered == 0] <- 1 #replace 0s with 1 so the axis can be log transformed

plot.h2 <- ggplot(subset(h2_data, timepoint != "NA"), aes(x = timepoint, y =  Mean_estimate_dilution_considered)) + geom_boxplot(fill = "#94CF8F", outlier.color = "NA") + geom_jitter(size=0.5, shape = 21, width = 0.2, fill = "#2BA121", color= "#2BA121")  + theme(axis.title.y = element_text(size=10, margin=margin(0,5,0,0), face = "bold"), axis.text.y = element_text(size=10), axis.text.x = element_text(size=9, face = "bold", angle = 25, vjust = 1, hjust = 1), axis.title.x = element_blank(), legend.text = element_text(size=14, face = "bold"), legend.title = element_text(face = "bold", size = 20),panel.grid.minor = element_blank(), panel.grid.major = element_blank(), panel.background = element_blank(), panel.border = element_rect(color = "black", fill= NA)) + labs(y="hBD2 [pg/ml]") + scale_x_discrete(labels=c("pre-HSCT", "day of HSCT", "week +1", "week +2", "week +3", "month +1", "month +2", "month +3", "Healthy controls")) + stat_boxplot(geom ='errorbar') + coord_trans(y = "log10")+ scale_y_continuous(breaks =c(1, 10, 50,100, 250, 500, 1000,2500,5000, 10000, 25000, 50000, 175000)) 

plot.h2
```

### **Comparison of hBD2 levels at different time points using a Friedman test with Benjamini-Hochberg correction and a post-hoc Conover test:**

```
#subset h2:
h2_fried <- h2_data[,c("Patient_ID", "timepoint", "Mean_estimate_dilution_considered")]

#dcast to format the data as required for the Friedman test
h2_fried_dcast <- dcast(h2_fried, Patient_ID  ~ timepoint, value.var = "Mean_estimate_dilution_considered", mean, margins =TRUE)

#turn into a matrix: 
h2_fried_dcast_ma <- as.matrix(h2_fried_dcast)

#perfrom Friedman test.
friedman.test(h2_fried_dcast_ma[, c(1:9)], na.action = "na.omit", p.adjust.method = "BH") #overall significant difference between timepoints
```

```
## 
##  Friedman rank sum test
## 
## data:  h2_fried_dcast_ma[, c(1:9)]
## Friedman chi-squared = 115.03, df = 8, p-value < 2.2e-16
```

```
#Post-hoc Conover test. Conover was chosen over Nemenyi as it allows for p-value correction for multiple testing.
#Therefore first convert the data to a numeric matrix:

h2_fried_dcast_ma_num <- h2_fried_dcast_ma

mode(h2_fried_dcast_ma_num) <- "numeric" #convert character matrix to numeric matrix
```

```
## Warning in mde(x): NAs introduced by coercion
```

```
posthoc.friedman.conover.test(h2_fried_dcast_ma_num[, c(2:9)],na.action = "na.omit", p.adjust.method = "BH")
```

```
## 
##  Pairwise comparisons using Conover's test for a two-way 
##                     balanced complete block design 
## 
## data:  h2_fried_dcast_ma_num[, c(2:9)] 
## 
##      f01     w0      w1      w2      w3      w4      w9     
## w0   2.7e-06 -       -       -       -       -       -      
## w1   0.00648 0.03831 -       -       -       -       -      
## w2   0.25795 0.00030 0.12880 -       -       -       -      
## w3   0.00010 0.39049 0.25795 0.00648 -       -       -      
## w4   4.9e-06 0.81738 0.06827 0.00072 0.53112 -       -      
## w9   0.03831 0.00648 0.53112 0.39049 0.06827 0.01409 -      
## xw13 2.7e-06 0.91854 0.04666 0.00038 0.43539 0.86896 0.00818
## 
## P value adjustment method: BH
```

#### Effect size

#### Kendall’s coefficient of concordance decsribes the agreement between subjects (Patient ID) and gives a value which ranges between 0 and 1. Kendall’s W of 1 indicates that all subjects ranked the time points in the same way and therefore were in complete agreement.

```
h2_fried_dcast_ma_df <- as.data.frame(h2_fried_dcast_ma[c(11:47),c(1:9)])#w/o healthy controls

h2_fried_dcast_ma_df[2:9] <- lapply(h2_fried_dcast_ma_df[2:9], as.character)
h2_fried_dcast_ma_df[2:9] <- lapply(h2_fried_dcast_ma_df[2:9], as.numeric)


#Transpose, so that groups (time points) are in rows, and blocks (PatientID) are in columns. 
h2_fried_dcast_ma_df_t <- t(h2_fried_dcast_ma_df)
h2_fried_dcast_ma_df_t <- as.data.frame(h2_fried_dcast_ma_df_t)
colnames(h2_fried_dcast_ma_df_t) <- as.character(unlist(h2_fried_dcast_ma_df_t[1,]))
h2_fried_dcast_ma_df_t = h2_fried_dcast_ma_df_t[-1, ]


for(i in 1:37){
 h2_fried_dcast_ma_df_t[,i] <- rank(h2_fried_dcast_ma_df_t[,i], 
      na.last = "keep", ties.method = "average")
  } 

KendallW(h2_fried_dcast_ma_df_t,
         correct=FALSE,
         test=TRUE, na.rm = FALSE)
```

```
## Warning in KendallW(h2_fried_dcast_ma_df_t, correct = FALSE, test = TRUE, :
## Coefficient may be incorrect due to ties
```

```
## 
##  Kendall's coefficient of concordance W
## 
## data:  h2_fried_dcast_ma_df_t
## Kendall chi-squared = 154.47, df = 7, subjects = 8, raters = 37,
## p-value < 2.2e-16
## alternative hypothesis: W is greater 0
## sample estimates:
##        W 
## 0.596412
```

### Compare hBD2 at each individual time point to the healthy controls by a Wilcoxon test (as healthy controls are not a repeated measurement relating to the other samples, but rather a set of different individuals)

```
wilcox.test(Mean_estimate_dilution_considered ~ timepoint, data= h2_data[h2_data$timepoint %in% c("f01", "yHC_unthawed_unspiked"),], na.action = "na.omit", p.adjust.method = "BH")
```

```
## Warning in wilcox.test.default(x = c(266, 7147, 1201, 104, 462, 40859,
## 32, : cannot compute exact p-value with ties
```

```
## 
##  Wilcoxon rank sum test with continuity correction
## 
## data:  Mean_estimate_dilution_considered by timepoint
## W = 172, p-value = 0.7452
## alternative hypothesis: true location shift is not equal to 0
```

```
wilcox.test(Mean_estimate_dilution_considered ~ timepoint, data= h2_data[h2_data$timepoint %in% c("w0", "yHC_unthawed_unspiked"),], na.action = "na.omit", p.adjust.method = "BH")
```

```
## 
##  Wilcoxon rank sum test
## 
## data:  Mean_estimate_dilution_considered by timepoint
## W = 174, p-value = 0.9232
## alternative hypothesis: true location shift is not equal to 0
```

```
wilcox.test(Mean_estimate_dilution_considered ~ timepoint, data= h2_data[h2_data$timepoint %in% c("w1", "yHC_unthawed_unspiked"),], na.action = "na.omit", p.adjust.method = "BH")
```

```
## Warning in wilcox.test.default(x = c(2066, 4038, 398, 507, 6889, 23069, :
## cannot compute exact p-value with ties
```

```
## 
##  Wilcoxon rank sum test with continuity correction
## 
## data:  Mean_estimate_dilution_considered by timepoint
## W = 185, p-value = 0.9046
## alternative hypothesis: true location shift is not equal to 0
```

```
wilcox.test(Mean_estimate_dilution_considered ~ timepoint, data= h2_data[h2_data$timepoint %in% c("w2", "yHC_unthawed_unspiked"),], na.action = "na.omit", p.adjust.method = "BH")
```

```
## Warning in wilcox.test.default(x = c(1072, 1690, 953, 6966, 22456, 93,
## 983, : cannot compute exact p-value with ties
```

```
## 
##  Wilcoxon rank sum test with continuity correction
## 
## data:  Mean_estimate_dilution_considered by timepoint
## W = 182.5, p-value = 0.9575
## alternative hypothesis: true location shift is not equal to 0
```

```
wilcox.test(Mean_estimate_dilution_considered ~ timepoint, data= h2_data[h2_data$timepoint %in% c("w3", "yHC_unthawed_unspiked"),], na.action = "na.omit", p.adjust.method = "BH")
```

```
## 
##  Wilcoxon rank sum test
## 
## data:  Mean_estimate_dilution_considered by timepoint
## W = 172, p-value = 0.8541
## alternative hypothesis: true location shift is not equal to 0
```

```
wilcox.test(Mean_estimate_dilution_considered ~ timepoint, data= h2_data[h2_data$timepoint %in% c("w4", "yHC_unthawed_unspiked"),], na.action = "na.omit", p.adjust.method = "BH")
```

```
## 
##  Wilcoxon rank sum test
## 
## data:  Mean_estimate_dilution_considered by timepoint
## W = 120, p-value = 0.7413
## alternative hypothesis: true location shift is not equal to 0
```

```
wilcox.test(Mean_estimate_dilution_considered ~ timepoint, data= h2_data[h2_data$timepoint %in% c("w9", "yHC_unthawed_unspiked"),], na.action = "na.omit", p.adjust.method = "BH")
```

```
## 
##  Wilcoxon rank sum test
## 
## data:  Mean_estimate_dilution_considered by timepoint
## W = 138, p-value = 0.4522
## alternative hypothesis: true location shift is not equal to 0
```

```
wilcox.test(Mean_estimate_dilution_considered ~ timepoint, data= h2_data[h2_data$timepoint %in% c("xw13", "yHC_unthawed_unspiked"),], na.action = "na.omit", p.adjust.method = "BH")
```

```
## 
##  Wilcoxon rank sum test
## 
## data:  Mean_estimate_dilution_considered by timepoint
## W = 104, p-value = 0.3022
## alternative hypothesis: true location shift is not equal to 0
```

#### The following code produces the basic Figure S1.

```
layout <- rbind(c(1,1,2), c(3,3,3), c(4,5,6))

grid.arrange(plot.crp, plot.citr, plot.mono, plot.NK, plot.allB, plot.cd4T, layout_matrix = layout)
```
