# Supplementary material for: Specific gut microbiome members are associated with distinct immune markers in pediatric allogeneic hematopoietic stem cell transplantation
Source: Microbiome. 2019 Sep 13;7:131. doi: 10.1186/s40168-019-0745-z (PMC6744702; doi:10.1186/s40168-019-0745-z)
Supplement: Supplementary file 14 — R data analysis report 4. Correlations between immune markers, immune cell counts, and outcomes in HSCT patients. (HTML 1238 kb) [file 40168_2019_745_MOESM14_ESM.html]

Correlations between immune markers, immune cell counts, and outcomes in HSCT patients


# Correlations between immune markers, immune cell counts, and outcomes in HSCT patients

#### *Anna Caecilia Masche*

#### *June 2018*

This is the 4th script used for data analysis and figure generation for the the manuscript by Masche et al. titled “Specific gut microbiome members are associated with distinct immune markers in allogeneic hematopoietic stem cell transplantation”.

This script and associated data are provided by Anna Cäcilia Masche, Susan Holmes, and Sünje Johanna Pamp.

These data and the associated script are licensed under the Creative Commons Attribution-ShareAlike 4.0 International License. To view a copy of this license, visit http://creativecommons.org/licenses/by-sa/4.0/ or send a letter to Creative Commons, PO Box 1866, Mountain View, CA 94042, USA.

Under the condition that appropriate credit is provided, you are free to: 1) Share, copy and redistribute the material in any medium or format 2) Adapt, remix, transform, and build upon the material for any purpose, even commercially.

To see the full license associated with attribution of this work, see the CC-By-CA license, see http://creativecommons.org/licenses/by-sa/4.0/.

The local filename is: Script4\_correlations.Rmd.

## **Install and load packages:**

```
#install.packages("corrplot")
library("corrplot")
library("plyr")
library("dplyr")
```

```
## Warning: package 'dplyr' was built under R version 3.4.1
```

```
## 
## Attaching package: 'dplyr'
```

```
## The following objects are masked from 'package:plyr':
## 
##     arrange, count, desc, failwith, id, mutate, rename, summarise,
##     summarize
```

```
## The following objects are masked from 'package:stats':
## 
##     filter, lag
```

```
## The following objects are masked from 'package:base':
## 
##     intersect, setdiff, setequal, union
```

```
library("Hmisc")
```

```
## Loading required package: lattice
```

```
## Loading required package: survival
```

```
## Loading required package: Formula
```

```
## Loading required package: ggplot2
```

```
## 
## Attaching package: 'Hmisc'
```

```
## The following objects are masked from 'package:dplyr':
## 
##     combine, src, summarize
```

```
## The following objects are masked from 'package:plyr':
## 
##     is.discrete, summarize
```

```
## The following objects are masked from 'package:base':
## 
##     format.pval, round.POSIXt, trunc.POSIXt, units
```

```
library("RColorBrewer")
#install.packages("RcmdrMisc")
library("RcmdrMisc")
```

```
## Loading required package: car
```

```
## 
## Attaching package: 'car'
```

```
## The following object is masked from 'package:dplyr':
## 
##     recode
```

```
## Loading required package: sandwich
```

```
## 
## Attaching package: 'RcmdrMisc'
```

```
## The following object is masked from 'package:Hmisc':
## 
##     Dotplot
```

## **Load table:**

#### The file Data\_matrix\_37\_patients.txt is provided.

```
d_37 <- read.table(file= "M:/Documents/Publications/Masche_R_Scripts_and_Data/Data/Data_matrix_37_patients.txt", sep = "", header = TRUE, row.names = 1)
```

## **To look at the correlations among clinical variables in all 37 patients, subset the variables selected by `adonis` and rename them:**

```
d_37_2 <- subset(d_37, select = c(Rec.age.in.y, CRP_tp_mean, CRP_week1, CRP_week5, CRP_week6, CRP_3months, CRP_6months, hbd2_before, hbd2_week0, hbd2_week1, hbd2_week2, CitDayMinus7, CitDay7, p3p4_dag60, p3p8_dag30, p45p19_dag60, p45p20_dag60, n20p19_dag60, n3p16p56_dag30, n3p16p56_dag60, mean_mono_before, mean_mono_week3, mean_neutro_3months, GVHD_factor))


names(d_37_2)[names(d_37_2) == "hbd2_before"] <- "hBD2_pre"
names(d_37_2)[names(d_37_2) == "hbd2_week0"] <- "hBD2_w0"
names(d_37_2)[names(d_37_2) == "hbd2_week1"] <- "hBD2_w1"
names(d_37_2)[names(d_37_2) == "hbd2_week2"] <- "hBD2_w2"
names(d_37_2)[names(d_37_2) == "mean_mono_before"] <- "mono_pre"
names(d_37_2)[names(d_37_2) == "Rec.age.in.y"] <- "Age"
names(d_37_2)[names(d_37_2) == "n3p16p56_dag30"] <- "NK_m1"
names(d_37_2)[names(d_37_2) == "n3p16p56_dag60"] <- "NK_m2"
names(d_37_2)[names(d_37_2) == "p45p19_dag60"] <- "B_m2"
names(d_37_2)[names(d_37_2) == "p45p20_dag60"] <- "mat_B_m2"
names(d_37_2)[names(d_37_2) == "n20p19_dag60"] <- "immat_B_m2"
names(d_37_2)[names(d_37_2) == "p3p4_dag60"] <- "CD4+_m2"
names(d_37_2)[names(d_37_2) == "p3p8_dag30"] <- "CD8+_m1"
names(d_37_2)[names(d_37_2) == "CitDayMinus7"] <- "Citr_pre"
names(d_37_2)[names(d_37_2) == "CitDay7"] <- "Citr_w1"
names(d_37_2)[names(d_37_2) == "mean_mono_week3"] <- "mono_w3"
names(d_37_2)[names(d_37_2) == "CRP_week1"] <- "CRP_w1"
names(d_37_2)[names(d_37_2) == "CRP_week5"] <- "CRP_w5"
names(d_37_2)[names(d_37_2) == "CRP_week6"] <- "CRP_w6"
names(d_37_2)[names(d_37_2) == "CRP_3months"] <- "CRP_m3"
names(d_37_2)[names(d_37_2) == "CRP_6months"] <- "CRP_m6"
names(d_37_2)[names(d_37_2) == "CRP_tp_mean"] <- "CRP"
names(d_37_2)[names(d_37_2) == "mean_neutro_3months"] <- "neutro_m3"
```

## **Median imputations:**

#### For consistency, we impute the data in the same way as the subset of 30 patients in Script 5.

```
#subset the numeric columns to be imputed
d_37_impute <- d_37_2[sapply(d_37_2, is.numeric)]

#check which columns have less than 20% NAs
d_37_impute_1 <- d_37_impute[which(colMeans(is.na(d_37_impute))<=0.2)]


#Insert additional columns for each column that we will use imputations on, to indicate whether a value was imputed or a real measurement (1/0 as factor)
d_37_impute_1_na <- d_37_impute_1 %>% dplyr::mutate_at(vars(colnames(d_37_impute_1)), funs(ifelse(is.na(.),1,0)))

#rename columns
colnames(d_37_impute_1_na) <- paste0(colnames(d_37_impute_1_na),'_NA')

#convert to factors 
d_37_impute_1_na <- lapply(d_37_impute_1_na, factor)

#impute column median 
for(i in 1:ncol(d_37_impute_1)){d_37_impute_1[is.na(d_37_impute_1[,i]), i] <- median(d_37_impute_1[,i], na.rm = TRUE)}

#add the non-numeric columns again
d_37_impute_all <- cbind(d_37[! sapply(d_37,is.numeric)], d_37_impute_1)

#add the columns indicating where there were NAs
d_37_impute_all <- cbind(d_37_impute_all, d_37_impute_1_na)

#subset the columns without NAs
d_37_impute_all_2 <- d_37_impute_all[,!sapply(d_37_impute_all,function(x) any(is.na(x)))]
```

## **Create a correlation matrix with the `rcorr()` function from package `Hmisc` and plot with the `corrplot` function:**

#### The following code produces the basic Figure 2A.

```
rcorr.clin.vars37<-rcorr(as.matrix(d_37_impute_all_2[, c("hBD2_pre", "hBD2_w0", "hBD2_w1", "hBD2_w2", "mono_pre", "neutro_m3", "CD8+_m1", "Age", "NK_m1", "NK_m2", "B_m2", "mat_B_m2", "immat_B_m2", "CD4+_m2", "Citr_pre", "Citr_w1", "mono_w3", "CRP_w1", "CRP_w5", "CRP_w6", "CRP_m3", "CRP_m6", "CRP")]), type="spearman")

par(xpd=TRUE)

corrplot(rcorr.clin.vars37$r, method="circle", col=rev(brewer.pal(n=9, name = "RdBu")) , tl.col="black", tl.srt=45,          tl.cex=1, cl.cex=1, tl.offset=0.8, cl.pos="n", 
         cl.align.text="r", cl.offset=-1.5, type="lower",
         p.mat= rcorr.clin.vars37$P, sig.level=0.07, insig="n", mar = c(1, 0, 3, 0))


colorlegend(xlim=c(18,25), ylim=c(20,22), rev(brewer.pal(n=9, name = "RdBu")), c(seq(-1,1,.5)), align="l", vertical=FALSE, addlabels=TRUE)
```

#### Calculate adjusted p-values (Holm corrected):

```
rcorr.clin.vars37_adj<-RcmdrMisc::rcorr.adjust(as.matrix(d_37_impute_all_2[, c("hBD2_pre", "hBD2_w0", "hBD2_w1", "hBD2_w2", "mono_pre", "neutro_m3", "CD8+_m1", "Age", "NK_m1", "NK_m2", "B_m2", "mat_B_m2", "immat_B_m2", "CD4+_m2", "Citr_pre", "Citr_w1", "mono_w3", "CRP_w1", "CRP_w5", "CRP_w6", "CRP_m3", "CRP_m6", "CRP")]), type="spearman")

#P-values:
rcorr.clin.vars37_adj$P
```

```
##            hBD2_pre hBD2_w0  hBD2_w1  hBD2_w2  mono_pre neutro_m3 CD8+_m1 
## hBD2_pre   ""       "<.0001" "<.0001" "<.0001" "1.0000" "1.0000"  "1.0000"
## hBD2_w0    "<.0001" ""       "<.0001" "<.0001" "1.0000" "1.0000"  "1.0000"
## hBD2_w1    "<.0001" "<.0001" ""       "<.0001" "1.0000" "1.0000"  "1.0000"
## hBD2_w2    "<.0001" "<.0001" "<.0001" ""       "1.0000" "1.0000"  "1.0000"
## mono_pre   "1.0000" "1.0000" "1.0000" "1.0000" ""       "1.0000"  "1.0000"
## neutro_m3  "1.0000" "1.0000" "1.0000" "1.0000" "1.0000" ""        "1.0000"
## CD8+_m1    "1.0000" "1.0000" "1.0000" "1.0000" "1.0000" "1.0000"  ""      
## Age        "1.0000" "1.0000" "1.0000" "1.0000" "1.0000" "1.0000"  "1.0000"
## NK_m1      "1.0000" "1.0000" "1.0000" "1.0000" "1.0000" "1.0000"  "1.0000"
## NK_m2      "1.0000" "1.0000" "1.0000" "1.0000" "1.0000" "1.0000"  "1.0000"
## B_m2       "1.0000" "1.0000" "1.0000" "1.0000" "1.0000" "1.0000"  "1.0000"
## mat_B_m2   "1.0000" "1.0000" "1.0000" "1.0000" "1.0000" "1.0000"  "1.0000"
## immat_B_m2 "1.0000" "1.0000" "1.0000" "1.0000" "1.0000" "1.0000"  "1.0000"
## CD4+_m2    "1.0000" "1.0000" "1.0000" "1.0000" "1.0000" "1.0000"  "1.0000"
## Citr_pre   "1.0000" "1.0000" "1.0000" "1.0000" "1.0000" "1.0000"  "1.0000"
## Citr_w1    "1.0000" "1.0000" "1.0000" "1.0000" "1.0000" "1.0000"  "1.0000"
## mono_w3    "1.0000" "1.0000" "1.0000" "1.0000" "1.0000" "1.0000"  "1.0000"
## CRP_w1     "1.0000" "1.0000" "1.0000" "1.0000" "1.0000" "1.0000"  "1.0000"
## CRP_w5     "1.0000" "0.7445" "1.0000" "1.0000" "1.0000" "1.0000"  "1.0000"
## CRP_w6     "1.0000" "1.0000" "1.0000" "1.0000" "1.0000" "1.0000"  "1.0000"
## CRP_m3     "1.0000" "1.0000" "1.0000" "1.0000" "1.0000" "0.3601"  "1.0000"
## CRP_m6     "1.0000" "1.0000" "1.0000" "1.0000" "1.0000" "1.0000"  "1.0000"
## CRP        "1.0000" "1.0000" "1.0000" "1.0000" "1.0000" "1.0000"  "1.0000"
##            Age      NK_m1    NK_m2    B_m2     mat_B_m2 immat_B_m2
## hBD2_pre   "1.0000" "1.0000" "1.0000" "1.0000" "1.0000" "1.0000"  
## hBD2_w0    "1.0000" "1.0000" "1.0000" "1.0000" "1.0000" "1.0000"  
## hBD2_w1    "1.0000" "1.0000" "1.0000" "1.0000" "1.0000" "1.0000"  
## hBD2_w2    "1.0000" "1.0000" "1.0000" "1.0000" "1.0000" "1.0000"  
## mono_pre   "1.0000" "1.0000" "1.0000" "1.0000" "1.0000" "1.0000"  
## neutro_m3  "1.0000" "1.0000" "1.0000" "1.0000" "1.0000" "1.0000"  
## CD8+_m1    "1.0000" "1.0000" "1.0000" "1.0000" "1.0000" "1.0000"  
## Age        ""       "1.0000" "1.0000" "1.0000" "1.0000" "1.0000"  
## NK_m1      "1.0000" ""       "1.0000" "0.0046" "0.0114" "1.0000"  
## NK_m2      "1.0000" "1.0000" ""       "1.0000" "1.0000" "1.0000"  
## B_m2       "1.0000" "0.0046" "1.0000" ""       "<.0001" "1.0000"  
## mat_B_m2   "1.0000" "0.0114" "1.0000" "<.0001" ""       "1.0000"  
## immat_B_m2 "1.0000" "1.0000" "1.0000" "1.0000" "1.0000" ""        
## CD4+_m2    "1.0000" "1.0000" "1.0000" "1.0000" "1.0000" "1.0000"  
## Citr_pre   "1.0000" "1.0000" "1.0000" "1.0000" "1.0000" "1.0000"  
## Citr_w1    "1.0000" "1.0000" "1.0000" "1.0000" "1.0000" "1.0000"  
## mono_w3    "1.0000" "1.0000" "1.0000" "0.3745" "0.3105" "1.0000"  
## CRP_w1     "1.0000" "1.0000" "1.0000" "1.0000" "0.8609" "1.0000"  
## CRP_w5     "1.0000" "1.0000" "1.0000" "1.0000" "1.0000" "1.0000"  
## CRP_w6     "1.0000" "1.0000" "1.0000" "1.0000" "1.0000" "1.0000"  
## CRP_m3     "1.0000" "1.0000" "1.0000" "1.0000" "1.0000" "1.0000"  
## CRP_m6     "1.0000" "1.0000" "1.0000" "1.0000" "1.0000" "1.0000"  
## CRP        "1.0000" "1.0000" "1.0000" "1.0000" "1.0000" "1.0000"  
##            CD4+_m2  Citr_pre Citr_w1  mono_w3  CRP_w1   CRP_w5   CRP_w6  
## hBD2_pre   "1.0000" "1.0000" "1.0000" "1.0000" "1.0000" "1.0000" "1.0000"
## hBD2_w0    "1.0000" "1.0000" "1.0000" "1.0000" "1.0000" "0.7445" "1.0000"
## hBD2_w1    "1.0000" "1.0000" "1.0000" "1.0000" "1.0000" "1.0000" "1.0000"
## hBD2_w2    "1.0000" "1.0000" "1.0000" "1.0000" "1.0000" "1.0000" "1.0000"
## mono_pre   "1.0000" "1.0000" "1.0000" "1.0000" "1.0000" "1.0000" "1.0000"
## neutro_m3  "1.0000" "1.0000" "1.0000" "1.0000" "1.0000" "1.0000" "1.0000"
## CD8+_m1    "1.0000" "1.0000" "1.0000" "1.0000" "1.0000" "1.0000" "1.0000"
## Age        "1.0000" "1.0000" "1.0000" "1.0000" "1.0000" "1.0000" "1.0000"
## NK_m1      "1.0000" "1.0000" "1.0000" "1.0000" "1.0000" "1.0000" "1.0000"
## NK_m2      "1.0000" "1.0000" "1.0000" "1.0000" "1.0000" "1.0000" "1.0000"
## B_m2       "1.0000" "1.0000" "1.0000" "0.3745" "1.0000" "1.0000" "1.0000"
## mat_B_m2   "1.0000" "1.0000" "1.0000" "0.3105" "0.8609" "1.0000" "1.0000"
## immat_B_m2 "1.0000" "1.0000" "1.0000" "1.0000" "1.0000" "1.0000" "1.0000"
## CD4+_m2    ""       "1.0000" "1.0000" "1.0000" "1.0000" "1.0000" "1.0000"
## Citr_pre   "1.0000" ""       "1.0000" "1.0000" "1.0000" "1.0000" "1.0000"
## Citr_w1    "1.0000" "1.0000" ""       "1.0000" "1.0000" "1.0000" "1.0000"
## mono_w3    "1.0000" "1.0000" "1.0000" ""       "1.0000" "1.0000" "1.0000"
## CRP_w1     "1.0000" "1.0000" "1.0000" "1.0000" ""       "1.0000" "1.0000"
## CRP_w5     "1.0000" "1.0000" "1.0000" "1.0000" "1.0000" ""       "0.0093"
## CRP_w6     "1.0000" "1.0000" "1.0000" "1.0000" "1.0000" "0.0093" ""      
## CRP_m3     "1.0000" "1.0000" "1.0000" "1.0000" "1.0000" "1.0000" "1.0000"
## CRP_m6     "1.0000" "1.0000" "1.0000" "1.0000" "1.0000" "1.0000" "1.0000"
## CRP        "1.0000" "1.0000" "1.0000" "1.0000" "1.0000" "1.0000" "1.0000"
##            CRP_m3   CRP_m6   CRP     
## hBD2_pre   "1.0000" "1.0000" "1.0000"
## hBD2_w0    "1.0000" "1.0000" "1.0000"
## hBD2_w1    "1.0000" "1.0000" "1.0000"
## hBD2_w2    "1.0000" "1.0000" "1.0000"
## mono_pre   "1.0000" "1.0000" "1.0000"
## neutro_m3  "0.3601" "1.0000" "1.0000"
## CD8+_m1    "1.0000" "1.0000" "1.0000"
## Age        "1.0000" "1.0000" "1.0000"
## NK_m1      "1.0000" "1.0000" "1.0000"
## NK_m2      "1.0000" "1.0000" "1.0000"
## B_m2       "1.0000" "1.0000" "1.0000"
## mat_B_m2   "1.0000" "1.0000" "1.0000"
## immat_B_m2 "1.0000" "1.0000" "1.0000"
## CD4+_m2    "1.0000" "1.0000" "1.0000"
## Citr_pre   "1.0000" "1.0000" "1.0000"
## Citr_w1    "1.0000" "1.0000" "1.0000"
## mono_w3    "1.0000" "1.0000" "1.0000"
## CRP_w1     "1.0000" "1.0000" "1.0000"
## CRP_w5     "1.0000" "1.0000" "1.0000"
## CRP_w6     "1.0000" "1.0000" "1.0000"
## CRP_m3     ""       "0.0670" "0.6445"
## CRP_m6     "0.0670" ""       "0.0189"
## CRP        "0.6445" "0.0189" ""
```

## **Natural killer (NK) and total B cell (mature and immature) reconstitution two months post HSCT with respect to the maximum acute GvHD (aGvHD) grade:**

### GVHD and NK cells in month +2:

#### The following code produces the first part of the basic Figure 2B.

```
ggplot(data= d_37_impute_all_2, aes(y=NK_m2, x=GVHD_factor)) + geom_boxplot()+ geom_point(aes(size=1, color=GVHD_factor), position=position_jitter(width=0.05, height=0), alpha=0.5) +  theme(legend.position="none", panel.border = element_rect(color = "#787878", fill = alpha("white", 0)),panel.grid.minor = element_blank(), panel.grid.major = element_blank(), panel.background = element_blank()) + coord_trans(y = "log10", limy = c(0.05,2)) + scale_y_continuous(breaks= c(0.05, 0.06, 0.07,0.08, 0.1, 0.15, 0.2, 0.25, 0.3, 0.4, 0.5,0.6, 0.8, 1, 1.5, 2), limits=c(0.05,2))
```

### Pairwise Wilcoxon test assessing differences in NK cell count in month +2 in patients with aGvHD grade 0-I vs. grade II-IV

```
wilcox.test(NK_m2 ~ GVHD_factor, data = d_37_impute_all_2)
```

```
## 
##  Wilcoxon rank sum test with continuity correction
## 
## data:  NK_m2 by GVHD_factor
## W = 219.5, p-value = 0.01113
## alternative hypothesis: true location shift is not equal to 0
```

### GVHD and total B cells in month +2:

#### The following code produces the second part of the basic Figure 2B.

```
ggplot(data= d_37_impute_all_2, aes(y=B_m2, x=GVHD_factor)) + geom_boxplot()+ geom_point(aes(size=1, color=GVHD_factor), position=position_jitter(width=0.05, height=0), alpha=0.5) +  theme(legend.position = "none", panel.border = element_rect(color = "#787878", fill = alpha("white", 0)),panel.grid.minor = element_blank(), panel.grid.major = element_blank(), panel.background = element_blank()) + coord_trans(y = "log10", limy = c(0.0006,3.5))+ scale_y_continuous(breaks= c(0.0006, 0.001, 0.005, 0.01, 0.015, 0.05, 0.1, 0.2, 0.25, 0.5, 0.8, 1, 1.5, 2, 3.5), limits=c(0.0006,3.5))
```

### Pairwise Wilcoxon test assessing differences in total B cell count in month +2 in patients with aGvHD grade 0-I vs. grade II-IV

```
wilcox.test(B_m2 ~ GVHD_factor, data = d_37_impute_all_2)
```

```
## 
##  Wilcoxon rank sum test with continuity correction
## 
## data:  B_m2 by GVHD_factor
## W = 254, p-value = 0.0002348
## alternative hypothesis: true location shift is not equal to 0
```
