# Supplementary material for: Specific gut microbiome members are associated with distinct immune markers in pediatric allogeneic hematopoietic stem cell transplantation
Source: Microbiome. 2019 Sep 13;7:131. doi: 10.1186/s40168-019-0745-z (PMC6744702; doi:10.1186/s40168-019-0745-z)
Supplement: Supplementary file 15 — R data analysis report 5. Variable selection and multivariate analyses of immune parameters and intestinal bacterial taxa in HSCT patients. (HTML 2214 kb) [file 40168_2019_745_MOESM15_ESM.html]

Variable selection and multivariate analyses of immune parameters and intestinal bacterial taxa in HSCT patients


# Variable selection and multivariate analyses of immune parameters and intestinal bacterial taxa in HSCT patients

#### *Anna Caecilia Masche*

#### *March 2019*

This is the 5th script used for data analysis and figure generation for the the manuscript by Masche et al. titled “Specific gut microbiome members are associated with distinct immune markers in allogeneic hematopoietic stem cell transplantation”.

This script and associated data are provided by Anna Cäcilia Masche, Susan Holmes, and Sünje Johanna Pamp.

These data and the associated script are licensed under the Creative Commons Attribution-ShareAlike 4.0 International License. To view a copy of this license, visit http://creativecommons.org/licenses/by-sa/4.0/ or send a letter to Creative Commons, PO Box 1866, Mountain View, CA 94042, USA.

Under the condition that appropriate credit is provided, you are free to: 1) Share, copy and redistribute the material in any medium or format 2) Adapt, remix, transform, and build upon the material for any purpose, even commercially.

To see the full license associated with attribution of this work, see the CC-By-CA license, see http://creativecommons.org/licenses/by-sa/4.0/.

The local filename is: Script5\_multivariate.Rmd.

## **Install required packages from CRAN and Bioconductor:**

```
#pkgs_needed = c("devtools", "gridExtra", "plyr", "dplyr", "ggbiplot", "ggord", "DESeq2", "genefilter", "vegan", "mixOmics", "SummarizedExperiment","phyloseq", "permute", "ggrepel")

#letsinstall = setdiff(pkgs_needed, installed.packages(dependencies = TRUE)) 

#if (length(letsinstall) > 0) {
#  source("http://bioconductor.org/biocLite.R")
#  biocLite(letsinstall)
#}
```

## **Load required packages:**

```
library("GenomeInfoDb")
```

```
## Loading required package: BiocGenerics
```

```
## Loading required package: parallel
```

```
## 
## Attaching package: 'BiocGenerics'
```

```
## The following objects are masked from 'package:parallel':
## 
##     clusterApply, clusterApplyLB, clusterCall, clusterEvalQ,
##     clusterExport, clusterMap, parApply, parCapply, parLapply,
##     parLapplyLB, parRapply, parSapply, parSapplyLB
```

```
## The following objects are masked from 'package:stats':
## 
##     IQR, mad, sd, var, xtabs
```

```
## The following objects are masked from 'package:base':
## 
##     anyDuplicated, append, as.data.frame, basename, cbind,
##     colMeans, colnames, colSums, dirname, do.call, duplicated,
##     eval, evalq, Filter, Find, get, grep, grepl, intersect,
##     is.unsorted, lapply, lengths, Map, mapply, match, mget, order,
##     paste, pmax, pmax.int, pmin, pmin.int, Position, rank, rbind,
##     Reduce, rowMeans, rownames, rowSums, sapply, setdiff, sort,
##     table, tapply, union, unique, unsplit, which, which.max,
##     which.min
```

```
## Loading required package: S4Vectors
```

```
## Loading required package: stats4
```

```
## 
## Attaching package: 'S4Vectors'
```

```
## The following object is masked from 'package:base':
## 
##     expand.grid
```

```
## Loading required package: IRanges
```

```
## 
## Attaching package: 'IRanges'
```

```
## The following object is masked from 'package:grDevices':
## 
##     windows
```

```
library("phyloseq")
```

```
## 
## Attaching package: 'phyloseq'
```

```
## The following object is masked from 'package:IRanges':
## 
##     distance
```

```
library("gridExtra")
```

```
## 
## Attaching package: 'gridExtra'
```

```
## The following object is masked from 'package:BiocGenerics':
## 
##     combine
```

```
library("plyr")
```

```
## Warning: package 'plyr' was built under R version 3.5.2
```

```
## 
## Attaching package: 'plyr'
```

```
## The following object is masked from 'package:IRanges':
## 
##     desc
```

```
## The following object is masked from 'package:S4Vectors':
## 
##     rename
```

```
library("dplyr")
```

```
## Warning: package 'dplyr' was built under R version 3.5.2
```

```
## 
## Attaching package: 'dplyr'
```

```
## The following objects are masked from 'package:plyr':
## 
##     arrange, count, desc, failwith, id, mutate, rename, summarise,
##     summarize
```

```
## The following object is masked from 'package:gridExtra':
## 
##     combine
```

```
## The following object is masked from 'package:GenomeInfoDb':
## 
##     intersect
```

```
## The following objects are masked from 'package:IRanges':
## 
##     collapse, desc, intersect, setdiff, slice, union
```

```
## The following objects are masked from 'package:S4Vectors':
## 
##     first, intersect, rename, setdiff, setequal, union
```

```
## The following objects are masked from 'package:BiocGenerics':
## 
##     combine, intersect, setdiff, union
```

```
## The following objects are masked from 'package:stats':
## 
##     filter, lag
```

```
## The following objects are masked from 'package:base':
## 
##     intersect, setdiff, setequal, union
```

```
library("DESeq2")
```

```
## Loading required package: GenomicRanges
```

```
## Loading required package: SummarizedExperiment
```

```
## Loading required package: Biobase
```

```
## Welcome to Bioconductor
## 
##     Vignettes contain introductory material; view with
##     'browseVignettes()'. To cite Bioconductor, see
##     'citation("Biobase")', and for packages 'citation("pkgname")'.
```

```
## 
## Attaching package: 'Biobase'
```

```
## The following object is masked from 'package:phyloseq':
## 
##     sampleNames
```

```
## Loading required package: DelayedArray
```

```
## Loading required package: matrixStats
```

```
## 
## Attaching package: 'matrixStats'
```

```
## The following objects are masked from 'package:Biobase':
## 
##     anyMissing, rowMedians
```

```
## The following object is masked from 'package:dplyr':
## 
##     count
```

```
## The following object is masked from 'package:plyr':
## 
##     count
```

```
## Loading required package: BiocParallel
```

```
## 
## Attaching package: 'DelayedArray'
```

```
## The following objects are masked from 'package:matrixStats':
## 
##     colMaxs, colMins, colRanges, rowMaxs, rowMins, rowRanges
```

```
## The following objects are masked from 'package:base':
## 
##     aperm, apply
```

```
library("genefilter")
```

```
## 
## Attaching package: 'genefilter'
```

```
## The following objects are masked from 'package:matrixStats':
## 
##     rowSds, rowVars
```

```
library("vegan")
```

```
## Loading required package: permute
```

```
## Loading required package: lattice
```

```
## This is vegan 2.5-2
```

```
library("permute")
library("mixOmics")
```

```
## Loading required package: MASS
```

```
## 
## Attaching package: 'MASS'
```

```
## The following object is masked from 'package:genefilter':
## 
##     area
```

```
## The following object is masked from 'package:dplyr':
## 
##     select
```

```
## Loading required package: ggplot2
```

```
## Warning: package 'ggplot2' was built under R version 3.5.2
```

```
## 
## Loaded mixOmics 6.3.2
## 
## Thank you for using mixOmics!
## 
## How to apply our methods: http://www.mixOmics.org for some examples.
## Questions or comments: email us at mixomics[at]math.univ-toulouse.fr  
## Any bugs? https://bitbucket.org/klecao/package-mixomics/issues
## Cite us:  citation('mixOmics')
```

```
library("ggrepel")
library("SummarizedExperiment")
```

## **Load phyloseq object created in Script 1:**

```
phy_obj1_core_cleaned_vsd1 <- readRDS("M:/Documents/Publications/Masche_R_Scripts_and_Data/Data/phy_obj1_core_cleaned_vsd1.Rdata")
```

## **Proceed with preparing the data for the sparse analyses methods**

### First exclude the blank control from the data set

```
NC1 <- grep("negative_control", sample_names(phy_obj1_core_cleaned_vsd1), value=TRUE) #grep extraction control
data <- prune_samples(! sample_names(phy_obj1_core_cleaned_vsd1) %in% NC1, phy_obj1_core_cleaned_vsd1)#subset extraction control
```

### **Which parameters do we want to look at?**

Human beta defensin (hBD)2 and 3  
Intestinal barrier integrity: Citrulline  
Inflammtion: CRP, IL-6  
Immune cell counts/Defensin-expressing cells: lymphocytes, CD3, CD4, CD8, NK, B, neutrophils, monocytes  
Survival, Graft-vs-host disease, occurrence of infections  
Adjust for: Baseline parameters (age, sex, diagnosis (malignant/benign), donor, graft, irradiation), antibiotics

### **Which of these are according to the time point of the sequencing?**

**Continuous variables:**

HBD2 and 3 - “hBD2\_plasma\_pg\_ml\_tp” and “hBD3\_plasma\_pg\_ml\_tp”  
Total lymphocyte count - “Lymphocyte\_count”  
CRP - “CRP..mg.l.”  
(neutrophils - “neutro”) - but mostly not available at day 0 and week 1 and 2  (monocytes - “mono”) - but mostly not available at day 0 and week 1 and 2

**Categorical variables:**

infections - “infect\_occur”  
antibiotics

### **Which variables should be treated as time point independant?**

**Continuous variables:**

age - “Rec.age.in.y”

IL-6 - “pIL6Day7” (only in week 1)  
CD3, CD4, CD8, NK, B cell counts at 1, 2, 3 and 6 months respectively  
CD3: “cd3\_dag30”  
“cd3\_dag60”  
“cd3\_dag90”  
“cd3\_dag180”

CD4: “p3p4\_dag30”  
“p3p4\_dag60”  
“p3p4\_dag90”  
“p3p4\_dag180”

CD8: “p3p8\_dag30”  
“p3p8\_dag60”  
“p3p8\_dag90”  
“p3p8\_dag180”

NK: “n3p16p56\_dag30”  
“n3p16p56\_dag60”  
“n3p16p56\_dag90”  
“n3p16p56\_dag180”

mature B cells:  “p45p20\_dag30”  
“p45p20\_dag60”  
“p45p20\_dag90”  
“p45p20\_dag180”

immature B cells:  “n20p19\_dag30”  
“n20p19\_dag60”  
“n20p19\_dag90”  
“n20p19\_dag180”

all B cells:  “p45p19\_dag30”  
“p45p19\_dag60”  
“p45p19\_dag90”  
“p45p19\_dag180”

neutrophils:  
“mean\_neutro\_before”  
“mean\_neutro\_week3”  
“mean\_neutro\_week4”  
“mean\_neutro\_2months”  
“mean\_neutro\_3months”  
“mean\_neutro\_6months”  
“mean\_neutro\_1year”  
monocytes:  
“mean\_mono\_before”  
“mean\_mono\_week3”  
“mean\_mono\_week4”  
“mean\_mono\_2months”  
“mean\_mono\_3months”  
“mean\_mono\_6months”  
“mean\_mono\_1year”

Citrulline:  
“Citrulline”  
or  
“CitDayMinus7”  “CitDay7”  
“CitDay21”

**Categorical variables:**

Survival - “Alive…1.yes..0.no.”  
GvHD - “GVHD\_factor”  
Graft type - “graft”  
sex - “Sex”  
diagnosis - “malignant\_benign”  
donor type - “Donor”  
Total body irradiation - “Irrad”

## **Calculate the distance matrix based on the OTUs using phyloseq’s `distance()` function (with Manhattan distance)**

```
manhattan_dist <- phyloseq::distance(data, method = "manhattan")
```

## **Convert the sample data of the phyloseq object to a data frame to use it in `adonis`**

```
df <- as(sample_data(data), "data.frame")
```

#### We then explored the categorical patient data by multidimensinal scaling (MDS) with various distance methods within phyloseq’s `distance()` function for a first impression (code and plots not shown).

## **Adonis analysis**

We proceeded with permutational multivariate analysis of variance using distance matrices (adonis) for variable selection. Prior to that, we checked whether the assumption of homogeneous multivariate dispersions for categorical variables is met. We used `betadisper()`, which is a multivariate analogue of the Levene’s test for homogeneity of variances with the null hypothesis of no difference in dispersion between groups (code and plots not shown).

#### **Summary of testing for homogeny of multiple dispersion:**

Variables that that did not meet the assumption (p<0.05): Graft, Sex, Diagnosis  
Variables that that did meet the assumption (p>0.05): Survival, GvHD, Donor  
Eventhough some variables do not meet the assumption of homogeneous multivariate dispersions, we decided to implement adonis for variable selection, but we do not draw our major conclusions from its statistical results.

### **Next, we set up a permutation design for the adonis analysis which respects repeated measurements within patients as well as keeps the chronological order of time points within patients intact**

```
#order df by Patient_ID and timepoint: 
df <- df[order(df$Patient_ID, df$timepoint), ]

### computing the true R2-value

print(fit <- adonis(manhattan_dist ~ timepoint, permutations=1, data = df))
```

```
## 
## Call:
## adonis(formula = manhattan_dist ~ timepoint, data = df, permutations = 1) 
## 
## Permutation: free
## Number of permutations: 1
## 
## Terms added sequentially (first to last)
## 
##           Df SumsOfSqs MeanSqs F.Model      R2 Pr(>F)
## timepoint  6    116436   19406 0.87404 0.05506      1
## Residuals 90   1998243   22203         0.94494       
## Total     96   2114679                 1.00000
```

```
### number of permutations
B <- 1999

### setting up a frame populated by random R2 values:
pop <- rep(NA, B + 1)
  
### first entry will be the true R2:
pop[1] <- fit$aov.tab[1, 5]

### create "permControl" object:
### turn off mirroring as time should only flow in one direction

ctrl <- permute::how(plots = Plots(strata = df$Patient_ID) , within = Within(type = "series", mirror = FALSE))

### Number of observations:
nobs <- nrow(df)

### check permutation (rows represent Patient_ID):
### within each repeated sample (Patient_ID) time points are shuffled with keeping their chronological order intact 
permute::shuffle(nobs, control = ctrl)
```

```
##  [1]  3  4  1  2  5 10  6  7  8  9 11 15 16 12 13 14 17 18 19 20 21 25 26
## [24] 22 23 24 27 28 29 33 34 35 30 31 32 40 36 37 38 39 41 42 43 46 44 45
## [47] 48 49 47 50 51 52 53 54 55 56 57 59 60 61 58 62 63 64 67 65 66 68 69
## [70] 70 71 72 75 73 74 77 78 76 83 79 80 81 82 85 84 89 86 87 88 92 90 91
## [93] 93 94 95 96 97
```

```
### loop:
set.seed(123)
for(i in 2:(B+1)){
     idx <- shuffle(nobs, control = ctrl)
     fit.rand <- adonis(manhattan_dist ~ timepoint[idx], data = df, permutations = 1)
     pop[i] <- fit.rand$aov.tab[1, 5]
}
```

### The customized permutation design (`ctrl`) within patients and with keeping the time point direction can then be applied within adonis.

## **Imputations:**

### **As another preparatory step, median imputation per column is performed for continuous variables that have less than 20% missing values. Variables with more than 20% missing values will not be included in the analysis from this point.**

```
#subset the numeric columns to be imputed
df_impute <- df[sapply(df, is.numeric)]

#check which columns have less than 20% NAs
df_impute_1 <- df_impute[which(colMeans(is.na(df_impute))<=0.2)]

#The following variables have more than 20% NAs and were therefore excluded from here
colnames(df_impute[which(colMeans(is.na(df_impute))>0.2)])
```

```
##  [1] "CRP_5months"           "CRP_7months"          
##  [3] "Citrulline"            "hBD2_plasma_pg_ml"    
##  [5] "hbd2_week4"            "hbd2_3months"         
##  [7] "hBD3_plasma_pg_ml"     "Lymphocyte_count"     
##  [9] "cd3_T_cell_count"      "cd3_dag90"            
## [11] "cd3_dag180"            "cd4_T_cell_count"     
## [13] "p3p4_dag90"            "p3p4_dag180"          
## [15] "cd8_T_cell_count"      "p3p8_dag90"           
## [17] "p3p8_dag180"           "all_B_cell_count"     
## [19] "p45p19_dag90"          "p45p19_dag180"        
## [21] "mature_B_cell_count"   "p45p20_dag90"         
## [23] "p45p20_dag180"         "immature_B_cell_count"
## [25] "n20p19_dag90"          "n20p19_dag180"        
## [27] "NK_cell_count"         "n3p16p56_dag90"       
## [29] "n3p16p56_dag180"       "mono"                 
## [31] "neutro"                "mean_mono_1year"      
## [33] "mean_neutro_1year"
```

```
#Insert additional columns for each column that we will use imputations on to indicate whether a value was imputed or a real measurement (1/0 as factor)

df_impute_1_na <- df_impute_1 %>% dplyr::mutate_at(vars(colnames(df_impute_1[,c(5:ncol(df_impute_1))])), funs(ifelse(is.na(.),1,0)))

#rename columns: 

colnames(df_impute_1_na)[5:ncol(df_impute_1_na)] <- paste0(colnames(df_impute_1_na)[5:ncol(df_impute_1_na)],'_NA')

#convert to fators: 
df_impute_1_na[,5:ncol(df_impute_1_na)] <- lapply(df_impute_1_na[,5:ncol(df_impute_1_na)], factor)

#impute column median 
for(i in 5:ncol(df_impute_1)){df_impute_1[is.na(df_impute_1[,i]), i] <- median(df_impute_1[,i], na.rm = TRUE)}

#add back the non-numeric columns
df_impute_all <- cbind(df[! sapply(df,is.numeric)], df_impute_1)

#add the columns indicating where there were NAs: 
df_impute_all <- cbind(df_impute_all, df_impute_1_na[5:ncol(df_impute_1_na)])

#subset the columns without NAs
df_impute_all_2 <- df_impute_all[,!sapply(df_impute_all,function(x) any(is.na(x)))]

#order again (so permutation design fits)
df_impute_all_2 <- df_impute_all_2[order(df_impute_all_2$Patient_ID, df_impute_all_2$timepoint), ]
```

## **ADONIS**

#### Adonis analysis is performed with those variables that were measured at simultaneous time points with the microbiome data. Those are predominantly human beta defensin 2 secretion levels (hbd2), C-reactive protein levels (CRP) and the total lymphocyte counts. The permutation design defined above which permutes only within patients and respects the order of time points will be used.

Due to the permutation design, including time-independent variables (e.g. patient’s sex) is meaningless as they do not change over time within a patient. So at this point of the analysis, we cannot adjust for the time-independent patient baseline parameters (age, sex, donor type, malignant vs. benign diagnosis, aGvHD grade, irradiation therapy, graft type). They will be included in the ordination further down again. But we have antibiotics application data for all time points, which are included here.

We are using `adonis2()`, because it allows looking for marginal effects of each variable and does not depend on in which order the variables are provided.

```
set.seed(123)  #set seed for reproducibility 

res_adonis2 <- adonis2(manhattan_dist ~  
hBD2_plasma_pg_ml_tp
+ CRP_tp_mean
+ Lymphocyte_count_tp
+  Sulfamethoxazole_Trimethoprim_tp              
+  Ceftazidim_tp                                 
+  Meropenem_tp                                  
+  Sulfamethizole_tp                             
+  Chloramphenicol_tp                            
+  Vancomycin_tp
+  Cefuroxime_tp                                  
+  Hexamycin_tp                                  
+  Ciprofloxacin_tp                              
+  Piperacillin_tp                               
+  Colistin_tp                                   
+  Linezolid_tp                                  
+  Metronidazol_tp                               
+  Teicoplanin_tp                                
+  Amoxicillin_tp                                
+  Fusidic_acid_tp                               
+  Dicloxacillin_tp
 , data = df_impute_all_2,  by = "margin", permutations = ctrl) 

res_adonis2
```

```
## Permutation test for adonis under reduced model
## Marginal effects of terms
## Plots: df$Patient_ID, plot permutation: none
## Permutation: series
## Number of permutations: 199
## 
## adonis2(formula = manhattan_dist ~ hBD2_plasma_pg_ml_tp + CRP_tp_mean + Lymphocyte_count_tp + Sulfamethoxazole_Trimethoprim_tp + Ceftazidim_tp + Meropenem_tp + Sulfamethizole_tp + Chloramphenicol_tp + Vancomycin_tp + Cefuroxime_tp + Hexamycin_tp + Ciprofloxacin_tp + Piperacillin_tp + Colistin_tp + Linezolid_tp + Metronidazol_tp + Teicoplanin_tp + Amoxicillin_tp + Fusidic_acid_tp + Dicloxacillin_tp, data = df_impute_all_2, permutations = ctrl, by = "margin")
##                                  Df SumOfSqs      R2      F Pr(>F)  
## hBD2_plasma_pg_ml_tp              1     9763 0.00462 0.4635  0.915  
## CRP_tp_mean                       1    28707 0.01358 1.3629  0.015 *
## Lymphocyte_count_tp               1    21541 0.01019 1.0227  0.610  
## Sulfamethoxazole_Trimethoprim_tp  1    21039 0.00995 0.9988  0.285  
## Ceftazidim_tp                     1    32731 0.01548 1.5539  0.060 .
## Meropenem_tp                      1    13007 0.00615 0.6175  0.585  
## Sulfamethizole_tp                 1    10975 0.00519 0.5211  0.775  
## Chloramphenicol_tp                1     7053 0.00334 0.3349  0.730  
## Vancomycin_tp                     1    35771 0.01692 1.6982  0.055 .
## Cefuroxime_tp                     1    11572 0.00547 0.5494  0.570  
## Hexamycin_tp                      1    19489 0.00922 0.9252  0.425  
## Ciprofloxacin_tp                  1    32415 0.01533 1.5389  0.205  
## Piperacillin_tp                   1     6404 0.00303 0.3040  0.740  
## Colistin_tp                       1    25159 0.01190 1.1944  0.355  
## Linezolid_tp                      1     6580 0.00311 0.3124  0.395  
## Metronidazol_tp                   0        0 0.00000   -Inf         
## Teicoplanin_tp                    0        0 0.00000   -Inf         
## Amoxicillin_tp                    1     4792 0.00227 0.2275  0.795  
## Fusidic_acid_tp                   1    52879 0.02501 2.5105  0.030 *
## Dicloxacillin_tp                  0        0 0.00000   -Inf         
## Residual                         77  1621897 0.76697                
## Total                            96  2114679 1.00000                
## ---
## Signif. codes:  0 '***' 0.001 '**' 0.01 '*' 0.05 '.' 0.1 ' ' 1
```

#### The results of the `adonis2()` analysis indicate that the C-reactive protein level (at simultaneaous time points) as well as the application of specific antibiotics at simultaneous time points could be influencing compositional differences in the microbiome between samples. Therfore, these variables should be included in downstream analyses.

### In the following, we run another `adonis2()` analysis with the time point independent variables with permutations across time points, but within patients. We still have repeated measurements, but the order of time points doesn’t matter here, because values are the same for all time points within a patient.

#### Here we can also include and adjust for the patient baseline parameters (age, sex, donor type, malignant vs. benign diagnosis, aGvHD grade, Irradiation therapy, graft type), and include survival and relapse rate.

### We run `adonis2()` for the previously tested biomarker hbd2, now in wide format (i.e. variables: hbd2\_before, hbd2\_week1, hbd2\_week2 etc.), thereby achieving that also later time points that are not represented in the microbiome data, can be taken into consideration. Furthermore, this way not only simultaneous effects are tested.

```
set.seed(123)

res_adonis2_1 <- adonis2(manhattan_dist ~  
Sex
+ Donor
+ Rec.age.in.y
+ Irrad
+ GVHD_factor  
+ malignant_benign                      
+ graft                      
+ Sens.0.relapse.1.death.Now. 
+ Alive...1.yes..0.no.
+ hbd2_before                           
+ hbd2_week0                            
+ hbd2_week1                            
+ hbd2_week2                            
+ hbd2_week3
+ hbd2_2months
, data = df_impute_all_2, strata = Patient_ID, by = "margin") 

res_adonis2_1
```

```
## Permutation test for adonis under reduced model
## Marginal effects of terms
## Permutation: free
## Number of permutations: 999
## 
## adonis2(formula = manhattan_dist ~ Sex + Donor + Rec.age.in.y + Irrad + GVHD_factor + malignant_benign + graft + Sens.0.relapse.1.death.Now. + Alive...1.yes..0.no. + hbd2_before + hbd2_week0 + hbd2_week1 + hbd2_week2 + hbd2_week3 + hbd2_2months, data = df_impute_all_2, by = "margin", strata = Patient_ID)
##                             Df SumOfSqs      R2      F Pr(>F)    
## Sex                          1    17686 0.00836 0.8527  0.571    
## Donor                        1    26296 0.01243 1.2678  0.220    
## Rec.age.in.y                 1    40830 0.01931 1.9686  0.042 *  
## Irrad                        1    32453 0.01535 1.5647  0.105    
## GVHD_factor                  1    26070 0.01233 1.2570  0.233    
## malignant_benign             1    16498 0.00780 0.7955  0.667    
## graft                        3   128217 0.06063 2.0607  0.030 *  
## Sens.0.relapse.1.death.Now.  1    27516 0.01301 1.3267  0.171    
## Alive...1.yes..0.no.         1    27818 0.01315 1.3412  0.184    
## hbd2_before                  1    89958 0.04254 4.3373  0.001 ***
## hbd2_week0                   1    39432 0.01865 1.9012  0.037 *  
## hbd2_week1                   1    62425 0.02952 3.0098  0.007 ** 
## hbd2_week2                   1    70010 0.03311 3.3755  0.004 ** 
## hbd2_week3                   1    32106 0.01518 1.5480  0.088 .  
## hbd2_2months                 1    33806 0.01599 1.6300  0.104    
## Residual                    79  1638486 0.77482                  
## Total                       96  2114679 1.00000                  
## ---
## Signif. codes:  0 '***' 0.001 '**' 0.01 '*' 0.05 '.' 0.1 ' ' 1
```

#### The results of the `adonis2` analysis indicate that even though the secretion levels of hBD2 might not have an effect at simultaneous time points, the hBD2 secretion before transplantation, at the time of transplantation and in week 1 and 2 might be associated with microbial compostion differences.

Furthermore, the age of the patients at the time of transplantation should be taken into consideration and adjusted for.

### We run `adonis2()` for the previously tested biomarker CRP, now in wide format.

```
set.seed(123)

res_adonis2_2 <- adonis2(manhattan_dist ~  
Sex
+ Donor
+ Rec.age.in.y
+ Irrad
+ GVHD_factor  
+ malignant_benign                      
+ graft                      
+ Sens.0.relapse.1.death.Now. 
+ Alive...1.yes..0.no.
+ CRP_before
+ CRP_week0
+ CRP_week1
+ CRP_week2
+ CRP_week3
+ CRP_week4
+ CRP_week5
+ CRP_week6
+ CRP_2months                           
+ CRP_3months                          
+ CRP_4months                          
+ CRP_6months 
, data = df_impute_all_2, strata = Patient_ID, by = "margin") 

res_adonis2_2
```

```
## Permutation test for adonis under reduced model
## Marginal effects of terms
## Permutation: free
## Number of permutations: 999
## 
## adonis2(formula = manhattan_dist ~ Sex + Donor + Rec.age.in.y + Irrad + GVHD_factor + malignant_benign + graft + Sens.0.relapse.1.death.Now. + Alive...1.yes..0.no. + CRP_before + CRP_week0 + CRP_week1 + CRP_week2 + CRP_week3 + CRP_week4 + CRP_week5 + CRP_week6 + CRP_2months + CRP_3months + CRP_4months + CRP_6months, data = df_impute_all_2, by = "margin", strata = Patient_ID)
##                             Df SumOfSqs      R2      F Pr(>F)   
## Sex                          1    29987 0.01418 1.5461  0.135   
## Donor                        1    33051 0.01563 1.7041  0.070 . 
## Rec.age.in.y                 1    20521 0.00970 1.0580  0.362   
## Irrad                        1    10642 0.00503 0.5487  0.899   
## GVHD_factor                  1    48930 0.02314 2.5228  0.006 **
## malignant_benign             1    32930 0.01557 1.6978  0.087 . 
## graft                        3   117506 0.05557 2.0195  0.031 * 
## Sens.0.relapse.1.death.Now.  1    21813 0.01032 1.1247  0.286   
## Alive...1.yes..0.no.         1    39776 0.01881 2.0509  0.034 * 
## CRP_before                   1    15714 0.00743 0.8102  0.631   
## CRP_week0                    1    22573 0.01067 1.1639  0.280   
## CRP_week1                    1    51055 0.02414 2.6324  0.011 * 
## CRP_week2                    1    34122 0.01614 1.7593  0.098 . 
## CRP_week3                    1    41269 0.01952 2.1278  0.055 . 
## CRP_week4                    1    27106 0.01282 1.3976  0.169   
## CRP_week5                    1    41965 0.01984 2.1637  0.026 * 
## CRP_week6                    1    38860 0.01838 2.0036  0.029 * 
## CRP_2months                  1    31527 0.01491 1.6255  0.095 . 
## CRP_3months                  1    39972 0.01890 2.0610  0.034 * 
## CRP_4months                  1    23961 0.01133 1.2354  0.252   
## CRP_6months                  1    39945 0.01889 2.0596  0.024 * 
## Residual                    73  1415828 0.66952                 
## Total                       96  2114679 1.00000                 
## ---
## Signif. codes:  0 '***' 0.001 '**' 0.01 '*' 0.05 '.' 0.1 ' ' 1
```

#### The results of the `adonis2()` analysis indicate that the level of C-ractive protein seems to be especially important in week 1, 5 and 6 and also at the 3 and 6 months follow up time point. Furthermore, GvHD grade, survival and graft type should be taken into consideration and/or adjusted for in further analyses.

### Next, we run an `adonis2()` analysis on citrulline levels before, and in week +1 and +3 after HSCT as well as Interleukin 6 (pIL6Day7) in week +1 after HSCT. These were only measured at a subset of the time points and can therefore only be tested in this way (wide format data). Again baseline parameters as well as survival and relapse outcomes will be adjusted for.

```
set.seed(123)

res_adonis2_3 <- adonis2(manhattan_dist ~  
Sex
+ Donor
+ Rec.age.in.y
+ Irrad
+ GVHD_factor  
+ malignant_benign                      
+ graft                      
+ Sens.0.relapse.1.death.Now. 
+ Alive...1.yes..0.no.
+ pIL6Day7                              
+ CitDayMinus7                         
+ CitDay7                             
+ CitDay21 
, data = df_impute_all_2, strata = Patient_ID, by = "margin") 

res_adonis2_3
```

```
## Permutation test for adonis under reduced model
## Marginal effects of terms
## Permutation: free
## Number of permutations: 999
## 
## adonis2(formula = manhattan_dist ~ Sex + Donor + Rec.age.in.y + Irrad + GVHD_factor + malignant_benign + graft + Sens.0.relapse.1.death.Now. + Alive...1.yes..0.no. + pIL6Day7 + CitDayMinus7 + CitDay7 + CitDay21, data = df_impute_all_2, by = "margin", strata = Patient_ID)
##                             Df SumOfSqs      R2      F Pr(>F)   
## Sex                          1    32496 0.01537 1.6026  0.098 . 
## Donor                        1    19274 0.00911 0.9505  0.476   
## Rec.age.in.y                 1    26174 0.01238 1.2908  0.199   
## Irrad                        1    23475 0.01110 1.1577  0.300   
## GVHD_factor                  1    13339 0.00631 0.6579  0.803   
## malignant_benign             1    17116 0.00809 0.8441  0.599   
## graft                        3    62410 0.02951 1.0260  0.372   
## Sens.0.relapse.1.death.Now.  1    31370 0.01483 1.5471  0.096 . 
## Alive...1.yes..0.no.         1    41651 0.01970 2.0541  0.030 * 
## pIL6Day7                     1    23180 0.01096 1.1431  0.311   
## CitDayMinus7                 1    78105 0.03693 3.8519  0.002 **
## CitDay7                      1    42029 0.01987 2.0727  0.021 * 
## CitDay21                     1    33872 0.01602 1.6705  0.069 . 
## Residual                    81  1642450 0.77669                 
## Total                       96  2114679 1.00000                 
## ---
## Signif. codes:  0 '***' 0.001 '**' 0.01 '*' 0.05 '.' 0.1 ' ' 1
```

#### Citrulline levels before, and also in week +1 seem to play a role in composition state differences. Again, survival should also be taken into consideration.

### Next, we run an `adonis2()` analysis on late follow up time points of the total lymphocyte count and subcategories of lymphocytes: CD3+ (cd3), CD4+ (p3p4), CD8+ (p3p8), B (immature: n20p19, mature: p45p20, all: p45p19) and NK (n3p16p56) cell counts at months +1 and +2 after HSCT which represent immune reconstitution. Again baseline parameters as well as survival and relapse outcomes will be adjusted for.

```
set.seed(123)

res_adonis2_4 <- adonis2(manhattan_dist ~  Sex
+ Donor
+ Rec.age.in.y
+ Irrad                  
+ GVHD_factor  
+ malignant_benign                      
+ graft 
+ Sens.0.relapse.1.death.Now. 
+ Alive...1.yes..0.no.
+ cd3_dag30                             
+ cd3_dag60                             
+ p3p4_dag30                            
+ p3p4_dag60                            
+ p3p8_dag30                            
+ p3p8_dag60                            
+ p45p19_dag30                          
+ p45p19_dag60                          
+ p45p20_dag30                          
+ p45p20_dag60                          
+ n20p19_dag30                          
+ n20p19_dag60                          
+ n3p16p56_dag30                        
+ n3p16p56_dag60
+ Lymfodag60                            
+ Lymfodag90                            
+ Lymfodag180                           
+ Lymfodag360
, data = df_impute_all_2, strata = Patient_ID, by = "margin") 
 
res_adonis2_4
```

```
## Permutation test for adonis under reduced model
## Marginal effects of terms
## Permutation: free
## Number of permutations: 999
## 
## adonis2(formula = manhattan_dist ~ Sex + Donor + Rec.age.in.y + Irrad + GVHD_factor + malignant_benign + graft + Sens.0.relapse.1.death.Now. + Alive...1.yes..0.no. + cd3_dag30 + cd3_dag60 + p3p4_dag30 + p3p4_dag60 + p3p8_dag30 + p3p8_dag60 + p45p19_dag30 + p45p19_dag60 + p45p20_dag30 + p45p20_dag60 + n20p19_dag30 + n20p19_dag60 + n3p16p56_dag30 + n3p16p56_dag60 + Lymfodag60 + Lymfodag90 + Lymfodag180 + Lymfodag360, data = df_impute_all_2, by = "margin", strata = Patient_ID)
##                             Df SumOfSqs      R2      F Pr(>F)   
## Sex                          1    67605 0.03197 3.4211  0.003 **
## Donor                        1    35655 0.01686 1.8043  0.046 * 
## Rec.age.in.y                 1    28037 0.01326 1.4188  0.123   
## Irrad                        1    21791 0.01030 1.1027  0.309   
## GVHD_factor                  1    32095 0.01518 1.6241  0.074 . 
## malignant_benign             1    31078 0.01470 1.5727  0.089 . 
## graft                        3   112291 0.05310 1.8941  0.006 **
## Sens.0.relapse.1.death.Now.  1    30206 0.01428 1.5285  0.094 . 
## Alive...1.yes..0.no.         1    31372 0.01484 1.5875  0.079 . 
## cd3_dag30                    1    28654 0.01355 1.4500  0.123   
## cd3_dag60                    1    17891 0.00846 0.9054  0.501   
## p3p4_dag30                   1    30266 0.01431 1.5316  0.090 . 
## p3p4_dag60                   1    38132 0.01803 1.9296  0.037 * 
## p3p8_dag30                   1    38615 0.01826 1.9541  0.029 * 
## p3p8_dag60                   1    21275 0.01006 1.0766  0.337   
## p45p19_dag30                 1    26588 0.01257 1.3454  0.166   
## p45p19_dag60                 1    41803 0.01977 2.1154  0.025 * 
## p45p20_dag30                 1    26622 0.01259 1.3472  0.167   
## p45p20_dag60                 1    41697 0.01972 2.1100  0.027 * 
## n20p19_dag30                 1    28078 0.01328 1.4208  0.133   
## n20p19_dag60                 1    40142 0.01898 2.0313  0.029 * 
## n3p16p56_dag30               1    63891 0.03021 3.2331  0.002 **
## n3p16p56_dag60               1    44566 0.02107 2.2552  0.020 * 
## Lymfodag60                   1    22196 0.01050 1.1232  0.324   
## Lymfodag90                   1    24450 0.01156 1.2373  0.224   
## Lymfodag180                  1    26278 0.01243 1.3298  0.177   
## Lymfodag360                  1    29658 0.01402 1.5008  0.114   
## Residual                    67  1324017 0.62611                 
## Total                       96  2114679 1.00000                 
## ---
## Signif. codes:  0 '***' 0.001 '**' 0.01 '*' 0.05 '.' 0.1 ' ' 1
```

#### Concerning immune reconstitution, CD4+ T cells as well as mature, immature and total B cell counts in month +2, NK cells counts in months +1 and +2, and CD8+ T cells in month +1 might be associated with microbial composition differences. Also sex, donor type, and graft type, show potential associations in this context.

## Next, we run an `adonis2()` analysis on monocyte and neutrophil counts which were only measured at a subset of time points. Again baseline parameters as well as survival and relapse outcomes will be adjusted for.

```
set.seed(123)                

res_adonis2_5 <- adonis2(manhattan_dist ~  
Sex
+ Donor
+ Rec.age.in.y
+ Irrad                  
+ GVHD_factor  
+ malignant_benign                      
+ graft 
+ Sens.0.relapse.1.death.Now. 
+ Alive...1.yes..0.no.
+ mean_mono_before
+ mean_mono_week3                       
+ mean_mono_week4 
+ mean_mono_2months                     
+ mean_mono_3months                     
+ mean_mono_6months
+ mean_neutro_before                    
+ mean_neutro_week3                     
+ mean_neutro_week4  
+ mean_neutro_2months                   
+ mean_neutro_3months                   
+ mean_neutro_6months 
, data = df_impute_all_2, strata = Patient_ID, by = "margin") 
 
res_adonis2_5
```

```
## Permutation test for adonis under reduced model
## Marginal effects of terms
## Permutation: free
## Number of permutations: 999
## 
## adonis2(formula = manhattan_dist ~ Sex + Donor + Rec.age.in.y + Irrad + GVHD_factor + malignant_benign + graft + Sens.0.relapse.1.death.Now. + Alive...1.yes..0.no. + mean_mono_before + mean_mono_week3 + mean_mono_week4 + mean_mono_2months + mean_mono_3months + mean_mono_6months + mean_neutro_before + mean_neutro_week3 + mean_neutro_week4 + mean_neutro_2months + mean_neutro_3months + mean_neutro_6months, data = df_impute_all_2, by = "margin", strata = Patient_ID)
##                             Df SumOfSqs      R2      F Pr(>F)   
## Sex                          1    52423 0.02479 2.5585  0.015 * 
## Donor                        1    25234 0.01193 1.2315  0.249   
## Rec.age.in.y                 1    33524 0.01585 1.6361  0.091 . 
## Irrad                        1    14699 0.00695 0.7174  0.726   
## GVHD_factor                  1    12479 0.00590 0.6090  0.853   
## malignant_benign             1    14011 0.00663 0.6838  0.774   
## graft                        3   103841 0.04910 1.6893  0.065 . 
## Sens.0.relapse.1.death.Now.  1    29597 0.01400 1.4444  0.136   
## Alive...1.yes..0.no.         1    15791 0.00747 0.7707  0.697   
## mean_mono_before             1    47349 0.02239 2.3109  0.013 * 
## mean_mono_week3              1    64242 0.03038 3.1353  0.002 **
## mean_mono_week4              1    17924 0.00848 0.8748  0.561   
## mean_mono_2months            1    25930 0.01226 1.2655  0.187   
## mean_mono_3months            1    18269 0.00864 0.8916  0.516   
## mean_mono_6months            1    15794 0.00747 0.7708  0.654   
## mean_neutro_before           1    25198 0.01192 1.2298  0.225   
## mean_neutro_week3            1    31161 0.01474 1.5208  0.149   
## mean_neutro_week4            1    29036 0.01373 1.4171  0.137   
## mean_neutro_2months          1    16656 0.00788 0.8129  0.611   
## mean_neutro_3months          1    48466 0.02292 2.3653  0.010 **
## mean_neutro_6months          1    15960 0.00755 0.7789  0.653   
## Residual                    73  1495766 0.70732                 
## Total                       96  2114679 1.00000                 
## ---
## Signif. codes:  0 '***' 0.001 '**' 0.01 '*' 0.05 '.' 0.1 ' ' 1
```

#### Regarding monocyte and neutrophil counts, recipient derived monocyte counts before transplantation, donor derived monocyte counts in week +3, and donor derived neutrophil counts in month +3 might have an impact on microbial composition differences. And sex should be adjusted for.

### **Next, we prepare the data for follow up analysis steps:**

#### **We make a new phyloseq object that contains the imputed data as sample data**

```
samdata <- sample_data(df_impute_all_2)
physeq2 = merge_phyloseq(samdata, otu_table(data), tax_table(data))
```

## **Sparse partial least squares (sPLS) regression analysis with the variables identified in adonis, using the `mixOmics` package**

### **Define X (`otu_table`) and Y (`sample_data`). For Y, subset the `sample_data` only for those variables previously chosen in adonis**

```
physeq2_sample_data_sub <- get_variable(physeq2, c("Rec.age.in.y", "CRP_tp_mean", "CRP_week1", "CRP_week5", "CRP_week6", "CRP_3months", "CRP_6months", "hbd2_before", "hbd2_week0", "hbd2_week1", "hbd2_week2", "CitDayMinus7", "CitDay7", "p3p4_dag60", "p3p8_dag30", "p45p19_dag60", "p45p20_dag60", "n20p19_dag60", "n3p16p56_dag30", "n3p16p56_dag60", "mean_mono_before", "mean_mono_week3", "mean_neutro_3months"))


X <- as.data.frame(t(otu_table(physeq2)))
Y <- physeq2_sample_data_sub
```

### **Check that the subjects are matched in the two data sets**

```
head(cbind(rownames(X), rownames(Y)))
```

```
##      [,1]        [,2]       
## [1,] "P10.w2"    "P10.w2"   
## [2,] "P10.w1"    "P10.w1"   
## [3,] "P30.pre.a" "P30.pre.a"
## [4,] "P15.d0"    "P15.d0"   
## [5,] "P20.w3"    "P20.w3"   
## [6,] "P07.w1"    "P07.w1"
```

```
all(rownames(X)==rownames(Y))
```

```
## [1] TRUE
```

### **Pre-assess the data with a PCA**

### PCA on the OTU count data (X)

```
pca.otu <- mixOmics::pca(X, ncomp = 10, center = TRUE, scale = TRUE)

pca.otu
```

```
## Eigenvalues for the first 10 principal components, see object$sdev^2: 
##       PC1       PC2       PC3       PC4       PC5       PC6       PC7 
## 38.383688 17.021327 13.570016 11.229052  9.714034  9.099097  8.180394 
##       PC8       PC9      PC10 
##  8.004250  6.649793  6.071807 
## 
## Proportion of explained variance for the first 10 principal components, see object$explained_variance: 
##        PC1        PC2        PC3        PC4        PC5        PC6 
## 0.16060121 0.07121894 0.05677831 0.04698348 0.04064449 0.03807154 
##        PC7        PC8        PC9       PC10 
## 0.03422759 0.03349059 0.02782340 0.02540505 
## 
## Cumulative proportion explained variance for the first 10 principal components, see object$cum.var: 
##       PC1       PC2       PC3       PC4       PC5       PC6       PC7 
## 0.1606012 0.2318201 0.2885985 0.3355819 0.3762264 0.4142980 0.4485256 
##       PC8       PC9      PC10 
## 0.4820161 0.5098395 0.5352446 
## 
##  Other available components: 
##  -------------------- 
##  loading vectors: see object$rotation
```

```
plot(pca.otu)
```

It seems that most variance is already explained by the 1st PC.

### PCA on the clinical data (Y)

```
pca.clinical <- mixOmics::pca(Y, ncomp = 10, center = TRUE, scale = TRUE)

pca.clinical
```

```
## Eigenvalues for the first 10 principal components, see object$sdev^2: 
##       PC1       PC2       PC3       PC4       PC5       PC6       PC7 
## 4.2991999 4.1628837 2.8571837 2.4645516 1.6414720 1.4169169 1.0718814 
##       PC8       PC9      PC10 
## 1.0278647 0.8772147 0.7343746 
## 
## Proportion of explained variance for the first 10 principal components, see object$explained_variance: 
##        PC1        PC2        PC3        PC4        PC5        PC6 
## 0.18692174 0.18099494 0.12422538 0.10715442 0.07136835 0.06160508 
##        PC7        PC8        PC9       PC10 
## 0.04660354 0.04468977 0.03813977 0.03192933 
## 
## Cumulative proportion explained variance for the first 10 principal components, see object$cum.var: 
##       PC1       PC2       PC3       PC4       PC5       PC6       PC7 
## 0.1869217 0.3679167 0.4921421 0.5992965 0.6706648 0.7322699 0.7788734 
##       PC8       PC9      PC10 
## 0.8235632 0.8617030 0.8936323 
## 
##  Other available components: 
##  -------------------- 
##  loading vectors: see object$rotation
```

```
plot(pca.clinical)
```

It seems that the most variance is explained by the first 2 PCs.

### **Choosing the number of components `ncomp` for sparse PLS regression:**

#### We set `keepY` (the number of variables for the model to keep on each component) to 23, so that all (continuous) variables are kept (because it is already a preselected subset). We set `keepX` (the number of OTUs to keep on each component) to 30.

#### We further used the `perf()` function within package `mixomics` to tune the number of components to use, but received values below the inclusion threshold of 0.0975 (Code and plots not shown). Therefore the scree plots above were more insightful, suggesting the inclusion of 2 components.

### **Run the sPLS:**

```
my.spls <- spls(X, Y, ncomp =2, keepX = c(30,30), keepY= c(23,23), mode = "regression")
```

### **Plot the first 2 components**

#### Make nicer variable labels

```
my.spls$names$colnames$Y <- c("Age", "CRP", "CRP_w1", "CRP_w5", "CRP_w6", "CRP_m3", "CRP_m6", "hBD2_pre", "hBD2_w0", "hBD2_w1", "hBD2_w2", "Citr_pre", "Citr_w1", "CD4+_m2", "CD8+_m1", "B_m2", "mat_B_m2", "immat_B_m2", "NK_m1", "NK_m2","mono_pre", "mono_w3", "neutro_m3")

colnames(my.spls$Y) <- c("Age", "CRP", "CRP_w1", "CRP_w5", "CRP_w6", "CRP_m3", "CRP_m6", "hBD2_pre", "hBD2_w0", "hBD2_w1", "hBD2_w2", "Citr_pre", "Citr_w1", "CD4+_m2", "CD8+_m1", "B_m2", "mat_B_m2", "immat_B_m2", "NK_m1", "NK_m2","mono_pre", "mono_w3", "neutro_m3")

rownames(my.spls$loadings$Y) <- c("Age", "CRP", "CRP_w1", "CRP_w5", "CRP_w6", "CRP_m3", "CRP_m6", "hBD2_pre", "hBD2_w0", "hBD2_w1", "hBD2_w2", "Citr_pre", "Citr_w1", "CD4+_m2", "CD8+_m1", "B_m2", "mat_B_m2", "immat_B_m2", "NK_m1", "NK_m2","mono_pre", "mono_w3", "neutro_m3")
```

#### **First plot correlation circle plots for OTUs and variables seperately**

```
#dev.off()
plotVar(my.spls, comp =c(1,2), overlap = FALSE, cex = c(3,3), cutoff = 0.2, style = "ggplot2", var.names = TRUE, col=c("red","black"))
```

### **Now plot boths OTUs and variables together (component 1 and 2)**

#### The following code produces the basic Figure 3A.

```
#dev.off()
plotVar(my.spls, comp =c(1,2), overlap = TRUE, cex = c(3,5), cutoff = 0.2, style = "ggplot2", var.names = c(FALSE,TRUE), col=c("red","black"))
```

On first glance, there seem to be 3 clusters of groups of variables and OTUs associated with each other.

### **Clustered Image Map**

#### Including components 1 and 2.

#### Specify clustering (“complete” for complete linkage) and distance method (“correlation” for Pearson’s correlation)

#### The following code produces the basic Figure 3B.

##### When running the script, if the following error appears: `Error in plot.new() : figure margins too large`, plot in console instead and zoom, then the plot will appear.

```
cim_my.spls <- mixOmics::cim(my.spls, comp = 1:2, xlab = "clinical variables", ylab = "OTUs", margins = c(7,20), dist.method = c("correlation", "correlation"), clust.method=c("complete", "complete"), mapping = "XY")
```

### **Extract correlation coefficients from `cim` and add a column for “cluster”:**

```
clusters <- cim_my.spls$mat

cluster <- c(rep("2",21), rep("1",14), rep("3",22))

clusters <- cbind(clusters, cluster)

clusters_otus_df <- data.frame(cbind(rownames(clusters), clusters[,"cluster"]))

names(clusters_otus_df)[names(clusters_otus_df) == 'X1'] <- 'seq'
names(clusters_otus_df)[names(clusters_otus_df) == 'X2'] <- 'cluster'

#get taxonomy for coloring: 

taxonomy <- as.data.frame(tax_table(physeq2)[rownames(tax_table(physeq2)) %in% clusters_otus_df$seq])

main_families1 <- c("Lachnospiraceae", "Ruminococcaceae","Erysipelotrichaceae", "Lactobacillaceae", "Enterobacteriaceae", "Staphylococcaceae", "Streptococcaceae", "Enterococcaceae")

taxonomy$family <- taxonomy$Family

levels(taxonomy$family)[!(levels(taxonomy$family) %in% main_families1)] <- c("Other")

#To color the side of the heatmap by family, we need a vector of length 239 (original number of OTUs like in table X and in the same order):

fam_x_names <- as.character(rownames(taxonomy))
fam_x_names1 <- c(fam_x_names, rep("none", 182))
fam_x_names2 <- as.data.frame(fam_x_names1)

fam <- as.character(taxonomy$family)
fam1 <- c(fam, rep("none", 182)) 
fam2 <- as.data.frame(fam1)

fam3 <- cbind (fam_x_names2, fam2)

names(fam3)[names(fam3) =="fam_x_names1"]<- "x_names"

x_names<- colnames(X)
x_df <- as.data.frame(x_names)
levels(x_df$x_names)[!(levels(x_df$x_names) %in% fam3$x_names)] <- c("none")

#now order by the original order in X: 

fam4 <- fam3[match(x_df$x_names, fam3$x_names), ]
```

### **Color by family:**

#### The following code produces the basic Figure 3B with taxonomic family annotation.

```
my.col <- c("Enterobacteriaceae" = "#a6cee3", "Lactobacillaceae" = "#1f78b4", "Streptococcaceae" = "#b2df8a", "Staphylococcaceae" = "#33a02c", "Enterococcaceae" = "#fb9a99", "Lachnospiraceae" = "#e31a1c", "Erysipelotrichaceae" = "#fdbf6f", "Ruminococcaceae" = "#ff7f00", "Other" = "#a0a0a0", "none" = "white")

col_order <- fam4$fam1

cim_my.spls_fam <- cim(my.spls, comp = 1:2, xlab = "clinical variables", ylab = "OTUs", margins = c(8,18), dist.method = c("correlation", "correlation"), clust.method=c("complete", "complete"), mapping = "XY", row.sideColors = my.col[as.character(fam4$fam1)], legend = list(legend=names(my.col), col=my.col, title = "Family"), row.names = colnames(my.spls$X))
```

### **Show in format OTUs vs samples:**

#### This produces the basic Figure S4.

```
my.col_2 <- c("f01" = "#EFF3FF", "w0" = "#C6DBEF", "w1" = "#9ECAE1", "w2" = "#6BAED6", "w3" = "#4292C6", "w4"=  "#2171B5", "w5" = "#084594")

cim_my.spls_X <- cim(my.spls, comp = 1:2, xlab = "clinical variables", ylab = "OTUs", margins = c(11,7), dist.method = c("correlation", "correlation"), clust.method=c("complete", "complete"), mapping = "X", col.sideColors = my.col[as.character(fam4$fam1)], row.sideColors = my.col_2[as.character(sample_data(physeq2)$timepoint)], legend=list(legend=names(my.col_2), col=my.col_2, title = "time point"))
```

### **Look at the taxonomy of the OTUs in the clusters based on hierarchical clustering assignment from the `cim` matrix**

#### Subset the OTUs in cluster 1:

```
otus_cluster_1 <- rownames(clusters_otus_df)[clusters_otus_df$cluster =="1"]

tax_table(physeq2)[rownames(tax_table(physeq2)) %in% otus_cluster_1]#almost all Lactobacillus
```

```
## Taxonomy Table:     [14 taxa by 7 taxonomic ranks]:
##                        Kingdom    Phylum          Class        
## HM534767.1.1445        "Bacteria" "Firmicutes"    "Bacilli"    
## AF413523.1.1559        "Bacteria" "Firmicutes"    "Bacilli"    
## KF751750.1.987         "Bacteria" "Firmicutes"    "Bacilli"    
## AB257866.1.1523        "Bacteria" "Firmicutes"    "Bacilli"    
## JX986976.1.1515        "Bacteria" "Firmicutes"    "Bacilli"    
## GU451064.1.1450        "Bacteria" "Firmicutes"    "Bacilli"    
## EU774037.1.1432        "Bacteria" "Firmicutes"    "Bacilli"    
## FN667075.1.1502        "Bacteria" "Firmicutes"    "Bacilli"    
## EU461951.1.1319        "Bacteria" "Firmicutes"    "Bacilli"    
## EU460769.1.1419        "Bacteria" "Firmicutes"    "Bacilli"    
## KC836559.1.1336        "Bacteria" "Firmicutes"    "Bacilli"    
## FJ749794.1.1449        "Bacteria" "Firmicutes"    "Bacilli"    
## KF029502.1.1525        "Bacteria" "Firmicutes"    "Bacilli"    
## AUFL01000034.2017.3448 "Bacteria" "Bacteroidetes" "Bacteroidia"
##                        Order             Family              
## HM534767.1.1445        "Lactobacillales" "Lactobacillaceae"  
## AF413523.1.1559        "Lactobacillales" "Lactobacillaceae"  
## KF751750.1.987         "Lactobacillales" "Lactobacillaceae"  
## AB257866.1.1523        "Lactobacillales" "Lactobacillaceae"  
## JX986976.1.1515        "Lactobacillales" "Lactobacillaceae"  
## GU451064.1.1450        "Lactobacillales" "Lactobacillaceae"  
## EU774037.1.1432        "Lactobacillales" "Lactobacillaceae"  
## FN667075.1.1502        "Lactobacillales" "Lactobacillaceae"  
## EU461951.1.1319        "Lactobacillales" "Streptococcaceae"  
## EU460769.1.1419        "Lactobacillales" "Lactobacillaceae"  
## KC836559.1.1336        "Lactobacillales" "Streptococcaceae"  
## FJ749794.1.1449        "Lactobacillales" "Lactobacillaceae"  
## KF029502.1.1525        "Lactobacillales" "Lactobacillaceae"  
## AUFL01000034.2017.3448 "Bacteroidales"   "Porphyromonadaceae"
##                        Genus          
## HM534767.1.1445        "Lactobacillus"
## AF413523.1.1559        "Lactobacillus"
## KF751750.1.987         "Lactobacillus"
## AB257866.1.1523        "Lactobacillus"
## JX986976.1.1515        "Lactobacillus"
## GU451064.1.1450        "Lactobacillus"
## EU774037.1.1432        "Lactobacillus"
## FN667075.1.1502        "Lactobacillus"
## EU461951.1.1319        "Streptococcus"
## EU460769.1.1419        "Lactobacillus"
## KC836559.1.1336        "Streptococcus"
## FJ749794.1.1449        "Lactobacillus"
## KF029502.1.1525        "Lactobacillus"
## AUFL01000034.2017.3448 "Dysgonomonas" 
##                        Species                                       
## HM534767.1.1445        "Lactobacillus sp. Akpobro1"                  
## AF413523.1.1559        "Lactobacillus pantheris"                     
## KF751750.1.987         "uncultured bacterium"                        
## AB257866.1.1523        "Lactobacillus suebicus"                      
## JX986976.1.1515        "Lactobacillus aviarius subsp. araffinosus"   
## GU451064.1.1450        "Lactobacillus sp. 5-1-2"                     
## EU774037.1.1432        "uncultured bacterium"                        
## FN667075.1.1502        "uncultured compost bacterium"                
## EU461951.1.1319        "uncultured bacterium"                        
## EU460769.1.1419        "uncultured bacterium"                        
## KC836559.1.1336        "Streptococcus salivarius subsp. thermophilus"
## FJ749794.1.1449        "Lactobacillus fermentum"                     
## KF029502.1.1525        "Lactobacillus casei"                         
## AUFL01000034.2017.3448 "Dysgonomonas capnocytophagoides DSM 22835"
```

#### Subset the OTUs in cluster 2:

```
otus_cluster_2 <- rownames(clusters_otus_df)[clusters_otus_df$cluster =="2"]

tax_table(physeq2)[rownames(tax_table(physeq2)) %in% otus_cluster_2]
```

```
## Taxonomy Table:     [21 taxa by 7 taxonomic ranks]:
##                        Kingdom    Phylum           Class             
## DQ801646.1.1388        "Bacteria" "Firmicutes"     "Clostridia"      
## EU246837.1.1436        "Bacteria" "Firmicutes"     "Bacilli"         
## DQ804549.1.1379        "Bacteria" "Firmicutes"     "Clostridia"      
## FJ366680.1.1377        "Bacteria" "Actinobacteria" "Actinobacteria"  
## HQ749687.1.1421        "Bacteria" "Firmicutes"     "Clostridia"      
## FJ753793.1.1354        "Bacteria" "Firmicutes"     "Clostridia"      
## DQ800353.1.1293        "Bacteria" "Firmicutes"     "Clostridia"      
## EU887971.1.1372        "Bacteria" "Firmicutes"     "Clostridia"      
## JQ608258.1.1383        "Bacteria" "Firmicutes"     "Erysipelotrichia"
## AY850535.1.1224        "Bacteria" "Actinobacteria" "Actinobacteria"  
## DQ326608.1.1352        "Bacteria" "Firmicutes"     "Clostridia"      
## AJ272200.1.1497        "Bacteria" "Firmicutes"     "Bacilli"         
## EU508251.1.1415        "Bacteria" "Firmicutes"     "Erysipelotrichia"
## DQ807523.1.1284        "Bacteria" "Firmicutes"     "Clostridia"      
## CP001726.749856.751352 "Bacteria" "Actinobacteria" "Coriobacteriia"  
## HQ794871.1.1442        "Bacteria" "Firmicutes"     "Erysipelotrichia"
## JF193283.1.1349        "Bacteria" "Firmicutes"     "Clostridia"      
## DQ802363.1.1390        "Bacteria" "Firmicutes"     "Clostridia"      
## GQ175428.1.1457        "Bacteria" "Firmicutes"     "Clostridia"      
## EU778851.1.1392        "Bacteria" "Firmicutes"     "Clostridia"      
## GQ133038.1.1416        "Bacteria" "Firmicutes"     "Bacilli"         
##                        Order                Family                 
## DQ801646.1.1388        "Clostridiales"      "Lachnospiraceae"      
## EU246837.1.1436        "Bacillales"         "Staphylococcaceae"    
## DQ804549.1.1379        "Clostridiales"      "Ruminococcaceae"      
## FJ366680.1.1377        "Bifidobacteriales"  "Bifidobacteriaceae"   
## HQ749687.1.1421        "Clostridiales"      "Ruminococcaceae"      
## FJ753793.1.1354        "Clostridiales"      "Peptostreptococcaceae"
## DQ800353.1.1293        "Clostridiales"      "Lachnospiraceae"      
## EU887971.1.1372        "Clostridiales"      "Ruminococcaceae"      
## JQ608258.1.1383        "Erysipelotrichales" "Erysipelotrichaceae"  
## AY850535.1.1224        "Bifidobacteriales"  "Bifidobacteriaceae"   
## DQ326608.1.1352        "Clostridiales"      "Ruminococcaceae"      
## AJ272200.1.1497        "Lactobacillales"    "Enterococcaceae"      
## EU508251.1.1415        "Erysipelotrichales" "Erysipelotrichaceae"  
## DQ807523.1.1284        "Clostridiales"      "Lachnospiraceae"      
## CP001726.749856.751352 "Coriobacteriales"   "Coriobacteriaceae"    
## HQ794871.1.1442        "Erysipelotrichales" "Erysipelotrichaceae"  
## JF193283.1.1349        "Clostridiales"      "Family XI"            
## DQ802363.1.1390        "Clostridiales"      "Lachnospiraceae"      
## GQ175428.1.1457        "Clostridiales"      "Ruminococcaceae"      
## EU778851.1.1392        "Clostridiales"      "Ruminococcaceae"      
## GQ133038.1.1416        "Lactobacillales"    "Enterococcaceae"      
##                        Genus             
## DQ801646.1.1388        "Incertae Sedis"  
## EU246837.1.1436        "Staphylococcus"  
## DQ804549.1.1379        "Faecalibacterium"
## FJ366680.1.1377        "Bifidobacterium" 
## HQ749687.1.1421        "Incertae Sedis"  
## FJ753793.1.1354        "Incertae Sedis"  
## DQ800353.1.1293        "Blautia"         
## EU887971.1.1372        "Incertae Sedis"  
## JQ608258.1.1383        "Incertae Sedis"  
## AY850535.1.1224        "Bifidobacterium" 
## DQ326608.1.1352        "uncultured"      
## AJ272200.1.1497        "Enterococcus"    
## EU508251.1.1415        "uncultured"      
## DQ807523.1.1284        "Incertae Sedis"  
## CP001726.749856.751352 "Eggerthella"     
## HQ794871.1.1442        "Incertae Sedis"  
## JF193283.1.1349        "Finegoldia"      
## DQ802363.1.1390        "Blautia"         
## GQ175428.1.1457        "uncultured"      
## EU778851.1.1392        "Subdoligranulum" 
## GQ133038.1.1416        "Enterococcus"    
##                        Species                          
## DQ801646.1.1388        "uncultured bacterium"           
## EU246837.1.1436        "Staphylococcus sp. Cobs2Tis23"  
## DQ804549.1.1379        "uncultured bacterium"           
## FJ366680.1.1377        "uncultured bacterium"           
## HQ749687.1.1421        "uncultured organism"            
## FJ753793.1.1354        "swine fecal bacterium RF1A-Xyl2"
## DQ800353.1.1293        "uncultured bacterium"           
## EU887971.1.1372        "uncultured Clostridia bacterium"
## JQ608258.1.1383        "bacterium NLAE-zl-C558"         
## AY850535.1.1224        "uncultured bacterium"           
## DQ326608.1.1352        "uncultured bacterium"           
## AJ272200.1.1497        "Enterococcus hirae"             
## EU508251.1.1415        "uncultured bacterium"           
## DQ807523.1.1284        "uncultured bacterium"           
## CP001726.749856.751352 "Eggerthella lenta DSM 2243"     
## HQ794871.1.1442        "uncultured organism"            
## JF193283.1.1349        "uncultured bacterium"           
## DQ802363.1.1390        "uncultured bacterium"           
## GQ175428.1.1457        "uncultured bacterium"           
## EU778851.1.1392        "uncultured bacterium"           
## GQ133038.1.1416        "uncultured bacterium"
```

#### Subset the OTUs in cluster 3:

```
otus_cluster_3 <- rownames(clusters_otus_df)[clusters_otus_df$cluster =="3"]

tax_table(physeq2)[rownames(tax_table(physeq2)) %in% otus_cluster_3]
```

```
## Taxonomy Table:     [22 taxa by 7 taxonomic ranks]:
##                             Kingdom    Phylum          
## JQ448431.1.1389             "Bacteria" "Firmicutes"    
## JQ460192.1.1325             "Bacteria" "Bacteroidetes" 
## M58833.1.1526               "Bacteria" "Firmicutes"    
## HG799951.1.1622             "Bacteria" "Firmicutes"    
## JF235878.1.1342             "Bacteria" "Actinobacteria"
## JQ680457.1.1359             "Bacteria" "Actinobacteria"
## GQ379595.1.1207             "Bacteria" "Proteobacteria"
## AB787271.1.1442             "Bacteria" "Bacteroidetes" 
## AOTI010230528.6.1502        "Bacteria" "Bacteroidetes" 
## JF109069.1.1322             "Bacteria" "Firmicutes"    
## JX464212.1.1336             "Bacteria" "Firmicutes"    
## HQ778412.1.1413             "Bacteria" "Bacteroidetes" 
## FJ976590.1.1390             "Bacteria" "Proteobacteria"
## KF178309.1.1501             "Bacteria" "Firmicutes"    
## HQ703879.1.1491             "Bacteria" "Firmicutes"    
## FJ950694.1.1472             "Bacteria" "Proteobacteria"
## JF164165.1.1375             "Bacteria" "Firmicutes"    
## KC463799.1.1243             "Bacteria" "Proteobacteria"
## JF146381.1.1370             "Bacteria" "Firmicutes"    
## AM900778.1.1535             "Bacteria" "Firmicutes"    
## CBVB010000006.254456.255954 "Bacteria" "Bacteroidetes" 
## AB013258.1.1500             "Bacteria" "Proteobacteria"
##                             Class                 Order              
## JQ448431.1.1389             "Bacilli"             "Lactobacillales"  
## JQ460192.1.1325             "Bacteroidia"         "Bacteroidales"    
## M58833.1.1526               "Bacilli"             "Lactobacillales"  
## HG799951.1.1622             "Bacilli"             "Bacillales"       
## JF235878.1.1342             "Actinobacteria"      "Micrococcales"    
## JQ680457.1.1359             "Actinobacteria"      "Corynebacteriales"
## GQ379595.1.1207             "Gammaproteobacteria" "Xanthomonadales"  
## AB787271.1.1442             "Bacteroidia"         "Bacteroidales"    
## AOTI010230528.6.1502        "Flavobacteriia"      "Flavobacteriales" 
## JF109069.1.1322             "Bacilli"             "Bacillales"       
## JX464212.1.1336             "Bacilli"             "Bacillales"       
## HQ778412.1.1413             "Bacteroidia"         "Bacteroidales"    
## FJ976590.1.1390             "Gammaproteobacteria" "Enterobacteriales"
## KF178309.1.1501             "Bacilli"             "Lactobacillales"  
## HQ703879.1.1491             "Bacilli"             "Lactobacillales"  
## FJ950694.1.1472             "Gammaproteobacteria" "Enterobacteriales"
## JF164165.1.1375             "Bacilli"             "Bacillales"       
## KC463799.1.1243             "Gammaproteobacteria" "Enterobacteriales"
## JF146381.1.1370             "Bacilli"             "Lactobacillales"  
## AM900778.1.1535             "Bacilli"             "Bacillales"       
## CBVB010000006.254456.255954 "Bacteroidia"         "Bacteroidales"    
## AB013258.1.1500             "Betaproteobacteria"  "Neisseriales"     
##                             Family               Genus                 
## JQ448431.1.1389             "Streptococcaceae"   "Streptococcus"       
## JQ460192.1.1325             "Prevotellaceae"     "Prevotella"          
## M58833.1.1526               "Lactobacillaceae"   "Pediococcus"         
## HG799951.1.1622             "Staphylococcaceae"  "Staphylococcus"      
## JF235878.1.1342             "Microbacteriaceae"  "Naasia"              
## JQ680457.1.1359             "Corynebacteriaceae" "Corynebacterium"     
## GQ379595.1.1207             "Xanthomonadaceae"   "Stenotrophomonas"    
## AB787271.1.1442             "Bacteroidaceae"     "Bacteroides"         
## AOTI010230528.6.1502        "Flavobacteriaceae"  "Chryseobacterium"    
## JF109069.1.1322             "Staphylococcaceae"  "Staphylococcus"      
## JX464212.1.1336             "Paenibacillaceae"   "Paenibacillus"       
## HQ778412.1.1413             "Bacteroidaceae"     "Bacteroides"         
## FJ976590.1.1390             "Enterobacteriaceae" "Enterobacter"        
## KF178309.1.1501             "Lactobacillaceae"   "Pediococcus"         
## HQ703879.1.1491             "Carnobacteriaceae"  "Alkalibacterium"     
## FJ950694.1.1472             "Enterobacteriaceae" "Escherichia-Shigella"
## JF164165.1.1375             "Staphylococcaceae"  "Staphylococcus"      
## KC463799.1.1243             "Enterobacteriaceae" "Klebsiella"          
## JF146381.1.1370             "Streptococcaceae"   "Streptococcus"       
## AM900778.1.1535             "Paenibacillaceae"   "Paenibacillus"       
## CBVB010000006.254456.255954 "Bacteroidaceae"     "Bacteroides"         
## AB013258.1.1500             "Neisseriaceae"      "uncultured"          
##                             Species                          
## JQ448431.1.1389             "uncultured bacterium"           
## JQ460192.1.1325             "uncultured bacterium"           
## M58833.1.1526               "Pediococcus acidilactici"       
## HG799951.1.1622             "Staphylococcus epidermidis"     
## JF235878.1.1342             "uncultured bacterium"           
## JQ680457.1.1359             "Corynebacterium sp. DYS15"      
## GQ379595.1.1207             "uncultured bacterium"           
## AB787271.1.1442             "Bacteroides sp. UasXn-3"        
## AOTI010230528.6.1502        "Triticum urartu"                
## JF109069.1.1322             "uncultured bacterium"           
## JX464212.1.1336             "Paenibacillus sp. BJC15-C21"    
## HQ778412.1.1413             "uncultured organism"            
## FJ976590.1.1390             "Enterobacter sp. LCR81"         
## KF178309.1.1501             "Pediococcus pentosaceus"        
## HQ703879.1.1491             "uncultured bacterium"           
## FJ950694.1.1472             "Escherichia coli"               
## JF164165.1.1375             "uncultured bacterium"           
## KC463799.1.1243             "uncultured bacterium"           
## JF146381.1.1370             "uncultured bacterium"           
## AM900778.1.1535             "Paenibacillus sp. PA215"        
## CBVB010000006.254456.255954 "Bacteroidaceae bacterium MS4"   
## AB013258.1.1500             "uncultured beta proteobacterium"
```

### Also subset the OTUs that had the maximum loadings in the first 2 components in the resluts of the sPLS regression

```
OTUs_main <- rownames(my.spls$loadings$X)[my.spls$loadings$X[,1]!=0 | my.spls$loadings$X[,2]!=0]
```

#### 57 OTUS have loadings `!=0` in comp 1 and/or 2

### **Plotting the loadings**

#### Loading plots of OTUs with maximum contributions on the 1st and 2nd component, respectively.

#### The following code produces the basic Figure 3C:

```
par(mfrow=c(1,2), oma = c(1,1,1,0))
plotLoadings(my.spls, comp = 1, method = 'mean', contrib = 'max', block = 'X', xlim =c(-0.6, 0.2), size.name = 0.5, size.title = 1)
plotLoadings(my.spls, comp = 2, method = 'mean', contrib = 'max', block = 'X', xlim =c(-0.5,0.4), size.name = 0.5, size.title = 1)
```

```
par(mfrow=c(1,1))
```

#### Loading plots of clinical variables with maximum contributions on the 1st and 2nd component, respectively.

```
par(mfrow=c(1,2))
plotLoadings(my.spls, comp = 1, method = 'mean', contrib = 'max', block = 'Y', size.title = 1)
plotLoadings(my.spls, comp = 2, method = 'mean', contrib = 'max', block = 'Y', size.title = 1)
```

```
par(mfrow=c(1,1))
```

## **Canonical correspondence analysis (CCpnA)**

#### Using package `vegan`

#### CcpnA requires the use of absolute OTU counts, i.e. it cannot handle variance stabilized data from `Deseq2`. Therefore we make a version of the current phyloseq object where the OTU table is replaced with the original absolute counts (Therefore use the OTU counts from the phyloseq object stored before variance stabilization).

#### The file phy\_obj1\_core\_cleaned.Rdata is provided.

```
phy_obj1_core_cleaned <- readRDS(file = "M:/Documents/Publications/Masche_R_Scripts_and_Data/Data/phy_obj1_core_cleaned.Rdata")

physeq_absolute <- physeq2

#exclude extr control from abs count otu_table:
NC2 <- grep("negative_control", sample_names(phy_obj1_core_cleaned), value=TRUE) #grep extraction control
phy_obj1_core_cleaned_wo_NC <- prune_samples(! sample_names(phy_obj1_core_cleaned) %in% NC2, phy_obj1_core_cleaned)#subset extraction control 

all(rownames(otu_table(physeq_absolute)) == rownames(otu_table(phy_obj1_core_cleaned_wo_NC)))
```

```
## [1] TRUE
```

```
all(colnames(otu_table(physeq_absolute)) == colnames(otu_table(phy_obj1_core_cleaned_wo_NC)))
```

```
## [1] TRUE
```

```
otu_table(physeq_absolute) <- otu_table(phy_obj1_core_cleaned)
```

### Save a version of this `phyloseq` object with absolute counts that can be used for the patient profiles (Script 7) (because we want to show relative abundance instead of vst stablized data for better overview there)

```
###Write the phyloseq object to a file
#saveRDS(physeq_absolute, file = "M:/Documents/Publications/Masche_R_Scripts_and_Data/Data/physeq_absolute.Rdata")
```

### Subset the 57 OTUs that are potentially most relevant from the sPLS regression analysis:

```
physeq_absolute_from_spls <- prune_taxa(OTUs_main, physeq_absolute)
```

#### Convert data from `phyloseq` to data frame format

```
df_sample_veg_cca_from_spls <- as(sample_data(physeq_absolute_from_spls), "data.frame")

df_taxa_veg_cca_from_spls <- as.data.frame(t(otu_table(physeq_absolute_from_spls)))
```

#### CcpnA is performed with the variables that were identified as potentially relevant in the adonis analysis and the OTUs identified as relevant in the sPLS

```
veg_cca_from_spls <- vegan::cca(df_taxa_veg_cca_from_spls ~  Rec.age.in.y + CRP_tp_mean + CRP_week1 + CRP_week5 + CRP_week6 + CRP_3months + CRP_6months + hbd2_before + hbd2_week0 + hbd2_week1 + hbd2_week2 + CitDayMinus7 + CitDay7 + p3p4_dag60 + p3p8_dag30 + p45p19_dag60 + p45p20_dag60 + n20p19_dag60 + n3p16p56_dag30 + n3p16p56_dag60 + mean_mono_before + mean_mono_week3 + mean_neutro_3months + Sex + Donor + malignant_benign + GVHD_factor + Alive...1.yes..0.no., data=df_sample_veg_cca_from_spls)

#summary(veg_cca_from_spls)
```

#### **Extract the scores from the CCpnA results and plot them with `ggplot2`:**

#### Code in this chunk was modified after Callahan BJ, Sankaran K, Fukuyama JA, McMurdie PJ, Holmes SP. Bioconductor workflow for microbiome data analysis: from raw reads to community analyses. F1000Research. 2016;5:1492. doi:10.12688/f1000research.8986.1.

```
tax <- tax_table(physeq_absolute_from_spls)@.Data %>% data.frame(stringsAsFactors = FALSE)

tax$seq <- rownames(tax)

main_orders <- c("Clostridiales", "Lactobacillales")

tax$Order[!(tax$Order %in% main_orders)] <- "Other"
tax$Order <- factor(tax$Order, levels = c(main_orders, "Other"))

main_families <- c("Lachnospiraceae", "Ruminococcaceae","Erysipelotrichaceae", "Lactobacillaceae")

tax$Family[!(tax$Family %in% main_families)] <- "Other"
tax$Family <- factor(tax$Family, levels = c(main_families, "Other"))

tax$otu_id <- seq_len(nrow(otu_table(physeq_absolute_from_spls)))

ps_scores <- vegan::scores(veg_cca_from_spls, display=c("sp","wa","cn","bp"), choices=c(1,2,3))

sites <- data.frame(ps_scores$sites)
sites$feces_sample <- rownames(sites)

sites <- sites %>%  left_join(sample_data(physeq_absolute_from_spls) %>% mutate(feces_sample = rownames(sample_data(physeq_absolute_from_spls)), by = 'feces_sample'))
```

```
## Warning in class(x) <- c(subclass, tibble_class): Setting class(x) to
## multiple strings ("tbl_df", "tbl", ...); result will no longer be an S4
## object
```

```
## Joining, by = "feces_sample"
```

```
species <- data.frame(ps_scores$species)
species$otu_id <- seq_along(rownames(otu_table(physeq_absolute_from_spls)))
species <- species %>%  left_join(tax)
```

```
## Joining, by = "otu_id"
```

```
cat_vars <- data.frame(ps_scores$centroids)
  
cont_vars <- data.frame(ps_scores$biplot)
cont_vars <- cont_vars[! rownames(cont_vars) %in% rownames(cat_vars),]

evals_prop <- 100 * veg_cca_from_spls$CCA$eig[1:3] / sum(veg_cca_from_spls$CA$eig)
```

## Subset variables with scores >0.2/<-0.2

```
cont_vars_main <- rownames(cont_vars)[cont_vars[,"CCA1"] >0.2 | cont_vars[,"CCA2"] >0.2 |  cont_vars[,"CCA1"] < -0.2 | cont_vars[,"CCA2"] < -0.2] #none filtered out (at 0.2)

cont_vars_main <-  cont_vars[rownames(cont_vars) %in% cont_vars_main,]

cat_vars_main <- rownames(cat_vars)[cat_vars[,"CCA1"] >0.2 | cat_vars[,"CCA2"] >0.2 |  cat_vars[,"CCA1"] < -0.2 | cat_vars[,"CCA2"] < -0.2] #malignant and graft_bone_marrow out (at 0.2)

cat_vars_main <-  cat_vars[rownames(cat_vars) %in% cat_vars_main,]

#also for species: 

species_main <- rownames(species)[species[,"CCA1"] >0.2 | species[,"CCA2"] >0.2 |  species[,"CCA1"] < -0.2 | species[,"CCA2"] < -0.2] 

species_main <- species[rownames(species) %in% species_main,]

species_main_clusters <- left_join(species_main, clusters_otus_df, by="seq")
```

```
## Warning: Column `seq` joining character vector and factor, coercing into
## character vector
```

## Make nicer labels for the variables

```
rownames(cont_vars_main) <- c("Age", "CRP", "CRP_w1", "CRP_w5", "CRP_w6", "CRP_m3", "hBD2_pre", "hBD2_w1", "hBD2_w2", "Citr_pre", "Citr_w1", "CD4+_m2", "B_m2", "mat_B_m2", "NK_m1", "NK_m2", "mono_pre", "mono_w3", "neutro_m3")

rownames(cat_vars_main) <- c("Female", "sibling_donor", "unrel_donor", "benign", "aGvHD_0-I", "aGvHD_II-IV", "Survival_no", "Survival_yes")
```

#### The following code produces the basic Figure S3A:

```
cca_plot_02 <- ggplot() +stat_ellipse(data=species_main_clusters, aes(x=CCA1, y=CCA2, group=cluster, fill= factor(cluster)), geom="polygon", alpha=0.6, type = "norm", level=0.8, linetype = "solid")+ scale_fill_manual(values = c("#a6cee3","#A0A0A0","#fdbf6f"))+  geom_point(data = sites, aes(x = CCA1, y = CCA2),shape=2, alpha = 0.5) +  geom_point(data = species_main_clusters, aes(x = CCA1, y = CCA2, col = cluster), size = 2, alpha = 0.8) +  guides(fill =FALSE, col = guide_legend(override.aes = list(size = 3))) + labs(x = sprintf("Axis1 [%s%% variance]", round(evals_prop[1], 2)), y = sprintf("Axis2 [%s%% variance]", round(evals_prop[2], 2))) + coord_fixed(sqrt(veg_cca_from_spls$CCA$eig[2] / veg_cca_from_spls$CCA$eig[1]))+ scale_colour_manual(values = c("#1f78b4", "#616161", "#ff7f00"))  +  theme(legend.position="none", panel.border = element_rect(color = "#787878", fill = alpha("white", 0)),panel.grid.minor = element_blank(), panel.grid.major = element_blank(), panel.background = element_blank()) + geom_point(data= cat_vars_main, x=cat_vars_main[,"CCA1"], y= cat_vars_main[,"CCA2"], shape= 3, alpha = 0.8)+ geom_text(aes(x=cat_vars_main[,"CCA1"], y= cat_vars_main[,"CCA2"],label=rownames(cat_vars_main)),hjust=0, vjust=0, size=4, alpha = 0.9, color = "#787878") +  geom_segment(aes(x =0, y = 0, xend=cont_vars_main[,"CCA1"], yend=cont_vars_main[,"CCA2"]), arrow = arrow(length = unit(0.25, "cm")), size=0.5, colour = "black", alpha = 0.6) + geom_text(aes(x=cont_vars_main[,"CCA1"], y= cont_vars_main[,"CCA2"], label=rownames(cont_vars_main)),hjust=0, vjust=0, color = "black", size = 4, alpha=0.9) + geom_hline(yintercept=0, linetype = "dashed", color = "grey") + geom_vline(xintercept=0, linetype = "dashed", color = "grey") 

cca_plot_02
```

#### The following code produces the basic Figure 4:

```
#to "zoom in":
cca_plot_02 + xlim(-0.6,1.45) +ylim(-1.2,2.4)
```

```
## Warning: Removed 7 rows containing non-finite values (stat_ellipse).
```

```
## Warning: Removed 9 rows containing missing values (geom_point).
```

```
## Warning: Removed 7 rows containing missing values (geom_point).
```

### **Run a CCpnA including antibiotics treatment:**

```
veg_cca_from_spls_antibio <- vegan::cca(df_taxa_veg_cca_from_spls ~  Rec.age.in.y + CRP_tp_mean + CRP_week1 + CRP_week5 + CRP_week6 + CRP_3months + CRP_6months + hbd2_before + hbd2_week0 + hbd2_week1 + hbd2_week2 + CitDayMinus7 + CitDay7 + p3p4_dag60 + p3p8_dag30 + p45p19_dag60 + p45p20_dag60 + n20p19_dag60 + n3p16p56_dag30 + n3p16p56_dag60 + mean_mono_before + mean_mono_week3 + mean_neutro_3months 
+ Sulfamethoxazole_Trimethoprim_tp              
+  Ceftazidim_tp                                 
+  Meropenem_tp                                  
+  Sulfamethizole_tp                             
+  Chloramphenicol_tp                            
+  Vancomycin_tp
+  Cefuroxime_tp                                  
+  Hexamycin_tp                                  
+  Ciprofloxacin_tp                              
+  Piperacillin_tp                               
+  Colistin_tp                                   
+  Linezolid_tp                                  
+  Metronidazol_tp                               
+  Teicoplanin_tp                                
+  Amoxicillin_tp                                
+  Fusidic_acid_tp                               
+  Dicloxacillin_tp 
+ Sex + Donor + malignant_benign + GVHD_factor + Alive...1.yes..0.no. + graft, data=df_sample_veg_cca_from_spls)
```

### Extract the scores

```
ps_scores_antibio <- vegan::scores(veg_cca_from_spls_antibio, display=c("sp","wa","cn","bp"), choices=c(1,2,3))

sites_antibio <- data.frame(ps_scores_antibio$sites)
sites_antibio$feces_sample <- rownames(sites_antibio)
sites_antibio <- sites_antibio %>% left_join(sample_data(physeq_absolute_from_spls)%>% mutate(feces_sample = rownames(sample_data(physeq_absolute_from_spls)), by = 'feces_sample'))
```

```
## Warning in class(x) <- c(subclass, tibble_class): Setting class(x) to
## multiple strings ("tbl_df", "tbl", ...); result will no longer be an S4
## object
```

```
## Joining, by = "feces_sample"
```

```
species_antibio <- data.frame(ps_scores_antibio$species)
species_antibio$otu_id <- seq_along(rownames(otu_table(physeq_absolute_from_spls)))
species_antibio <- species_antibio %>%  left_join(tax)
```

```
## Joining, by = "otu_id"
```

```
cat_vars_antibio <- data.frame(ps_scores_antibio$centroids)
  
cont_vars_antibio <- data.frame(ps_scores_antibio$biplot)
cont_vars_antibio <- cont_vars_antibio[! rownames(cont_vars_antibio) %in% rownames(cat_vars_antibio),]


evals_prop <- 100 * veg_cca_from_spls$CCA$eig[1:3] / sum(veg_cca_from_spls$CA$eig)
```

### Join cluster info

```
species_antibio_clusters <- left_join(species_antibio, clusters_otus_df, by="seq")
```

```
## Warning: Column `seq` joining character vector and factor, coercing into
## character vector
```

#### Subset >0.2 /<-0.2 only for `cont_vars` and `cat_vars` so that only the most important antibiotics are shown and we get a better overview

```
cont_vars_antibio_main <- rownames(cont_vars_antibio)[cont_vars_antibio[,"CCA1"] >0.2 | cont_vars_antibio[,"CCA2"] >0.2 |  cont_vars_antibio[,"CCA1"] < -0.2 | cont_vars_antibio[,"CCA2"] < -0.2] 

cont_vars_antibio <-  cont_vars_antibio[rownames(cont_vars_antibio) %in% cont_vars_antibio_main,]


cat_vars_antibio_main <- rownames(cat_vars_antibio)[cat_vars_antibio[,"CCA1"] >0.2 | cat_vars_antibio[,"CCA2"] >0.2 |  cat_vars_antibio[,"CCA1"] < -0.2 | cat_vars_antibio[,"CCA2"] < -0.2] 

cat_vars_antibio <-  cat_vars_antibio[rownames(cat_vars_antibio) %in% cat_vars_antibio_main,]
```

### Make nicer labels for the variables

```
rownames(cont_vars_antibio) <-  c("CRP", "CRP_w1", "CRP_w5", "CRP_w6", "CRP_m3", "hBD2_pre", "hBD2_w1", "hBD2_w2", "Citr_pre", "Citr_w1", "CD4+_m2", "B_m2", "mat_B_m2", "NK_m1", "NK_m2", "mono_pre", "mono_w3", "neutro_m3")

rownames(cat_vars_antibio) <- c("Ceftazidim", "Sulfamethizole", "Chloramphenicol", "Vancomycin_0", "Vancomycin", "Cefuroxime", "Hexamycin", "Ciprofloxacin_0", "Ciprofloxacin", "Piperacillin", "Colistin", "Linezolid", "Metronidazol", "Teicoplanin", "Amoxicillin", "Fusidic_acid", "Dicloxacillin", "Female", "sibling_donor", "unrelated_donor", "benign", "aGVHD_0-I", "aGVHD_II-IV", "Survival_no", "Survival_yes", "graft_BM/UC", "graft_UC")
```

### The following code produces the basic Figure S3B:

```
cca_plot_antibio_02 <- ggplot() +stat_ellipse(data=species_antibio_clusters, aes(x=CCA1, y=CCA2, group=cluster, fill= factor(cluster)), geom="polygon", alpha=0.6, type = "norm", level=0.8, linetype = "solid")+ scale_fill_manual(values = c("#a6cee3", "#A0A0A0", "#fdbf6f"))+  geom_point(data = sites_antibio, aes(x = CCA1, y = CCA2),shape=2, alpha = 0.5) +  geom_point(data = species_antibio_clusters, aes(x = CCA1, y = CCA2, col = cluster), size = 2, alpha = 0.8) +  guides(fill =FALSE, col = guide_legend(override.aes = list(size = 3))) + labs(x = sprintf("Axis1 [%s%% variance]", round(evals_prop[1], 2)), y = sprintf("Axis2 [%s%% variance]", round(evals_prop[2], 2))) + coord_fixed(sqrt(veg_cca_from_spls$CCA$eig[2] / veg_cca_from_spls$CCA$eig[1]))+ scale_colour_manual(values = c("#1f78b4", "#616161", "#ff7f00"))  +  theme(legend.position="none", panel.border = element_rect(color = "#787878", fill = alpha("white", 0)),panel.grid.minor = element_blank(), panel.grid.major = element_blank(), panel.background = element_blank()) + geom_point(data= cat_vars_antibio, x=cat_vars_antibio[,"CCA1"], y= cat_vars_antibio[,"CCA2"], shape= 3, alpha = 0.8)+ geom_text_repel(aes(x=cat_vars_antibio[,"CCA1"], y= cat_vars_antibio[,"CCA2"],label=rownames(cat_vars_antibio)),hjust=0, vjust=0, size=4, alpha = 0.9, color = "#787878", face = "bold") +  geom_segment(aes(x =0, y = 0, xend=cont_vars_antibio[,"CCA1"], yend=cont_vars_antibio[,"CCA2"]), arrow = arrow(length = unit(0.25, "cm")), size=0.5, colour = "black", alpha = 0.6) + geom_text_repel(aes(x=cont_vars_antibio[,"CCA1"], y= cont_vars_antibio[,"CCA2"], label=rownames(cont_vars_antibio)),hjust=0, vjust=0, color = "black", size = 4, alpha=0.9) + geom_hline(yintercept=0, linetype = "dashed", color = "grey") + geom_vline(xintercept=0, linetype = "dashed", color = "grey")
```

```
## Warning: Ignoring unknown parameters: face
```

```
cca_plot_antibio_02
```
