# Supplementary material for: Specific gut microbiome members are associated with distinct immune markers in pediatric allogeneic hematopoietic stem cell transplantation
Source: Microbiome. 2019 Sep 13;7:131. doi: 10.1186/s40168-019-0745-z (PMC6744702; doi:10.1186/s40168-019-0745-z)
Supplement: Supplementary file 16 — R data analysis report 6. Clustering of samples into Community State Types (CSTs) based on Jenson-Shannon divergence. (HTML 2338 kb) [file 40168_2019_745_MOESM16_ESM.html]

Clustering of samples into Community State Types (CSTs) based on Jenson-Shannon divergence


# Clustering of samples into Community State Types (CSTs) based on Jenson-Shannon divergence

#### *Anna Caecilia Masche*

#### *March 2019*

This is the 6th script used for data analysis and figure generation for the the manuscript by Masche et al. titled “Specific gut microbiome members are associated with distinct immune markers in allogeneic hematopoietic stem cell transplantation”.

This script and associated data are provided by Anna Cäcilia Masche, Susan Holmes, and Sünje Johanna Pamp.

These data and the associated script are licensed under the Creative Commons Attribution-ShareAlike 4.0 International License. To view a copy of this license, visit http://creativecommons.org/licenses/by-sa/4.0/ or send a letter to Creative Commons, PO Box 1866, Mountain View, CA 94042, USA.

Under the condition that appropriate credit is provided, you are free to: 1) Share, copy and redistribute the material in any medium or format 2) Adapt, remix, transform, and build upon the material for any purpose, even commercially.

To see the full license associated with attribution of this work, see the CC-By-CA license, see http://creativecommons.org/licenses/by-sa/4.0/.

The local filename is: Script6\_CSTs.Rmd.

The code in this script was partially modified after analyses performed in DiGiulio DB, Callahan BJ, McMurdie PJ, Costello EK, Lyell DJ, Robaczewska A, et al. “Temporal and spatial variation of the human microbiota during pregnancy.” Proc Natl Acad Sci. 2015;112:11060-5. doi:10.1073/pnas.1502875112.

### Here, we clustered samples into community state types (CSTs) by partitioning around medoid (PAM) clustering based on Jenson-Shannon divergence of variance stabilized microbial count data. The number of clusters was determined by gap statistic evaluation and Silhouette width quality validation. We further assessed patients’ transitions between CSTs over time.

## **Install required packages from CRAN and Bioconductor:**

```
#pkgs_needed = c("cluster", "clusterSim", "factoextra")

#letsinstall = setdiff(pkgs_needed, installed.packages(dependencies = TRUE)) 

#if (length(letsinstall) > 0) {
#  source("http://bioconductor.org/biocLite.R")
#  biocLite(letsinstall)
#}
```

```
library("cluster")
library("vegan")
```

```
## Warning: package 'vegan' was built under R version 3.4.1
```

```
## Loading required package: permute
```

```
## Warning: package 'permute' was built under R version 3.4.1
```

```
## Loading required package: lattice
```

```
## This is vegan 2.4-3
```

```
library("phyloseq")
library("ggplot2")
library("RColorBrewer")
library("grid")
library("plyr")
library("clusterSim")
```

```
## Warning: package 'clusterSim' was built under R version 3.4.1
```

```
## Loading required package: MASS
```

```
## 
## This is package 'modeest' written by P. PONCET.
## For a complete list of functions, use 'library(help = "modeest")' or 'help.start()'.
```

```
#library("ggbiplot")
#library("ggord")
library("factoextra")
```

```
## Warning: package 'factoextra' was built under R version 3.4.1
```

```
library("gridExtra")

theme_set(theme_bw())
```

## **Load the data (the previously created phyloseq object without contaminants (“cleaned”) and only “core” OTUs). This is also the version which is already transformed to relative abundance:**

```
otu_file <- readRDS("phy_obj1_core_cleaned_vsd1.Rdata")
```

### Exclude negative control from OTU table:

```
NC <- grep("negative_control", sample_names(otu_file), value=TRUE) #grep extraction control
otu_file <- prune_samples(!sample_names(otu_file) %in% NC, otu_file)#subset w/o extraction control
```

## **Prepare for clustering into CSTs**

### Determine dissimilarities by Jenson-Shannon divergence:

#### How to decide on the number of dimensions: Determine where the decrease of eigenvalues levels off.

```
#distance matrix:
jsd_dist <- phyloseq::distance(otu_file, method = "jsd")

ord = ordinate(otu_file, method = "MDS", distance = jsd_dist)
plot_scree(ord) + ggtitle("MDS-jsd ordination eigenvalues") + theme(axis.title.x = element_text(size = 20), axis.title.y = element_text(size = 20), axis.text.x = element_text(size = 11), axis.text.y = element_text(size = 13))
```

```
evs <- ord$value$Eigenvalues
print(evs[1:20])
```

```
##  [1] 0.198483488 0.103735848 0.083521316 0.057284918 0.049587675
##  [6] 0.045729401 0.043520074 0.035464268 0.028960300 0.025887888
## [11] 0.021604699 0.019911361 0.016659111 0.015704940 0.015276461
## [16] 0.013283657 0.011976520 0.011567536 0.010910678 0.009763796
```

```
print(tail(evs))
```

```
## [1] -0.005546544 -0.006064236 -0.006704185 -0.007606725 -0.009081509
## [6] -0.010421614
```

The scree plot suggests that the decrease levels off from around 4.

### **Determine the number of clusters**

#### To identify the most appropriate number of clusters to separate the data into, we employ the gap statistic with 4 MDS dimensions.

```
NDIM <- 4
x <- ord$vectors[,1:NDIM]  # rows=sample, cols=MDS axes, entries = value
pamPCoA = function(x, k) {
    list(cluster = pam(x[,1:2], k, cluster.only = TRUE))
}
gs = clusGap(x, FUN = pamPCoA, K.max = 12, B = 50)
plot_clusgap(gs) + scale_x_continuous(breaks=c(seq(0, 12, 2)))
```

We observe 4 to be the local maximum and the slope is getting much smaller after 4, so we go with 4 clusters.

## **Cluster into CSTs**

#### Perform partitioning around medoids (PAM) 4-fold clustering:

```
K <- 4
x <- ord$vectors[,1:NDIM]
data.cluster <- pam(x, k=K, cluster.only=T)
clust <- as.factor(pam(x, k=K, cluster.only=T))
#Swapping the assignment for consistency in colors between this analysis and the CCpnA and the sPLS:
clust[clust==1] <- NA
clust[clust==4] <- 1
clust[is.na(clust)] <- 4

data.cluster[data.cluster==1]<- NA
data.cluster[data.cluster==4]<- 1
data.cluster[is.na(data.cluster)]<- 4

clust[clust==3] <- NA
clust[clust==4] <- 3
clust[is.na(clust)] <- 4

data.cluster[data.cluster==3]<- NA
data.cluster[data.cluster==4]<- 3
data.cluster[is.na(data.cluster)]<- 4

sample_data(otu_file)$CST <- clust
CSTs <- as.character(seq(K))
```

### **Evaluate clustering**

#### Inspect the results in MDS and NMDS ordinations:

```
#Color CSTs according to those used in the CCpnA and sPLS analyses:
CSTColors <- c("#1f78b4", "#ff7f00", "#616161", "#A0A0A0")[c(1,2,3,4)]
names(CSTColors) <- CSTs

CSTColorScale <- scale_colour_manual(name = "CST", values = CSTColors[1:4])

CSTFillScale <- scale_fill_manual(name = "CST", values = CSTColors[1:4])

plot_ordination(otu_file, ord, color="CST")+ CSTColorScale + geom_point(size=5)  + theme(axis.title.x = element_text(size = 20), axis.title.y = element_text(size = 20), axis.text.x = element_text(size = 13), axis.text.y = element_text(size = 13))#axes 1 and 2
```

```
plot_ordination(otu_file, ord, axes=c(1,3), color="CST") +CSTColorScale + geom_point(size=5) + theme(axis.title.x = element_text(size = 20), axis.title.y = element_text(size = 20), axis.text.x = element_text(size = 13), axis.text.y = element_text(size = 13))#axes 1 and 3
```

```
plot_ordination(otu_file, ord, axes=c(2,3), color="CST") +CSTColorScale + geom_point(size=5) + theme(axis.title.x = element_text(size = 20), axis.title.y = element_text(size = 20), axis.text.x = element_text(size = 13), axis.text.y = element_text(size = 13))#axes 2 and 3
```

```
plot_ordination(otu_file, ordinate(otu_file, method="NMDS", distance=jsd_dist), color="CST") + CSTColorScale + ggtitle("NMDS -- jsd -- By Cluster") + geom_point(size=5) + theme(axis.title.x = element_text(size = 20), axis.title.y = element_text(size = 20), axis.text.x = element_text(size = 13), axis.text.y = element_text(size = 13))
```

```
## Run 0 stress 0.2235523 
## Run 1 stress 0.2320113 
## Run 2 stress 0.2308857 
## Run 3 stress 0.2204555 
## ... New best solution
## ... Procrustes: rmse 0.03362554  max resid 0.2447548 
## Run 4 stress 0.2259984 
## Run 5 stress 0.2472378 
## Run 6 stress 0.2297661 
## Run 7 stress 0.225534 
## Run 8 stress 0.2272215 
## Run 9 stress 0.2243781 
## Run 10 stress 0.2294403 
## Run 11 stress 0.2210661 
## Run 12 stress 0.2280757 
## Run 13 stress 0.2213029 
## Run 14 stress 0.2201217 
## ... New best solution
## ... Procrustes: rmse 0.01669737  max resid 0.08240121 
## Run 15 stress 0.2524454 
## Run 16 stress 0.2250526 
## Run 17 stress 0.2266265 
## Run 18 stress 0.2203309 
## ... Procrustes: rmse 0.006856261  max resid 0.05351424 
## Run 19 stress 0.2205054 
## ... Procrustes: rmse 0.01635287  max resid 0.08345062 
## Run 20 stress 0.2204876 
## ... Procrustes: rmse 0.008973819  max resid 0.05597833 
## *** No convergence -- monoMDS stopping criteria:
##     20: stress ratio > sratmax
```

Overall, the ordinations support our approach to separate the samples into these 4 clusters eventhough some samples might fit into more than one of the clusters.

#### The following heatmaps provide a first insight into differences in abundance profiles between the clusters:

```
tax_table(otu_file) <- cbind(tax_table(otu_file), OTU_number=taxa_names(otu_file))

taxa.order <- names(sort(taxa_sums(otu_file)))
for(CST in CSTs) {
  hm <- prune_taxa(names(sort(taxa_sums(otu_file), T))[1:25], otu_file)
  hm <- prune_samples(sample_data(hm)$CST == CST, hm)
  print(plot_heatmap(hm, taxa.label= "OTU_number", taxa.order=taxa.order) + ggtitle(paste("CST:", CST))+ theme(axis.title.x = element_text(size = 20), axis.title.y = element_text(size = 20), axis.text.x = element_text(size = 13), axis.text.y = element_text(size = 13)))
}
```

#### In the following version the annotation is “genus”, but each row still represents an OTU:

```
taxa.order <- names(sort(taxa_sums(otu_file)))
for(CST in CSTs) {
  otu_hm <- prune_taxa(names(sort(taxa_sums(otu_file), T))[1:25], otu_file)
  otu_hm <- prune_samples(sample_data(otu_hm)$CST == CST, otu_hm)
  print(plot_heatmap(otu_hm, taxa.label="Genus", taxa.order=taxa.order) + ggtitle(paste("CST:", CST))+ theme(axis.title.x = element_text(size = 20), axis.title.y = element_text(size = 20), axis.text.x = element_text(size = 13), axis.text.y = element_text(size = 13)))
}
```

## **Abundance of each OTU per CST:**

#### The following code provides input for the assignment of OTUs to CSTs (Table S3)

```
#Calculate the number of samples in each group
group_sums <- as.matrix(table(sample_data(otu_file)[ ,"CST"]))[,1]

#Merge samples by summing
merged <- merge_samples(otu_table(otu_file), sample_data(otu_file)$CST)

#Divide summed OTU counts by number of samples in each group to get the mean 
#Calculation is done while taxa are columns, but then transposed in the end
x <- as.matrix(otu_table(merged))
if(taxa_are_rows(merged)){ x<-t(x) }
out <- t(x/group_sums)

#Return new phyloseq object with taxa as rows
out <- otu_table(out, taxa_are_rows = TRUE)
otu_table(merged) <- out
```

## **Heatmap of the 50 most abundant OTUs with top annotations colored by CST, survival outcome and aGvHD grade:**

First define functions to create the heatmap:

```
make_hcb <- function(data, var, name = NULL, fillScale = NULL, ...) {
  hcb <- ggplot(data=data, aes_string(x="index", y=1, fill=var)) + 
          geom_raster() +
          scale_y_continuous(expand=c(0,0), breaks=1, labels=name) + 
          scale_x_continuous(expand=c(0,0)) +
          xlab(NULL) + ylab(NULL) +
          theme(axis.title=element_blank(), axis.ticks=element_blank()) +
          theme(axis.text.x=element_blank()) +
          theme(axis.text.y=element_text(size=14, face="bold")) +
          theme(plot.margin=unit(c(0,0,0,0),"lines"), 
         #       axis.ticks.margin = unit(0,"null"), ...) +
            axis.text = element_text(margin = margin(0,"null")),...) +
        guides(fill=F)
  if(!is.null(fillScale)) hcb <- hcb + fillScale
  return(hcb)
}

plot_heatmap.2 <- function(otu_file, sample.label=NULL, taxa.label=NULL, ...) {
  hm <- plot_heatmap(otu_file, taxa.label="Species", sample.order=sample.order, taxa.order = taxa.order)
  hm <- hm + theme(axis.text.x=element_blank(), axis.ticks.x=element_blank())
  #low = "#00008EFF"; high = "#7F0000FF";  na.value = "black" # From plot_heatmap defaults
  new_gradient <- scale_fill_gradientn(colours = brewer.pal(9,"YlGnBu"), na.value = "black", name="Relative\nabundance")
  hm <- hm + theme(plot.margin=unit(c(0,0.5,0.5,0.5),"lines"))
  hm <- hm + new_gradient
  hm <- hm + geom_raster() #
  hm <- hm + ylab("Taxa")
  hm$layers <- hm$layers[2] #
  return(hm)
}

mush <- function(hmap, hcbs) {
  cbgs <- lapply(hcbs, ggplotGrob)
  hmg <- ggplotGrob(hmap)
  # Make sure both plots have the same width in our final output
  cbWidths <- lapply(cbgs, function(x) x$widths[1:4])
  maxWidth <- do.call(unit.pmax, cbWidths)
  maxWidth <- unit.pmax(hmg$widths[1:4], maxWidth)
  
  # For visibility, set to the maximum width
  hmg$widths[1:4] <- as.list(maxWidth)
  for(i in seq_along(cbgs)) {
    cbgs[[i]]$widths[1:5] <- as.list(unit.c(maxWidth, hmg$widths[5]+hmg$widths[6]))
  }
  heights <- unit.c(unit(rep(1,length(cbgs)), "lines"), unit(1, "null"))
  rval <- do.call(arrangeGrob, args = c(cbgs, list(hmg), ncol=1, heights=list(heights)))
  return(rval)
}
```

### **Now plot the heatmap for the 50 most abundant OTUs:**

#### The following code produces the basic Figure S5:

```
top50 <- names(sort(taxa_sums(otu_file), decreasing=T))[1:50]
otu_hm <- prune_taxa(top50,otu_file)
taxa.order <- names(sort(taxa_sums(otu_hm)))

sample.order <- rownames(sample_data(otu_hm)[order(get_variable(otu_hm, "CST"))])
hm <- plot_heatmap.2(otu_hm, taxa.label="Species", sample.order=sample.order, taxa.order=taxa.order)
```

```
## Scale for 'fill' is already present. Adding another scale for 'fill',
## which will replace the existing scale.
```

```
hm <- hm + theme(axis.title.x = element_text(size=20),
                axis.title.y = element_text(size=20),
                axis.text.x = element_text(angle=90, size=10),
                axis.text.y = element_text(size=12),
                plot.title = element_text(size=22),
                legend.position = "none",
                plot.margin=unit(c(0,0,0,0),"mm"))

### CHANGING SPECIES TO TAXA ON Y-LABEL
labvec <- as(tax_table(otu_hm)[, c("OTU_number")], "character")
names(labvec) <- taxa_names(otu_hm)
labvec <- labvec[taxa.order]
labvec[is.na(labvec)] <- ""


hm <- hm + scale_y_discrete("Taxa", labels = labvec)
```

```
## Scale for 'y' is already present. Adding another scale for 'y', which
## will replace the existing scale.
```

```
hm <- hm + theme(axis.title = element_text(size=14))

hcbdf <- data.frame(sample_data(otu_hm))[sample.order,]
hcbdf$index <- seq(1,nsamples(otu_hm))
hcb <- make_hcb(hcbdf, "CST", name="Community state type", fillScale = CSTFillScale)
```

```
## Warning in unit(c(t, r, b, l), unit): NAs durch Umwandlung erzeugt
```

```
hcb <- hcb + annotate("text", x=tapply(hcbdf$index, hcbdf[,"CST",drop=T], mean), y=1, label=levels(hcbdf[,"CST",drop=T]), size=6)


#Make survival a factor:
str(hcbdf$Alive...1.yes..0.no.)
```

```
##  int [1:97] 1 1 0 0 1 1 1 1 1 0 ...
```

```
hcbdf$Alive...1.yes..0.no. <- as.factor(hcbdf$Alive...1.yes..0.no.)

survival <- make_hcb(hcbdf, "Alive...1.yes..0.no.", name="survival", fillScale = scale_fill_manual(values=c("0"="#949494", "1"="white")))
```

```
## Warning in unit(c(t, r, b, l), unit): NAs durch Umwandlung erzeugt
```

```
survival <- survival + theme(axis.text.y = element_text(size=12, face="bold", color="grey60"))

#Make GvHD a factor: 
str(hcbdf$GVHD_factor)
```

```
##  Factor w/ 2 levels "0-1","02-apr": 1 1 2 2 1 1 2 2 1 2 ...
```

```
hcbdf$GVHD_factor <- as.factor(hcbdf$GVHD_factor)
#fix naming:
levels(hcbdf$GVHD_factor)[levels(hcbdf$GVHD_factor) == "02-apr"] <- "2-4"

GVHD <- make_hcb(hcbdf, "GVHD_factor", name="Graft-versus-host disease", fillScale = scale_fill_manual(values=c("0-1"="white", "2-4"="#949494")))
```

```
## Warning in unit(c(t, r, b, l), unit): NAs durch Umwandlung erzeugt
```

```
GVHD <- GVHD + theme(axis.text.y = element_text(size=12, face="bold", color="grey60"))

Fig <- mush(hm, list(GVHD, survival, hcb))

plot(Fig)
```

## **Analyzing CST dynamics**

### **Plot the CST transitions over time**

#### The following code produces the basic Figure 5:

```
otu_df <- data.frame(sample_data(otu_file))
#fix naming:
levels(otu_df$GVHD_factor)[levels(otu_df$GVHD_factor) == "02-apr"] <- "2-4"


#order the patients in the df according to survival
otu_df$Patient_ID <- factor(otu_df$Patient_ID, levels = unique(otu_df$Patient_ID[order(-as.numeric(otu_df$GVHD_factor), -as.numeric(otu_df$Alive...1.yes..0.no.), - as.numeric(otu_df$Patient_ID))]))

otu_df$Alive...1.yes..0.no._f = factor(otu_df$Alive...1.yes..0.no., levels=c('1','0'))


time_plot <- ggplot(otu_df, aes(x=days_posttransplant, y=Patient_ID)) + CSTColorScale
time_plot +geom_line() + geom_point(aes(color=CST), size = 6) + theme(panel.grid.minor = element_blank(), panel.grid.major = element_blank(), panel.background = element_blank(), axis.title.x = element_text(size =16, face = "bold"), axis.text.x = element_text(size = 16), axis.text.y = element_text(size = 16), legend.title = element_text(size = 16, face = "bold"), legend.text = element_text(size = 16), strip.text.y = element_text(angle = 0)) + xlab("Days after HSCT") + ylab("") + facet_grid(GVHD_factor + Alive...1.yes..0.no._f~., labeller = "label_value", scales = "free_y", space = "free_y") + theme_bw()  + scale_x_continuous(breaks = seq(-35,45,7))
```

## **Cluster validation by Silhouette assessment:**

#### We used function `silhouette()` from package `cluster`

#### Average Silhouette width was s(i) = 0.16 (range: -0.02 - 0.36), with CST 1 and CST 4 being the best defined clusters (s(i) = 0.23 and 0.36, respectively). A Silhouette coefficient s(i) close to 1 indicates appropriate clustering of the respective samples.

```
obs.silhouette=mean(silhouette(data.cluster, jsd_dist)[,3])
print(obs.silhouette)
```

```
## [1] 0.1612694
```

```
sil <- silhouette(data.cluster, jsd_dist)
summary(sil)
```

```
## Silhouette of 97 units in 4 clusters from silhouette.default(x = data.cluster, dist = jsd_dist) :
##  Cluster sizes and average silhouette widths:
##          20          19          30          28 
##  0.23346890  0.08302912 -0.02329127  0.36053365 
## Individual silhouette widths:
##     Min.  1st Qu.   Median     Mean  3rd Qu.     Max. 
## -0.19098  0.03016  0.14811  0.16127  0.33247  0.53719
```

```
fviz_silhouette(sil, palette = CSTColors)
```

```
##   cluster size ave.sil.width
## 1       1   20          0.23
## 2       2   19          0.08
## 3       3   30         -0.02
## 4       4   28          0.36
```

#### We did Silhouette assessment also with K=2, K=3, K=5, K=6 and K=7 (code not shown here), and still got the best (though not optimal) quality of clustering with the initial K=4.
