# Supplementary material for: Specific gut microbiome members are associated with distinct immune markers in pediatric allogeneic hematopoietic stem cell transplantation
Source: Microbiome. 2019 Sep 13;7:131. doi: 10.1186/s40168-019-0745-z (PMC6744702; doi:10.1186/s40168-019-0745-z)
Supplement: Supplementary file 17 — R data analysis report 7. Longitudinal profiles of microbial community composition and immune markers. (HTML 4520 kb) [file 40168_2019_745_MOESM17_ESM.html]

Longitudinal profiles of microbial community composition and immune markers


# Longitudinal profiles of microbial community composition and immune markers

#### *Anna Caecilia Masche*

#### *March 2019*

This is the 7th script used for data analysis and figure generation for the the manuscript by Masche et al. titled “Specific gut microbiome members are associated with distinct immune markers in allogeneic hematopoietic stem cell transplantation”.

This script and associated data are provided by Anna Cäcilia Masche, Susan Holmes, and Sünje Johanna Pamp.

These data and the associated script are licensed under the Creative Commons Attribution-ShareAlike 4.0 International License. To view a copy of this license, visit http://creativecommons.org/licenses/by-sa/4.0/ or send a letter to Creative Commons, PO Box 1866, Mountain View, CA 94042, USA.

Under the condition that appropriate credit is provided, you are free to: 1) Share, copy and redistribute the material in any medium or format 2) Adapt, remix, transform, and build upon the material for any purpose, even commercially.

To see the full license associated with attribution of this work, see the CC-By-CA license, see http://creativecommons.org/licenses/by-sa/4.0/.

The local filename is: Script7\_profiles.Rmd.

## **Load required packages:**

```
library(reshape2)
library(ggplot2)
library(dplyr)
```

```
## Warning: package 'dplyr' was built under R version 3.4.1
```

```
## 
## Attaching package: 'dplyr'
```

```
## The following objects are masked from 'package:stats':
## 
##     filter, lag
```

```
## The following objects are masked from 'package:base':
## 
##     intersect, setdiff, setequal, union
```

```
library(plotly)
```

```
## 
## Attaching package: 'plotly'
```

```
## The following object is masked from 'package:ggplot2':
## 
##     last_plot
```

```
## The following object is masked from 'package:stats':
## 
##     filter
```

```
## The following object is masked from 'package:graphics':
## 
##     layout
```

```
library(phyloseq)
library(webshot)
```

```
## Warning: package 'webshot' was built under R version 3.4.4
```

## **Load the table**

#### The file Data\_matrix\_37\_patients\_per\_day.txt is provided.

```
dti <- read.table(file= "Data_matrix_37_patients_per_day.txt", sep = "\t", header = TRUE)
```

## **Load phyloseq object physeq\_absolute.Rdata (with absolute counts, so we can use relative abundance):**

#### The file physeq\_absolute.Rdata is provided.

```
phy_dat <- readRDS("physeq_absolute.Rdata")
```

## **Extract the diversity data that we need from the phyloseq object and merge it with the other data**

```
df_phy_dat = as(sample_data(phy_dat), "data.frame")

df_phy_dat <- tibble::rownames_to_column(df_phy_dat, var = "feces_sample")#assigns a name to the row.names column

df_phy_dat_InvSimpson <- subset(df_phy_dat, select = c(feces_sample, InvSimpson)) 

dti_2 <- left_join(dti, df_phy_dat_InvSimpson, by = "feces_sample")
```

```
## Warning: Column `feces_sample` joining factor and character vector,
## coercing into character vector
```

## **Extract the absolute OTU count data that we need from the phyloseq object and merge it with the other data**

### **Agglomerate OTU counts on family level**

```
phy_dat_otu_family = tax_glom(phy_dat, "Family")#agglom. on family level

#transform to relative abundance
phy_dat_otu_family_rel = transform_sample_counts(phy_dat_otu_family, function(x) x/sum(x))

#get the family names
fam_names <- as.data.frame(tax_table(phy_dat_otu_family_rel)[,"Family"])

#extract the family agglomerated otu table
df_phy_dat_otu_family_rel = as.data.frame(t(otu_table(phy_dat_otu_family_rel)))

#rename the columns to family names: 
colnames(df_phy_dat_otu_family_rel) <- fam_names$Family[match(names(df_phy_dat_otu_family_rel), rownames(fam_names))]

#now join with the previous data:
df_phy_dat_otu_family <- tibble::rownames_to_column(df_phy_dat_otu_family_rel, var = "feces_sample")#gives a name to the row.names column

dti_3 <- left_join(dti_2, df_phy_dat_otu_family, by = "feces_sample")
```

## **Make separate data frames for the data that should be added as event lines (GvHD onset and infections)**

```
vline.dat <- subset(dti_3, select = c(Patient_ID, days_posttransplant, GvHD_onset, infect_occur))

names(vline.dat)[names(vline.dat) == 'infect_occur'] <- 'infection' 


vline.dat_melt <- melt(vline.dat, id.vars = c("Patient_ID", "days_posttransplant"))

vline.dat_melt <- na.omit(vline.dat_melt)

vline.dat_melt_1 <- vline.dat_melt[vline.dat_melt$value == 1,]
```

## **Plot for patient 26:**

#### The following 3 code chunks produce the basic Figure 6.

```
test_26 <- dti_3[dti_3$Patient_ID == "P26",]
test_26 <- subset(test_26, days_posttransplant>-36 &  days_posttransplant<100) 

#vline data: 
vline.dat_melt_1_P26 <- vline.dat_melt_1[vline.dat_melt_1$Patient_ID == "P26",]  

#now we want to melt the otu data into 2 columns, one for abundance (abund_family) and one for taxonomy (family)

test_26_melted_fam <- reshape2::melt(test_26, id.vars = colnames(test_26[,c(1:463)]), measure.vars = colnames(test_26[,c(464:506)]), value.name = "abund_family", variable.name = "family")

#rename the less relevant families to "other" to colorcode only a few

main_families <- c("Lachnospiraceae", "Ruminococcaceae","Erysipelotrichaceae", "Lactobacillaceae", "Staphylococcaceae", "Enterobacteriaceae", "Erysipelotrichaceae", "Streptococcaceae", "Enterococcaceae")

levels(test_26_melted_fam$family)[!(levels(test_26_melted_fam$family) %in% main_families)] <- c("Other")


#now we want to agglomerate/sum the abundances of all "other" families that are less relevant

test_26_melted_fam_summed <- test_26_melted_fam %>% dplyr::group_by(Patient_ID, feces_sample, days_posttransplant, family) %>% dplyr::summarise(abund_family_summed = sum(abund_family, na.rm = TRUE))


test_26_melted_fam_summed<-as.data.frame(test_26_melted_fam_summed)


#assign colors to genera (extrapolate pallet) 

mapColours <- data.frame(family = rep(c('Ruminococcaceae', 'Erysipelotrichaceae','Lachnospiraceae', 'Enterococcaceae','Staphylococcaceae', 'Streptococcaceae','Lactobacillaceae','Enterobacteriaceae', 'Other'),133), colours = rep(c('#ff7f00', '#fdbf6f', '#e31a1c', '#fb9a99', '#33a02c', '#b2df8a', '#1f78b4','#a6cee3','#a0a0a0'), 133), stringsAsFactors = FALSE)

test_26_melted_fam_summed <- test_26_melted_fam_summed[order(match(test_26_melted_fam_summed$family,mapColours$family)),] 

b_26_comb <- plot_ly(data = test_26 , x = ~days_posttransplant, width = 800)  %>%  add_trace(data = test_26, x = ~days_posttransplant, y = ~InvSimpson, type = "scatter", mode = "lines+markers", color=I("#00868B"), connectgaps = TRUE, yaxis = "y2", showlegend =FALSE)  %>%  add_trace(x = ~days_posttransplant, y = ~hBD2_plasma_pg_ml, yaxis = "y4", color = I("#EEC900"), type = "scatter", mode = "lines+markers", connectgaps = TRUE, showlegend =FALSE) %>%  add_trace(x = ~days_posttransplant, y = ~mono, yaxis = "y5", color = I("#404040"),type = "scatter", mode = "lines+markers", connectgaps = TRUE, showlegend =FALSE) %>% add_trace(x = ~vline.dat_melt_1_P26$days_posttransplant[vline.dat_melt_1_P26$variable=="GvHD_onset"], y= c(0, 5), color = I("red"), type="scatter", mode = "lines", showlegend =FALSE) %>% add_trace(x = ~vline.dat_melt_1_P26$days_posttransplant[vline.dat_melt_1_P26$variable=="GvHD_onset"], y= 4, color = I("red"), text="GvHD onset", mode = "text", textposition = "top right", yaxis = "y2", showlegend =FALSE) %>% layout(barmode = "stack", yaxis = list(range = c(0,1), title = "", zeroline = FALSE, showline = FALSE, showticklabels = FALSE, showgrid = FALSE, side = "right"), yaxis2 = list( showline = FALSE, side = "left", title = "InvSimpson",color ="#00868B", overlaying = "y"),yaxis4 = list( showline = FALSE, side = "right", overlaying = "y",title = "hBD2",color = "#EEC900"),yaxis5 = list(showline = FALSE, side = "right", overlaying = "y", position = 1, title = "Monocytes", anchor = "free", color = "#404040"), xaxis = list(autotick=FALSE, showline = FALSE, zeroline = FALSE, dtick = 7, title = "days posttransplant", ticklen=0), showlegend =FALSE, margin = list(pad = 45, b = 90, l = 150, r = 90), legend = list(orientation ="h"))


b_26_comb <- b_26_comb %>% add_trace(data= test_26_melted_fam_summed, x=test_26_melted_fam_summed$days_posttransplant[test_26_melted_fam_summed$days_posttransplant==-27],  y=test_26_melted_fam_summed$abund_family_summed[test_26_melted_fam_summed$days_posttransplant==-27], type="bar", width = 4, color = family, marker = list(color = ~mapColours$colours), yaxis ="y", showlegend =FALSE) %>% add_trace(data= test_26_melted_fam_summed, x=test_26_melted_fam_summed$days_posttransplant[test_26_melted_fam_summed$days_posttransplant==-1],  y=test_26_melted_fam_summed$abund_family_summed[test_26_melted_fam_summed$days_posttransplant==-1], type="bar", width = 4, color = family, marker = list(color = ~mapColours$colours), yaxis ="y", showlegend =FALSE) %>% add_trace(data= test_26_melted_fam_summed, x=test_26_melted_fam_summed$days_posttransplant[test_26_melted_fam_summed$days_posttransplant==14],  y=test_26_melted_fam_summed$abund_family_summed[test_26_melted_fam_summed$days_posttransplant==14], type="bar", width = 4, color = family, marker = list(color = ~mapColours$colours), yaxis ="y", showlegend =FALSE) %>% add_trace(data= test_26_melted_fam_summed, x=test_26_melted_fam_summed$days_posttransplant[test_26_melted_fam_summed$days_posttransplant==9],  y=test_26_melted_fam_summed$abund_family_summed[test_26_melted_fam_summed$days_posttransplant==9], type="bar", width = 4, color = family, marker = list(color = ~mapColours$colours), yaxis ="y", showlegend =FALSE) %>% add_trace(data= test_26_melted_fam_summed, x=test_26_melted_fam_summed$days_posttransplant[test_26_melted_fam_summed$days_posttransplant==21],  y=test_26_melted_fam_summed$abund_family_summed[test_26_melted_fam_summed$days_posttransplant==21], type="bar", width = 4, color = family, marker = list(color = ~mapColours$colours), yaxis ="y", showlegend =FALSE) 

b_26_comb
```

```
## No trace type specified:
##   Based on info supplied, a 'scatter' trace seems appropriate.
##   Read more about this trace type -> https://plot.ly/r/reference/#scatter
```

## **Plot for patient 24:**

```
test_24 <- dti_3[dti_3$Patient_ID == "P24",]

#putting xaxis= dict(range=[-35, 210]) in the plot doesn't work for some reason, work around by subsetting

test_24 <- subset(test_24, days_posttransplant>-36 &  days_posttransplant<100) 

#vline data: 

vline.dat_melt_1_P24 <- vline.dat_melt_1[vline.dat_melt_1$Patient_ID == "P24",]  

#now we want to melt the otu data into 2 columns, one for vst count (abund_family) and one for taxonomy (family)

test_24_melted_fam <- reshape2::melt(test_24, id.vars = colnames(test_24[,c(1:463)]), measure.vars = colnames(test_24[,c(464:506)]), value.name = "abund_family", variable.name = "family")
 

#rename the less relevant families to "other" to colorcode only a few

main_families <- c("Lachnospiraceae", "Ruminococcaceae","Erysipelotrichaceae", "Lactobacillaceae", "Staphylococcaceae", "Enterobacteriaceae", "Erysipelotrichaceae", "Streptococcaceae", "Enterococcaceae")

levels(test_24_melted_fam$family)[!(levels(test_24_melted_fam$family) %in% main_families)] <- c("Other")


#now we want to agglomerate/sum the abundances of all "other" families that are less relevant

test_24_melted_fam_summed <- test_24_melted_fam %>% dplyr::group_by(Patient_ID, feces_sample, days_posttransplant, family) %>% dplyr::summarise(abund_family_summed = sum(abund_family, na.rm = TRUE))


test_24_melted_fam_summed<-as.data.frame(test_24_melted_fam_summed)


test_24_melted_fam_summed <- test_24_melted_fam_summed[order(match(test_24_melted_fam_summed$family,mapColours$family)),] 

b_24_comb <- plot_ly(data = test_24 , x = ~days_posttransplant, width = 800)  %>%  add_trace(data = test_24, x = ~days_posttransplant, y = ~InvSimpson, type = "scatter", mode = "lines+markers", color=I("#00868B"), connectgaps = TRUE, yaxis = "y2", showlegend =FALSE)  %>%  add_trace(x = ~days_posttransplant, y = ~hBD2_plasma_pg_ml, yaxis = "y4", color = I("#EEC900"), type = "scatter", mode = "lines+markers", connectgaps = TRUE, showlegend =FALSE) %>%  add_trace(x = ~days_posttransplant, y = ~mono, yaxis = "y5", color = I("#404040"),type = "scatter", mode = "lines+markers", connectgaps = TRUE, showlegend =FALSE) %>% add_trace(x = ~vline.dat_melt_1_P24$days_posttransplant[vline.dat_melt_1_P24$variable=="GvHD_onset"], y= c(0, 5), color = I("red"), type="scatter", mode = "lines", showlegend =FALSE) %>% add_trace(x = ~vline.dat_melt_1_P24$days_posttransplant[vline.dat_melt_1_P24$variable=="GvHD_onset"], y= 0.9, color = I("red"), text="GvHD onset", mode = "text", textposition = "top right", yaxis = "y", showlegend =FALSE) %>% add_trace(x = ~vline.dat_melt_1_P24$days_posttransplant[vline.dat_melt_1_P24$variable=="infection" & vline.dat_melt_1_P24$days_posttransplant <100][1], y= c(0, 1), color = I("blue"), type="scatter", mode = "lines", showlegend =FALSE, yaxis="y")%>% add_trace(x = ~vline.dat_melt_1_P24$days_posttransplant[vline.dat_melt_1_P24$variable=="infection" & vline.dat_melt_1_P24$days_posttransplant <100][2], y= c(0, 1), color = I("blue"), type="scatter", mode = "lines", showlegend =FALSE, yaxis="y") %>% add_trace(x = ~vline.dat_melt_1_P24$days_posttransplant[vline.dat_melt_1_P24$variable=="infection" & vline.dat_melt_1_P24$days_posttransplant <100][3], y= c(0, 1), color = I("blue"), type="scatter", mode = "lines", showlegend =FALSE, yaxis="y")%>% add_trace(x = ~vline.dat_melt_1_P24$days_posttransplant[vline.dat_melt_1_P24$variable=="infection" & vline.dat_melt_1_P24$days_posttransplant <100][4], y= c(0, 1), color = I("blue"), type="scatter", mode = "lines", showlegend =FALSE, yaxis="y") %>% add_trace(x = ~vline.dat_melt_1_P24$days_posttransplant[vline.dat_melt_1_P24$variable=="infection" & vline.dat_melt_1_P24$days_posttransplant <100][5], y= c(0, 1), color = I("blue"), type="scatter", mode = "lines", showlegend =FALSE, yaxis="y")%>%  add_trace(x = ~vline.dat_melt_1_P24$days_posttransplant[vline.dat_melt_1_P24$variable=="infection" & vline.dat_melt_1_P24$days_posttransplant <100][6], y= c(0, 1), color = I("blue"), type="scatter", mode = "lines", showlegend =FALSE, yaxis="y")%>% add_trace(x = ~vline.dat_melt_1_P24$days_posttransplant[vline.dat_melt_1_P24$variable=="infection" & vline.dat_melt_1_P24$days_posttransplant <100], y= 0.85, color = I("blue"), text="inf ", mode = "text", textposition = 'top left', yaxis = "y", showlegend =FALSE)  %>% add_trace(x = 0, y= c(0, 5), color = I("gray"), type="scatter", mode = "lines", line=list(dash = "dash"), showlegend =FALSE) %>% layout(barmode = "stack", yaxis = list(range = c(0,1), title = "", zeroline = FALSE, showline = FALSE, showticklabels = FALSE, showgrid = FALSE, side = "right"), yaxis2 = list( showline = FALSE, side = "left", title = "InvSimpson",color ="#00868B", overlaying = "y"),yaxis4 = list( showline = FALSE, side = "right", overlaying = "y",title = "hBD2",color = "#EEC900"),yaxis5 = list(showline = FALSE, side = "right", overlaying = "y", position = 1, title = "Monocytes", anchor = "free", color = "#404040"), xaxis = list(autotick=FALSE, showline = FALSE, zeroline = FALSE, dtick = 7, title = "days posttransplant", ticklen=0), showlegend =FALSE, margin = list(pad = 45, b = 90, l = 150, r = 90), legend = list(orientation ="h"))


b_24_comb <- b_24_comb %>% add_trace(data= test_24_melted_fam_summed, x=test_24_melted_fam_summed$days_posttransplant[test_24_melted_fam_summed$days_posttransplant==-19],  y=test_24_melted_fam_summed$abund_family_summed[test_24_melted_fam_summed$days_posttransplant==-19], type="bar", width = 4, color = family, marker = list(color = ~mapColours$colours), yaxis ="y", showlegend =FALSE) %>% add_trace(data= test_24_melted_fam_summed, x=test_24_melted_fam_summed$days_posttransplant[test_24_melted_fam_summed$days_posttransplant==21],  y=test_24_melted_fam_summed$abund_family_summed[test_24_melted_fam_summed$days_posttransplant==21], type="bar", width = 4, color = family, marker = list(color = ~mapColours$colours), yaxis ="y", showlegend =FALSE) %>% add_trace(data= test_24_melted_fam_summed, x=test_24_melted_fam_summed$days_posttransplant[test_24_melted_fam_summed$days_posttransplant==34],  y=test_24_melted_fam_summed$abund_family_summed[test_24_melted_fam_summed$days_posttransplant==34], type="bar", width = 4, color = family, marker = list(color = ~mapColours$colours), yaxis ="y") %>%  add_trace(x = ~vline.dat_melt_1_P24$days_posttransplant[vline.dat_melt_1_P24$variable=="infection" & vline.dat_melt_1_P24$days_posttransplant <100][7], y= c(0, 1), color = I("blue"), type="scatter", mode = "lines", showlegend =FALSE, yaxis="y")

b_24_comb
```

```
## No trace type specified:
##   Based on info supplied, a 'scatter' trace seems appropriate.
##   Read more about this trace type -> https://plot.ly/r/reference/#scatter
## No trace type specified:
##   Based on info supplied, a 'scatter' trace seems appropriate.
##   Read more about this trace type -> https://plot.ly/r/reference/#scatter
```

## **Plot for patient 30:**

```
test_30 <- dti_3[dti_3$Patient_ID == "P30",]

#putting xaxis= dict(range=[-35, 210]) in the plot doesn't work for some reason, work around by subsetting

test_30 <- subset(test_30, days_posttransplant>-36 &  days_posttransplant<100) 

#vline data: 

vline.dat_melt_1_P30 <- vline.dat_melt_1[vline.dat_melt_1$Patient_ID == "P30",]  

#now we want to melt the otu data into 2 colums, one for vst count (abund_family) and one for taxonomy (family)

test_30_melted_fam <- reshape2::melt(test_30, id.vars = colnames(test_30[,c(1:463)]), measure.vars = colnames(test_30[,c(464:506)]), value.name = "abund_family", variable.name = "family")
 

#rename the less relevant families to "other" to colorcode only a few

main_families <- c("Lachnospiraceae", "Ruminococcaceae","Erysipelotrichaceae", "Lactobacillaceae", "Staphylococcaceae", "Enterobacteriaceae", "Erysipelotrichaceae", "Streptococcaceae", "Enterococcaceae")

levels(test_30_melted_fam$family)[!(levels(test_30_melted_fam$family) %in% main_families)] <- c("Other")


#now we want to agglomerate/sum the abundances of all "other" families that are less relevant

test_30_melted_fam_summed <- test_30_melted_fam %>% dplyr::group_by(Patient_ID, feces_sample, days_posttransplant, family) %>% dplyr::summarise(abund_family_summed = sum(abund_family, na.rm = TRUE))


test_30_melted_fam_summed<-as.data.frame(test_30_melted_fam_summed)

test_30_melted_fam_summed <- test_30_melted_fam_summed[order(match(test_30_melted_fam_summed$family,mapColours$family)),]

b_30_comb <- plot_ly(data = test_30 , x = ~days_posttransplant, width = 800)%>%  add_trace(data = test_30, x = ~days_posttransplant, y = ~InvSimpson, type = "scatter", mode = "lines+markers", color=I("#00868B"), connectgaps = TRUE, yaxis = "y2", showlegend =FALSE)%>%  add_trace(x = ~days_posttransplant, y = ~hBD2_plasma_pg_ml, yaxis = "y4", color = I("#EEC900"), type = "scatter", mode = "lines+markers", connectgaps = TRUE, showlegend =FALSE) %>%  add_trace(x = ~days_posttransplant, y = ~mono, yaxis = "y5", color = I("#404040"),type = "scatter", mode = "lines+markers", connectgaps = TRUE, showlegend =FALSE)                                                                                                                           %>% add_trace(x = ~vline.dat_melt_1_P30$days_posttransplant[vline.dat_melt_1_P30$variable=="GvHD_onset"], y= c(0,1), yaxis="y", color = I("red"), type="scatter", mode = "lines", showlegend =FALSE)                                                                                                                     %>% add_trace(x = ~vline.dat_melt_1_P30$days_posttransplant[vline.dat_melt_1_P30$variable=="GvHD_onset"], y= 0.9, color = I("red"), text="GvHD onset", mode = "text", textposition = "top right", yaxis = "y", showlegend =FALSE)                                                                                                                                                                                                                                  %>% add_trace(x = 0, y= c(0, 1), color = I("gray"), type="scatter", mode = "lines", line=list(dash = "dash"), showlegend =FALSE, yaxis = "y5")                                                                                                                     %>%layout(barmode = "stack", yaxis = list(range = c(0,1), title = "", zeroline = FALSE, showline = FALSE, showticklabels = FALSE, showgrid = FALSE, side = "right"),  yaxis2 = list( showline = FALSE, side = "left", title = "InvSimpson",color ="#00868B", overlaying = "y"),yaxis4 = list( showline = FALSE, side = "right", overlaying = "y",title = "hBD2",color = "#EEC900"),yaxis5 = list(showline = FALSE, side = "right", overlaying = "y", position = 1, title = "Monocytes", anchor = "free", color = "#404040"), xaxis = list(autotick=FALSE, showline = FALSE, zeroline = FALSE, dtick = 7, title = "days posttransplant", ticklen=0), showlegend =FALSE, margin = list(pad = 45, b = 90, l = 150, r = 90), legend = list(orientation ="v"))
 

b_30_comb <- b_30_comb %>% add_trace(data= test_30_melted_fam_summed, x=test_30_melted_fam_summed$days_posttransplant[test_30_melted_fam_summed$days_posttransplant==-24],  y=test_30_melted_fam_summed$abund_family_summed[test_30_melted_fam_summed$days_posttransplant==-24], type="bar", width = 4, color = family, marker = list(color = ~mapColours$colours), yaxis ="y ", showlegend =TRUE) %>% add_trace(data= test_30_melted_fam_summed, x=test_30_melted_fam_summed$days_posttransplant[test_30_melted_fam_summed$days_posttransplant==-16],  y=test_30_melted_fam_summed$abund_family_summed[test_30_melted_fam_summed$days_posttransplant==-16], type="bar", width = 4, color = family, marker = list(color = ~mapColours$colours), yaxis ="y", showlegend =FALSE) %>% add_trace(data= test_30_melted_fam_summed, x=test_30_melted_fam_summed$days_posttransplant[test_30_melted_fam_summed$days_posttransplant==0],  y=test_30_melted_fam_summed$abund_family_summed[test_30_melted_fam_summed$days_posttransplant==0], type="bar", width = 4, color = family, marker = list(color = ~mapColours$colours), yaxis ="y", showlegend =FALSE)  %>% add_trace(data= test_30_melted_fam_summed, x=test_30_melted_fam_summed$days_posttransplant[test_30_melted_fam_summed$days_posttransplant==12],  y=test_30_melted_fam_summed$abund_family_summed[test_30_melted_fam_summed$days_posttransplant==12], type="bar", width = 4, color = family, marker = list(color = ~mapColours$colours), yaxis ="y", showlegend =FALSE) %>% add_trace(data= test_30_melted_fam_summed, x=test_30_melted_fam_summed$days_posttransplant[test_30_melted_fam_summed$days_posttransplant==26],  y=test_30_melted_fam_summed$abund_family_summed[test_30_melted_fam_summed$days_posttransplant==26], type="bar", width = 4, color = family, marker = list(color = ~mapColours$colours), yaxis ="y", showlegend =FALSE) 


b_30_comb
```

```
## No trace type specified:
##   Based on info supplied, a 'scatter' trace seems appropriate.
##   Read more about this trace type -> https://plot.ly/r/reference/#scatter
```

#### The following 3 code chunks produce the basic Figure S6.

## **Plot for patient 29:**

```
test_29 <- dti_3[dti_3$Patient_ID == "P29",]

#putting xaxis= dict(range=[-35, 210]) in the plot doesn't work for some reason, work around by subsetting

test_29 <- subset(test_29, days_posttransplant>-36 &  days_posttransplant<100) 

#vline data: 

vline.dat_melt_1_P29 <- vline.dat_melt_1[vline.dat_melt_1$Patient_ID == "P29",]  


#now we want to melt the otu data into 2 colums, one for vst count (abund_family) and one for taxonomy (family)

test_29_melted_fam <- reshape2::melt(test_29, id.vars = colnames(test_29[,c(1:463)]), measure.vars = colnames(test_29[,c(464:506)]), value.name = "abund_family", variable.name = "family")
 

#rename the less relevant families to "other" to colorcode only a few

main_families <- c("Lachnospiraceae", "Ruminococcaceae","Erysipelotrichaceae", "Lactobacillaceae", "Staphylococcaceae", "Enterobacteriaceae", "Erysipelotrichaceae", "Streptococcaceae", "Enterococcaceae")

levels(test_29_melted_fam$family)[!(levels(test_29_melted_fam$family) %in% main_families)] <- c("Other")


#now we want to agglomerate/sum the abundances of all "other" families that are less relevant

test_29_melted_fam_summed <- test_29_melted_fam %>% dplyr::group_by(Patient_ID, feces_sample, days_posttransplant, family) %>% dplyr::summarise(abund_family_summed = sum(abund_family, na.rm = TRUE))


test_29_melted_fam_summed<-as.data.frame(test_29_melted_fam_summed)

#order for colorcoding
#test_29_melted_fam_summed <- arrange(test_29_melted_fam_summed, family)

#assign colors to genera (extrapolate pallet) (Here the top 25 are already over the whole data set and not per patient)

test_29_melted_fam_summed <- test_29_melted_fam_summed[order(match(test_29_melted_fam_summed$family,mapColours$family)),]


b_29_comb <- plot_ly(data = test_29 , x = ~days_posttransplant, width = 800)                                                                                                                      %>%  add_trace(data = test_29, x = ~days_posttransplant, y = ~InvSimpson, type = "scatter", mode = "lines+markers", color=I("#00868B"), connectgaps = TRUE, yaxis = "y2", showlegend =FALSE)                                                                                                                                                                                                                                          %>%  add_trace(x = ~days_posttransplant, y = ~hBD2_plasma_pg_ml, yaxis = "y4", color = I("#EEC900"), type = "scatter", mode = "lines+markers", connectgaps = TRUE, showlegend =FALSE) %>%  add_trace(x = ~days_posttransplant, y = ~mono, yaxis = "y5", color = I("#404040"),type = "scatter", mode = "lines+markers", connectgaps = TRUE, showlegend =FALSE)                                                                                                                           %>% add_trace(x = ~vline.dat_melt_1_P29$days_posttransplant[vline.dat_melt_1_P29$variable=="GvHD_onset"], y= c(0,1), yaxis="y", color = I("red"), type="scatter", mode = "lines", showlegend =FALSE)                                                                                                                     %>% add_trace(x = ~vline.dat_melt_1_P29$days_posttransplant[vline.dat_melt_1_P29$variable=="GvHD_onset"], y= 0.9, color = I("red"), text="GvHD onset", mode = "text", textposition = "top right", yaxis = "y", showlegend =FALSE)                                                                                                                                                                                                                                  %>% add_trace(x = 0, y= c(0, 1), color = I("gray"), type="scatter", mode = "lines", line=list(dash = "dash"), showlegend =FALSE, yaxis = "y5")                                                                                                                     %>%layout(barmode = "stack", yaxis = list(range = c(0,1), title = "", zeroline = FALSE, showline = FALSE, showticklabels = FALSE, showgrid = FALSE, side = "right"),  yaxis2 = list( showline = FALSE, side = "left", title = "InvSimpson",color ="#00868B", overlaying = "y"),yaxis4 = list( showline = FALSE, side = "right", overlaying = "y",title = "hBD2",color = "#EEC900"),yaxis5 = list(showline = FALSE, side = "right", overlaying = "y", position = 1, title = "Monocytes", anchor = "free", color = "#404040"), xaxis = list(autotick=FALSE, showline = FALSE, zeroline = FALSE, dtick = 7, title = "days posttransplant", ticklen=0), showlegend =FALSE, margin = list(pad = 45, b = 90, l = 150, r = 90), legend = list(orientation ="v"))
 

b_29_comb <- b_29_comb %>% add_trace(data= test_29_melted_fam_summed, x=test_29_melted_fam_summed$days_posttransplant[test_29_melted_fam_summed$days_posttransplant==-12],  y=test_29_melted_fam_summed$abund_family_summed[test_29_melted_fam_summed$days_posttransplant==-12], type="bar", width = 4, color = family, marker = list(color = ~mapColours$colours), yaxis ="y ", showlegend =TRUE) %>% add_trace(data= test_29_melted_fam_summed, x=test_29_melted_fam_summed$days_posttransplant[test_29_melted_fam_summed$days_posttransplant==31],  y=test_29_melted_fam_summed$abund_family_summed[test_29_melted_fam_summed$days_posttransplant==31], type="bar", width = 4, color = family, marker = list(color = ~mapColours$colours), yaxis ="y", showlegend =FALSE) %>% add_trace(data= test_29_melted_fam_summed, x=test_29_melted_fam_summed$days_posttransplant[test_29_melted_fam_summed$days_posttransplant==35],  y=test_29_melted_fam_summed$abund_family_summed[test_29_melted_fam_summed$days_posttransplant==35], type="bar", width = 4, color = family, marker = list(color = ~mapColours$colours), yaxis ="y", showlegend =FALSE)  

b_29_comb
```

```
## No trace type specified:
##   Based on info supplied, a 'scatter' trace seems appropriate.
##   Read more about this trace type -> https://plot.ly/r/reference/#scatter
```

## **Plot for patient 16:**

```
test_16 <- dti_3[dti_3$Patient_ID == "P16",]

#putting xaxis= dict(range=[-35, 210]) in the plot doesn't work for some reason, work around by subsetting

test_16 <- subset(test_16, days_posttransplant>-36 &  days_posttransplant<100) 

#vline data: 

vline.dat_melt_1_P16 <- vline.dat_melt_1[vline.dat_melt_1$Patient_ID == "P16",]  


#now we want to melt the otu data into 2 colums, one for vst count (abund_family) and one for taxonomy (family)

test_16_melted_fam <- reshape2::melt(test_16, id.vars = colnames(test_16[,c(1:463)]), measure.vars = colnames(test_16[,c(464:506)]), value.name = "abund_family", variable.name = "family")
 

#rename the less relevant families to "other" to colorcode only a few

main_families <- c("Lachnospiraceae", "Ruminococcaceae","Erysipelotrichaceae", "Lactobacillaceae", "Staphylococcaceae", "Enterobacteriaceae", "Erysipelotrichaceae", "Streptococcaceae", "Enterococcaceae")

levels(test_16_melted_fam$family)[!(levels(test_16_melted_fam$family) %in% main_families)] <- c("Other")


#now we want to agglomerate/sum the abundances of all "other" families that are less relevant

test_16_melted_fam_summed <- test_16_melted_fam %>% dplyr::group_by(Patient_ID, feces_sample, days_posttransplant, family) %>% dplyr::summarise(abund_family_summed = sum(abund_family, na.rm = TRUE))


test_16_melted_fam_summed<-as.data.frame(test_16_melted_fam_summed)

test_16_melted_fam_summed <- test_16_melted_fam_summed[order(match(test_16_melted_fam_summed$family,mapColours$family)),]


b_16_comb <- plot_ly(data = test_16 , x = ~days_posttransplant, width = 800)                                                                                                                      %>%  add_trace(data = test_16, x = ~days_posttransplant, y = ~InvSimpson, type = "scatter", mode = "lines+markers", color=I("#00868B"), connectgaps = TRUE, yaxis = "y2", showlegend =FALSE)                                                                                                                                                                                                                                          %>%  add_trace(x = ~days_posttransplant, y = ~hBD2_plasma_pg_ml, yaxis = "y4", color = I("#EEC900"), type = "scatter", mode = "lines+markers", connectgaps = TRUE, showlegend =FALSE) %>%  add_trace(x = ~days_posttransplant, y = ~mono, yaxis = "y5", color = I("#404040"),type = "scatter", mode = "lines+markers", connectgaps = TRUE, showlegend =FALSE)                                                                                                                           %>% add_trace(x = ~vline.dat_melt_1_P16$days_posttransplant[vline.dat_melt_1_P16$variable=="GvHD_onset"], y= c(0,1), yaxis="y", color = I("red"), type="scatter", mode = "lines", showlegend =FALSE)                                                                                                                     %>% add_trace(x = ~vline.dat_melt_1_P16$days_posttransplant[vline.dat_melt_1_P16$variable=="GvHD_onset"], y= 0.9, color = I("red"), text="GvHD onset", mode = "text", textposition = "top right", yaxis = "y", showlegend =FALSE)                                                                                                                                                                                                                                  %>% add_trace(x = 0, y= c(0, 1), color = I("gray"), type="scatter", mode = "lines", line=list(dash = "dash"), showlegend =FALSE, yaxis = "y5")                                                                                                                     %>%layout(barmode = "stack", yaxis = list(range = c(0,1), title = "", zeroline = FALSE, showline = FALSE, showticklabels = FALSE, showgrid = FALSE, side = "right"),  yaxis2 = list( showline = FALSE, side = "left", title = "InvSimpson",color ="#00868B", overlaying = "y"),yaxis4 = list( showline = FALSE, side = "right", overlaying = "y",title = "hBD2",color = "#EEC900"),yaxis5 = list(showline = FALSE, side = "right", overlaying = "y", position = 1, title = "Monocytes", anchor = "free", color = "#404040"), xaxis = list(autotick=FALSE, showline = FALSE, zeroline = FALSE, dtick = 7, title = "days posttransplant", ticklen=0), showlegend =FALSE, margin = list(pad = 45, b = 90, l = 150, r = 90), legend = list(orientation ="v"))
 

b_16_comb <- b_16_comb %>% add_trace(data= test_16_melted_fam_summed, x=test_16_melted_fam_summed$days_posttransplant[test_16_melted_fam_summed$days_posttransplant==2],  y=test_16_melted_fam_summed$abund_family_summed[test_16_melted_fam_summed$days_posttransplant==2], type="bar", width = 4, color = family, marker = list(color = ~mapColours$colours), yaxis ="y ", showlegend =TRUE) 


b_16_comb
```

```
## No trace type specified:
##   Based on info supplied, a 'scatter' trace seems appropriate.
##   Read more about this trace type -> https://plot.ly/r/reference/#scatter
```

## **Plot for patient 22:**

```
test_22 <- dti_3[dti_3$Patient_ID == "P22",]

#putting xaxis= dict(range=[-35, 210]) in the plot doesn't work for some reason, work around by subsetting

test_22 <- subset(test_22, days_posttransplant>-36 &  days_posttransplant<100) 

#vline data: 

vline.dat_melt_1_P22 <- vline.dat_melt_1[vline.dat_melt_1$Patient_ID == "P22",]  

#now we want to melt the otu data into 2 colums, one for vst count (abund_family) and one for taxonomy (family)

test_22_melted_fam <- reshape2::melt(test_22, id.vars = colnames(test_22[,c(1:463)]), measure.vars = colnames(test_22[,c(464:506)]), value.name = "abund_family", variable.name = "family")
 

#rename the less relevant families to "other" to colorcode only a few

main_families <- c("Lachnospiraceae", "Ruminococcaceae","Erysipelotrichaceae", "Lactobacillaceae", "Staphylococcaceae", "Enterobacteriaceae", "Erysipelotrichaceae", "Streptococcaceae", "Enterococcaceae")

levels(test_22_melted_fam$family)[!(levels(test_22_melted_fam$family) %in% main_families)] <- c("Other")


#now we want to agglomerate/sum the abundances of all "other" families that are less relevant

test_22_melted_fam_summed <- test_22_melted_fam %>% dplyr::group_by(Patient_ID, feces_sample, days_posttransplant, family) %>% dplyr::summarise(abund_family_summed = sum(abund_family, na.rm = TRUE))


test_22_melted_fam_summed<-as.data.frame(test_22_melted_fam_summed)

#order for colorcoding
#test_22_melted_fam_summed <- arrange(test_22_melted_fam_summed, family)

#assign colors to genera (extrapolate pallet) (Here the top 25 are already over the whole data set and not per patient)

test_22_melted_fam_summed <- test_22_melted_fam_summed[order(match(test_22_melted_fam_summed$family,mapColours$family)),]

b_22_comb <- plot_ly(data = test_22 , x = ~days_posttransplant, width = 800)                                                                                                                      %>%  add_trace(data = test_22, x = ~days_posttransplant, y = ~InvSimpson, type = "scatter", mode = "lines+markers", color=I("#00868B"), connectgaps = TRUE, yaxis = "y2", showlegend =FALSE)                                                                                                                                                                                                                                          %>%  add_trace(x = ~days_posttransplant, y = ~hBD2_plasma_pg_ml, yaxis = "y4", color = I("#EEC900"), type = "scatter", mode = "lines+markers", connectgaps = TRUE, showlegend =FALSE) %>%  add_trace(x = ~days_posttransplant, y = ~mono, yaxis = "y5", color = I("#404040"),type = "scatter", mode = "lines+markers", connectgaps = TRUE, showlegend =FALSE)                                                                                                                           %>% add_trace(x = ~vline.dat_melt_1_P22$days_posttransplant[vline.dat_melt_1_P22$variable=="GvHD_onset"], y= c(0,1), yaxis="y", color = I("red"), type="scatter", mode = "lines", showlegend =FALSE)                                                                                                                     %>% add_trace(x = ~vline.dat_melt_1_P22$days_posttransplant[vline.dat_melt_1_P22$variable=="GvHD_onset"], y= 0.9, color = I("red"), text="GvHD onset", mode = "text", textposition = "top right", yaxis = "y", showlegend =FALSE)                                                                                                                                                                                                                                  %>% add_trace(x = 0, y= c(0, 1), color = I("gray"), type="scatter", mode = "lines", line=list(dash = "dash"), showlegend =FALSE, yaxis = "y5")                                                                                                                     %>%layout(barmode = "stack", yaxis = list(range = c(0,1), title = "", zeroline = FALSE, showline = FALSE, showticklabels = FALSE, showgrid = FALSE, side = "right"),  yaxis2 = list( showline = FALSE, side = "left", title = "InvSimpson",color ="#00868B", overlaying = "y"),yaxis4 = list( showline = FALSE, side = "right", overlaying = "y",title = "hBD2",color = "#EEC900"),yaxis5 = list(showline = FALSE, side = "right", overlaying = "y", position = 1, title = "Monocytes", anchor = "free", color = "#404040"), xaxis = list(autotick=FALSE, showline = FALSE, zeroline = FALSE, dtick = 7, title = "days posttransplant", ticklen=0), showlegend =FALSE, margin = list(pad = 45, b = 90, l = 150, r = 90), legend = list(orientation ="v"))
 

b_22_comb <- b_22_comb %>% add_trace(data= test_22_melted_fam_summed, x=test_22_melted_fam_summed$days_posttransplant[test_22_melted_fam_summed$days_posttransplant==-18],  y=test_22_melted_fam_summed$abund_family_summed[test_22_melted_fam_summed$days_posttransplant==-18], type="bar", width = 4, color = family, marker = list(color = ~mapColours$colours), yaxis ="y ", showlegend =TRUE) %>% add_trace(data= test_22_melted_fam_summed, x=test_22_melted_fam_summed$days_posttransplant[test_22_melted_fam_summed$days_posttransplant==2],  y=test_22_melted_fam_summed$abund_family_summed[test_22_melted_fam_summed$days_posttransplant==2], type="bar", width = 4, color = family, marker = list(color = ~mapColours$colours), yaxis ="y", showlegend =FALSE) %>% add_trace(data= test_22_melted_fam_summed, x=test_22_melted_fam_summed$days_posttransplant[test_22_melted_fam_summed$days_posttransplant==30],  y=test_22_melted_fam_summed$abund_family_summed[test_22_melted_fam_summed$days_posttransplant==30], type="bar", width = 4, color = family, marker = list(color = ~mapColours$colours), yaxis ="y", showlegend =FALSE)  

b_22_comb
```

```
## No trace type specified:
##   Based on info supplied, a 'scatter' trace seems appropriate.
##   Read more about this trace type -> https://plot.ly/r/reference/#scatter
```

## **Calculate average Lactobacillaceae abundances pre and post aGvHD onset:**

### For the patients who died:

```
pre26 <- test_26_melted_fam_summed$abund_family_summed[test_26_melted_fam_summed$family == "Lactobacillaceae" & test_26_melted_fam_summed$days_posttransplant < vline.dat_melt_1_P26$days_posttransplant[vline.dat_melt_1_P26$variable=="GvHD_onset"] & ! is.na(test_26_melted_fam_summed$feces_sample)]

post26 <- test_26_melted_fam_summed$abund_family_summed[test_26_melted_fam_summed$family == "Lactobacillaceae" & test_26_melted_fam_summed$days_posttransplant > vline.dat_melt_1_P26$days_posttransplant[vline.dat_melt_1_P26$variable=="GvHD_onset"] & ! is.na(test_26_melted_fam_summed$feces_sample)]


pre24 <- test_24_melted_fam_summed$abund_family_summed[test_24_melted_fam_summed$family == "Lactobacillaceae" & test_24_melted_fam_summed$days_posttransplant < vline.dat_melt_1_P24$days_posttransplant[vline.dat_melt_1_P24$variable=="GvHD_onset"] & ! is.na(test_24_melted_fam_summed$feces_sample)]

post24 <- test_24_melted_fam_summed$abund_family_summed[test_24_melted_fam_summed$family == "Lactobacillaceae" & test_24_melted_fam_summed$days_posttransplant > vline.dat_melt_1_P24$days_posttransplant[vline.dat_melt_1_P24$variable=="GvHD_onset"] & ! is.na(test_24_melted_fam_summed$feces_sample)]


pre30 <- test_30_melted_fam_summed$abund_family_summed[test_30_melted_fam_summed$family == "Lactobacillaceae" & test_30_melted_fam_summed$days_posttransplant < vline.dat_melt_1_P30$days_posttransplant[vline.dat_melt_1_P30$variable=="GvHD_onset"] & ! is.na(test_30_melted_fam_summed$feces_sample)]

post30 <- test_30_melted_fam_summed$abund_family_summed[test_30_melted_fam_summed$family == "Lactobacillaceae" & test_30_melted_fam_summed$days_posttransplant > vline.dat_melt_1_P30$days_posttransplant[vline.dat_melt_1_P30$variable=="GvHD_onset"] & ! is.na(test_30_melted_fam_summed$feces_sample)]

pre_died <- c(pre26, pre24, pre30)
post_died <- c(post26, post24, post30)

summary(pre_died)
```

```
##     Min.  1st Qu.   Median     Mean  3rd Qu.     Max. 
## 0.002455 0.004591 0.068844 0.098766 0.114012 0.378309
```

```
summary(post_died)
```

```
##     Min.  1st Qu.   Median     Mean  3rd Qu.     Max. 
## 0.002235 0.833824 0.910569 0.729232 0.929112 0.970419
```

### For the patients who survived:

```
pre29 <- test_29_melted_fam_summed$abund_family_summed[test_29_melted_fam_summed$family == "Lactobacillaceae" & test_29_melted_fam_summed$days_posttransplant < vline.dat_melt_1_P29$days_posttransplant[vline.dat_melt_1_P29$variable=="GvHD_onset"] & ! is.na(test_29_melted_fam_summed$feces_sample)]

post29 <- test_29_melted_fam_summed$abund_family_summed[test_29_melted_fam_summed$family == "Lactobacillaceae" & test_29_melted_fam_summed$days_posttransplant > vline.dat_melt_1_P29$days_posttransplant[vline.dat_melt_1_P29$variable=="GvHD_onset"] & ! is.na(test_29_melted_fam_summed$feces_sample)]


pre16 <- test_16_melted_fam_summed$abund_family_summed[test_16_melted_fam_summed$family == "Lactobacillaceae" & test_16_melted_fam_summed$days_posttransplant < vline.dat_melt_1_P16$days_posttransplant[vline.dat_melt_1_P16$variable=="GvHD_onset"] & ! is.na(test_16_melted_fam_summed$feces_sample)]

post16 <- test_16_melted_fam_summed$abund_family_summed[test_16_melted_fam_summed$family == "Lactobacillaceae" & test_16_melted_fam_summed$days_posttransplant > vline.dat_melt_1_P16$days_posttransplant[vline.dat_melt_1_P16$variable=="GvHD_onset"] & ! is.na(test_16_melted_fam_summed$feces_sample)]


pre22 <- test_22_melted_fam_summed$abund_family_summed[test_22_melted_fam_summed$family == "Lactobacillaceae" & test_22_melted_fam_summed$days_posttransplant < vline.dat_melt_1_P22$days_posttransplant[vline.dat_melt_1_P22$variable=="GvHD_onset"] & ! is.na(test_22_melted_fam_summed$feces_sample)]

post22 <- test_22_melted_fam_summed$abund_family_summed[test_22_melted_fam_summed$family == "Lactobacillaceae" & test_22_melted_fam_summed$days_posttransplant > vline.dat_melt_1_P22$days_posttransplant[vline.dat_melt_1_P22$variable=="GvHD_onset"] & ! is.na(test_22_melted_fam_summed$feces_sample)]

pre_surv <- c(pre29, pre16, pre22)
post_surv <- c(post29, post16, post22)

summary(pre_surv)
```

```
##     Min.  1st Qu.   Median     Mean  3rd Qu.     Max. 
## 0.003513 0.282571 0.567612 0.531834 0.816875 0.988602
```

```
summary(post_surv)
```

```
##      Min.   1st Qu.    Median      Mean   3rd Qu.      Max. 
## 0.0000000 0.0008144 0.0016288 0.1824786 0.2737179 0.5458069
```
